# Multiomics of Bohring-Opitz syndrome truncating *ASXL1* mutations identify canonical and noncanonical Wnt signaling dysregulation

**DOI:** 10.1172/jci.insight.167744

**Published:** 2023-05-22

**Authors:** Isabella Lin, Angela Wei, Zain Awamleh, Meghna Singh, Aileen Ning, Analeyla Herrera, REACH Biobank and Registry, Bianca E. Russell, Rosanna Weksberg, Valerie A. Arboleda

**Affiliations:** 1 Department of Human Genetics,; 2 Department of Pathology and Laboratory Medicine, David Geffen School of Medicine, UCLA, Los Angeles, California, USA.; 3 Department of Computational Medicine, UCLA, Los Angeles, California, USA.; 4 Interdepartmental BioInformatics Program, UCLA, Los Angeles, California, USA.; 5 Department of Genetics and Genome Biology Program, Research Institute, The Hospital for Sick Children, Toronto, Ontario, Canada.; 6 Division of Genetics, Department of Pediatrics, UCLA, Los Angeles, California, USA.; 7 Division of Clinical & Metabolic Genetics, The Hospital for Sick Children, Toronto, Ontario, Canada.; 8 Institute of Medical Sciences and Department of Molecular Genetics, University of Toronto, Toronto, Ontario, Canada.; 9 Molecular Biology Institute, UCLA, Los Angeles, California, USA.; 10 Jonsson Comprehensive Cancer Center, UCLA, Los Angeles, California, USA.

**Keywords:** Development, Genetics, Epigenetics, Genetic diseases, Leukemias

## Abstract

ASXL1 (additional sex combs–like 1) plays key roles in epigenetic regulation of early developmental gene expression. De novo protein-truncating mutations in *ASXL1* cause Bohring-Opitz syndrome (BOS; OMIM #605039), a rare neurodevelopmental condition characterized by severe intellectual disabilities, distinctive facial features, hypertrichosis, increased risk of Wilms tumor, and variable congenital anomalies, including heart defects and severe skeletal defects giving rise to a typical BOS posture. These BOS-causing *ASXL1* variants are also high-prevalence somatic driver mutations in acute myeloid leukemia. We used primary cells from individuals with BOS (*n* = 18) and controls (*n* = 49) to dissect gene regulatory changes caused by *ASXL1* mutations using comprehensive multiomics assays for chromatin accessibility (ATAC-seq), DNA methylation, histone methylation binding, and transcriptome in peripheral blood and skin fibroblasts. Our data show that regardless of cell type, *ASXL1* mutations drive strong cross-tissue effects that disrupt multiple layers of the epigenome. The data showed a broad activation of canonical Wnt signaling at the transcriptional and protein levels and upregulation of *VANGL2*, which encodes a planar cell polarity pathway protein that acts through noncanonical Wnt signaling to direct tissue patterning and cell migration. This multiomics approach identifies the core impact of *ASXL1* mutations and therapeutic targets for BOS and myeloid leukemias.

## Introduction

**Figure 1:**
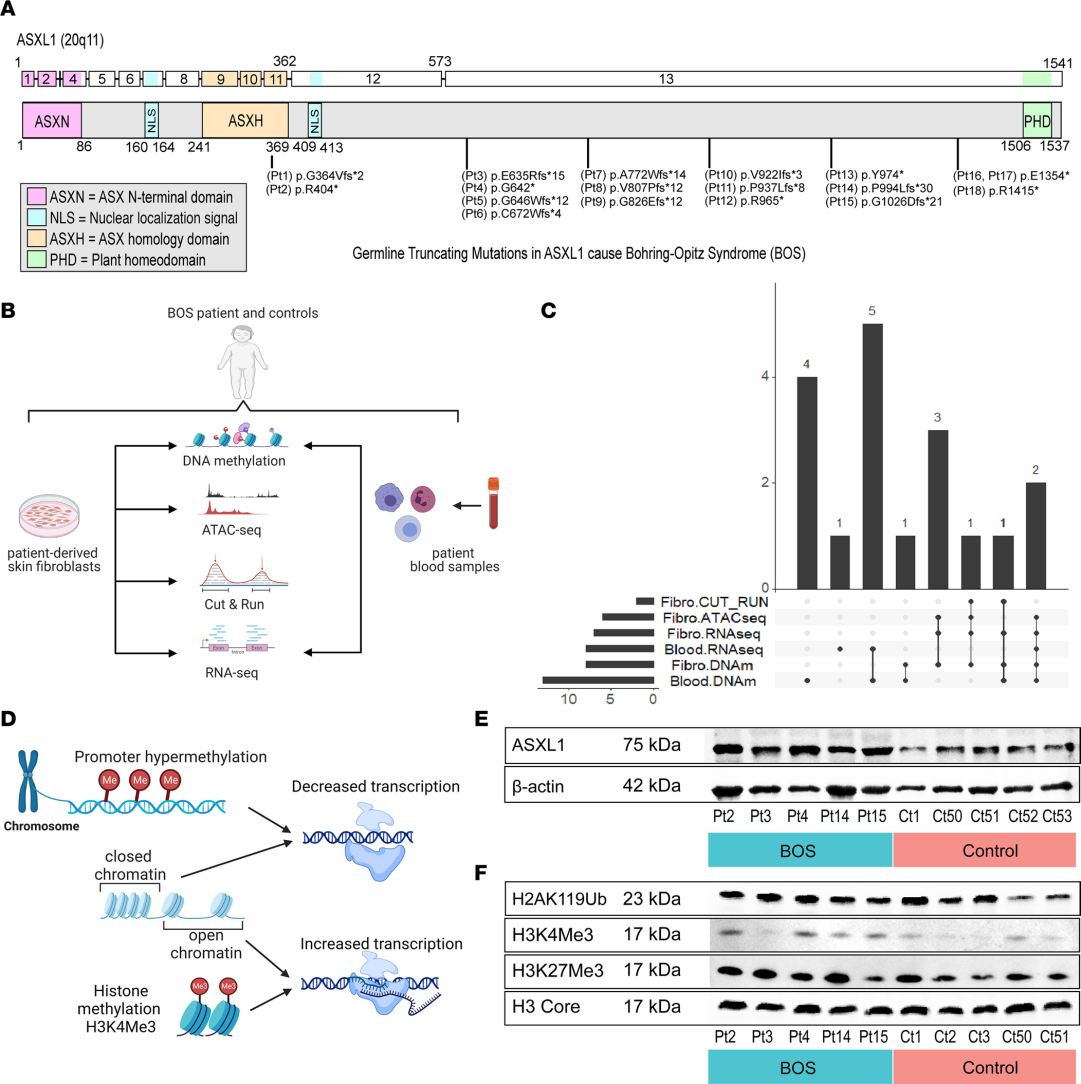
Multiomics study design for Bohring-Opitz syndrome (BOS) caused by pathogenic mutations in *ASXL1*. (**A**) Schematic representation of the ASXL1 transcript (ENST00000375687.10) and protein (GenBank: NM_015338.6; GRCh37), its functional domains, and mutations causing BOS. Mutations listed correspond to patients in this study and are tagged with a Pt identifier. (**B**) Peripheral blood and dermal fibroblasts were collected and underwent epigenomic assays for ATAC-seq, CUT&RUN, and DNA methylation, and global transcriptome analysis using RNA-seq. (**C**) Across the multiomics assays and 2 specimen types, we had 8 of 18 BOS samples with fibroblast assays and 14 of 18 with blood assays. Four of 18 BOS patients had data from assays across both specimen types. (**D**) Promoter hypermethylation and closed chromatin, which can be examined with DNA methylation and ATAC-seq analysis, respectively, are associated with decreased transcription, while activating histone methylation such as H3K4Me3 at promoters and open chromatin are associated with increased transcription. (**E**) Western blot for representative BOS (*n* = 5) and representative control (*n* = 5) fibroblast whole-cell lysate extracts shows no significant difference in total ASXL1 protein. This was repeated 3 times. (**F**) Western blot for representative BOS (*n* = 5) and representative control (*n* = 5). Fibroblast histone extracts showed no significant difference in H2AK119ub, H3K4me3, and H3K27me3. This was repeated 3 times.

ASXL1 (additional sex combs–like 1) is an essential protein in embryonic development (1). Eutherian mammals have 3 ASX homologs, ASXL1, ASXL2, and ASXL3, which share conserved domains: the ASX N-terminal (ASXN) domain with a HARE-HTH domain involved in putative DNA binding (2), the ASX homology (ASXH) domain with a characteristic LXXLL motif that mediates protein interactions and a DEUBAD domain that activates BRCA1-associated protein 1 (BAP1), and the C-terminal plant homeodomain (PHD) important in reading posttranslational histone modifications such as histone H3 Lys4 trimethylation (H3K4me3) (3–5) (Figure 1A). ASXL1 plays a role in the modulation of the epigenetic landscape that regulates downstream transcription. The ASXH and PHD domains are highly conserved and are part of large epigenetic complexes, the polycomb (PcG) complexes and the deubiquitinase complexes. These epigenetic complexes direct genome-wide transcriptional regulation (3, 6, 7) through activation or repression of essential developmental genes. *Asx*, the *Drosophila melanogaster* ortholog, plays a critical role in anterior-posterior patterning (8) and mutant embryos exhibit incomplete head involution and posterior-directed transformations of all abdominal segments (8). In mammals, *ASXL1* is essential in early neural crest (9), cardiac (10), and hematopoietic development (11, 12).

ASXL1 directs histone modifications through polycomb repressive complex 1 and 2 (PRC1/-2) and the polycomb repressive deubiquitinase (PR-DUB) complex. PRC1 and -2 control activation of developmental stemness and differentiation, including progressive restriction of neural progenitor cell multipotency and production of mature cortical neurons during corticogenesis (13). This control of the stem-cell state occurs through monoubiquitination at histone H2A lysine 119 (H2AK119ub) via PRC1 and mono-, di-, and trimethylation at histone H3K27 (H3K27me, H3K27me2, H3K27me3) via PRC2 (3, 14, 15). Together, these complexes regulate key signaling pathways such as Wnt signaling in tissue-specific and developmental contexts, with PRC2 accessory proteins regulating Wnt signaling during erythropoiesis (16) and PRC1 regulating the Wnt/β-catenin pathway through a positive feedback loop in hepatocellular carcinoma (17).

The PR-DUB complex, which includes ASXL1 that binds BAP1 (18–20), plays key roles in brain development (21–23) and regulation of myeloid differentiation through H2AK119 deubiquitination (19, 24). While it is clear that *ASXL1* protein-truncating mutations disrupt core developmental processes across multiple organ systems, its role as an essential chromatin modifier in human development has not been fully elucidated.

### ASXL1 mutations in Bohring-Opitz syndrome and in hematologic malignancies.

De novo protein-truncating mutations of *ASXL1* cause a rare genetic disorder, Bohring-Opitz syndrome (BOS; OMIM #605039). BOS is characterized by profound intellectual disability, developmental delay, seizures, and variable anomalies that include heart defects, higher risk of Wilms tumor, and BOS posture (25, 26). As of 2018, only 46 clinically diagnosed individuals have been reported in the literature, with less than half (20/46) molecularly confirmed (25). Pathogenic truncating mutations in *ASXL1* are enriched in exons 12 and 13, the penultimate and ultimate exons (3), resulting in premature truncation of the highly conserved C-terminal PHD domain. Intriguingly, truncation mutations in *ASXL1* homologs *ASXL2* (Shashi-Pena syndrome, OMIM #6171901) (27) and *ASXL3* (Bainbridge-Ropers syndrome, OMIM #615485) (23, 28) are not enriched or clustered in the homologous C-terminal region despite sharing multiple conserved domains (3).

The same protein-truncating mutations that cause BOS are also observed as somatic driver mutations in myeloid malignancies such as chronic myelomonocytic leukemia (~45%), myelodysplastic syndromes (MDS, 16%), myeloproliferative neoplasms (~10%), and acute myeloid leukemia (secondary 30%, de novo 6.5%) (29–32). Studies have identified low-frequency somatic mutations in *ASXL1* correlated with mutagenic processes and increasing age (33), defining a new premalignancy state called clonal hematopoiesis of indeterminate potential (33, 34). ASXL1 regulates the delicate interplay between proliferation and differentiation of stem progenitor cell populations (9, 35) and therefore, both germline and somatic *ASXL1* mutations disrupt the proliferation-differentiation balance and promote stem-cell identity over differentiation in BOS and myeloid leukemias.

Recent ASXL1 functional studies have been carried out as transgenic overexpression of mutant and wild-type ASXL1 proteins in cell-line systems. These studies are difficult to interpret because they are not reflective of endogenous levels or cell-type-specific functions of ASXL1 or they are in nonhuman model systems (36). Given the multiple essential functions of ASXL1 as part of PRC1 and PRC2 in neural progenitor multipotency (13), and the key role of the PR-DUB complex in brain development (21–23), we hypothesized that protein-truncating mutations of *ASXL1* dysregulate global transcription, cellular homeostasis, and downstream signaling pathways.

We also examined whether BOS cells harbored decreased mRNA or protein levels since protein-truncating *ASXL1* mutations occur in the last 2 exons of the gene, exons 12 and 13, and are predicted to escape nonsense-mediated decay (37). These truncated proteins are predicted to have a gain-of-function effect (38). The broad phenotypic effects of *ASXL1* mutations in BOS patients and myeloid malignancies suggest that ASXL1 drives essential and core gene regulatory features across early development and disease. We hypothesized that *ASXL1* mutations share common cellular effects that might therefore be detectable as broad and high-effect epigenomic and transcriptomic signatures across cells and tissues.

To study BOS molecular pathogenesis, we collected samples from 18 patients with BOS, one of the largest ASXL1 rare disease cohorts published, and performed multiomics analysis. Our approach circumvents the confounding variable of cancer-derived cell models that harbor multiple additional karyotypic and genomic mutations (31, 39) and focuses our study on the singular effect of pathogenic *ASXL1* mutations. This integrated multiomics study characterizes the impact of *ASXL1* truncating mutations on the epigenome and transcriptome and our work shows for the first time to our knowledge that *ASXL1* mutations aberrantly activate the Wnt signaling pathway and noncanonical Wnt planar cell polarity (PCP) genes.

### Canonical Wnt signaling pathway.

Canonical Wnt signaling is an evolutionarily conserved signaling pathway that is essential for development (40), and plays important roles in stem cell biology and regulation of hematopoiesis (41). In the Wnt-active state, WNTs (translated products of the *WNT* gene) bind to the transmembrane receptor Frizzled (FZD) and stimulate the coreceptor low density receptor-related protein 5 and 6 (LRP5 and -6), which play critical roles in initiation of Wnt signaling transduction (42). Activation of FZD and LRP5/6 recruits and inactivates the destruction complex that targets phosphorylation, ubiquitination, and degradation of β-catenin through the proteasome. Inactivation of the destruction complex occurs through dissociation of glycogen synthase kinase 3β (GSK3B) from the inhibition protein AXIN, and concurrent inactivation of the default quiescent-state β-catenin destruction complex (43–45). This means that β-catenin cannot be phosphorylated, and thus cannot be degraded (46, 47). The activation of the canonical Wnt pathway results in cytoplasmic accumulation and subsequent nuclear translocation of β-catenin that, along with T cell factor/lymphoid enhancer factor (TCF/LEF) transcription factors, transcriptionally coactivates Wnt target genes (48).

To examine cross-omics dysregulation in more depth, we explored van Gogh–like 2 (VANGL2), a noncanonical Wnt and PCP protein, which was highly dysregulated across all the -omics assays conducted across blood and fibroblast BOS samples. VANGL2 regulates polarized cellular migration and differentiation and tissue morphology during development. In the nervous system, the PCP pathway regulates neuronal maturation and has a functional role in neural complex formation (49). VANGL2 intersects with the canonical pathway through activation of Dishevelled (DVL), which activates the noncanonical pathways and drives c-JUN–mediated expression. We hypothesized that upregulation of *VANGL2* disrupts canonical and noncanonical signaling pathways and underlies the clinical phenotypes observed in patients with BOS and other mutations in *ASXL*. To date, no studies to our knowledge have established a link between *ASXL1* disorders and dysregulation of Wnt signaling.

In this study, we identify and test the mechanisms by which truncating *ASXL1* mutations in BOS may dysregulate the canonical and noncanonical Wnt signaling pathways. These findings elucidate what we believe are novel regulatory mechanisms underlying BOS pathogenesis.

## Results

### Study design.

We recruited 18 individuals with BOS (Supplemental Table 1; supplemental material available online with this article; https://doi.org/10.1172/jci.insight.167744DS1), 25 sex-matched and genetically related controls to account for genetic variation, and 30 sex- and age- matched controls (Supplemental Table 2). This is the largest cohort of BOS patients studied at the molecular level, and includes patients first reported in 2023 by Russell et al. (50). All individuals with BOS have clinically identified *ASXL1* mutations in the last 2 exons of the gene that are predicted to cause protein-truncating variants (NM_015338.6) between amino acids 364 and 1415 (Figure 1A and Supplemental Table 1). All mutations were verified through bulk RNA sequencing (RNA-seq) data visualization in IGV (https://www.ncbi.nlm.nih.gov/pmc/articles/PMC3346182/) (Supplemental Figure 1) to confirm specimen identity. A summary of patient demographics is provided in Supplemental Table 3. Clinical data of this cohort are available through the ASXL registry (https://www.uclahealth.org/medical-services/pediatric-genetics/clinical-services/ucla-asxl-related-disorders-and-chromatinopathies-clinic), and a subset of patients were previously reported by Awamleh et al. (51).

Consenting individuals donated peripheral blood (*n* = 14), a skin punch biopsy (*n* = 8), or both. We obtained both sample types from 4 of the 18 individuals with BOS in this study, which allowed us to integrate data across sample types to identify tissue-dependent and -independent dysregulation caused by truncating *ASXL1* mutations. We used primary sample types to examine the direct role of the mutation on patient tissue. We used controls with similar genetic backgrounds to control for baseline genetic effects. We conducted a comprehensive multiomics analysis across tissues (Figure 1B). We assessed global changes to the epigenome using 3 approaches: chromatin accessibility using assay for transposase-accessible chromatin using sequencing (ATAC-seq) (52), H3K4me3 and H3K27me3 enrichment using cleavage under targets and release using nuclease (CUT&RUN) (53), and the DNA methylation (DNAm) landscape through Illumina Epic Arrays (51). We also assessed global changes to the transcriptome using RNA-seq. Not all samples were assessed by all methods and therefore the overlap between assays for individuals with BOS is outlined in Figure 1C. We derived disease-specific DNAm signatures (51) and transcriptional signatures from RNA-seq using blood and fibroblast cells (Figure 1B), and integrated our findings to examine the transomics dysregulation (Figure 1D).

### ASXL1 protein expression identifies no bulk differences of expression in BOS samples compared to controls.

*ASXL1* is expressed across multiple tissues and multiple cell types (Supplemental Table 4), consistent with the clinical phenotype spanning multiple organ systems. To test our hypothesis that *ASXL1* mutations may have high-level epigenomic and transcriptomic signatures across affected cells and tissues, we conducted RT-qPCR using *ASXL1* primers (Supplemental Table 5). We found no significant differences in *ASXL1* expression levels between representative BOS (*n* = 4) and control (*n* = 4) blood samples (Supplemental Figure 2A) or between representative BOS (*n* = 6) and control (*n* = 6) fibroblast samples (Supplemental Figure 2B). For blood samples, the RNA-seq *ASXL1* transcript absolute fold change [abs(log_2_FC)] was 0.11, which did not meet our threshold of 0.58 for a strong fold change, and the adjusted *P* value (*P*_adj_) was 0.29, which did not meet our significance threshold of *P*_adj_ less than 0.05 (Supplemental Figure 2C). Similarly, for fibroblasts, the abs(log_2_FC) was –0.01 and *P*_adj_ was 0.95 (Supplemental Figure 2D).

We next asked whether the truncating mutation caused differences in wild-type ASXL1 protein levels due to decreased translational efficiency or stability of the truncated allele. We identified a high-quality antibody by testing multiple, commercially available antibodies (Supplemental Table 6 and Supplemental Figure 3A) in CACO2 cells, a cell line with very high *ASXL1* RNA expression (Supplemental Table 4), and HEK293T cells transfected with FLAG-tagged truncated *ASXL1* (54). We identified 2 antibodies (ab228009, MABE1933) that showed bands at the same molecular weight of 75 kDa, one of which (ab228009) showed higher expression of ASXL1 in CACO2 cells and overlapped with the ASXL1 construct probed with a high-affinity FLAG antibody (Supplemental Figure 3B). Western blotting for ASXL1 identified no significant difference in total ASXL1 protein levels between BOS (*n* = 5) and control (*n* = 5) fibroblasts (Figure 1E, Supplemental Figure 4, and Supplemental Table 7; see complete unedited blots in the supplemental material). Western blot bands for ASXL1 were detected at the same molecular weight between BOS and control samples, and truncated proteins were not detected at a reduced molecular weight for any BOS sample tested. This is consistent with other literature examining endogenous, truncated human ASXL1 expression (55).

Finally, we extracted histones from fibroblast cells and quantified global changes for histone modification. The mutations in BOS terminate ASXL1 before the PHD domain, a histone- or DNA-binding domain reported to recognize a subset of histone modifications, including H3K4me and H3K27me3 (5), and thought to affect levels of H3K27me3 and H2AK119ub (19). We performed histone immunoblotting for these specific modifications using representative BOS (*n* = 5) and control (*n* = 5) fibroblasts and did not observe any significant changes to H3K4me3, H3K27me3, or H2AK119Ub levels (Figure 1F, Supplemental Figure 5, and Supplemental Table 7; see complete unedited blots in the supplemental material).

### Transcriptomic analysis identifies transcriptional pathways disrupted in BOS.

One of the core functions of ASXL1 is to activate the epigenome to express certain genes and pathways at developmental time points. Changes to the epigenome activate or repress transcription and RNA-seq can identify the effects of the transcriptional rewiring due to epigenetic mutations. To address this, we performed RNA-seq in blood and dermal fibroblasts from individuals with BOS and controls. Transcriptomic data were generated to a minimum of 30 million reads per sample for a total of 33 RNA-seq libraries: 8 BOS blood, 7 BOS fibroblasts, 11 control blood, and 7 control fibroblasts (Supplemental Tables 8 and 9, Methods, and Supplemental Methods. Similar to the RT-qPCR data, normalized *ASXL1* gene expression was not significantly different between BOS (*n* = 8) and control (*n* = 11) blood, or between BOS (*n* = 7) and control (*n* = 7) fibroblast samples (Supplemental Figure 2, C and D). We found that an increased number of *ASXL1* reads was correlated with total sequencing reads in both blood samples and fibroblast samples, as expected (Supplemental Figure 6A). Fibroblast samples had higher normalized *ASXL1* read counts per sequencing coverage compared with blood samples, which is expected given the *ASXL1* expression by tissue type (Supplemental Table 4).

We then examined whether the total number of reads at each BOS sample’s mutation site was correlated with total *ASXL1* reads (Supplemental Figure 6B). We identified a minimum of 1195 and a maximum of 5612 *ASXL1* reads per sample. Here, we found no significant correlation between *ASXL1* reads at mutation site and total *ASXL1* reads (*R*^2^ < 0.01 for blood samples and *R*^2^ = 0.10 for fibroblast samples). While patient 4 (Pt4) fibroblasts were found to have only 2 reads at the mutation site compared with a total of 3968 total *ASXL1* reads, Pt8 blood was found to have 100 reads at the mutation site compared with a total of 1314 *ASXL1* reads (Supplemental Tables 8–10).

**Figure 2:**
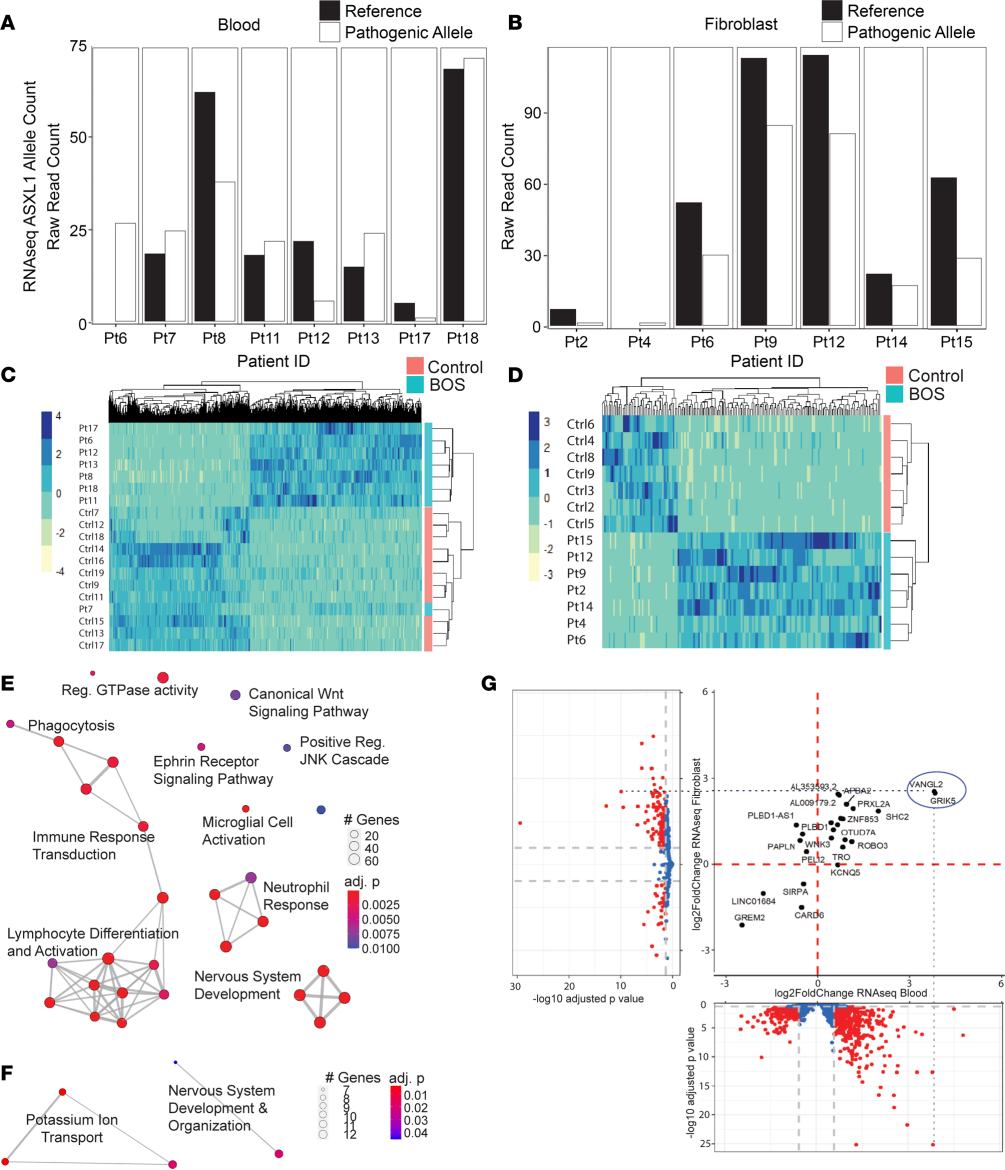
Pathogenic mutations in *ASXL1* cause tissue-specific and tissue-independent effects on gene expression. (**A**) RNA-seq raw read counts for the *ASXL1* reference allele (black) and pathogenic allele (white, with black outline) at each BOS patient’s respective mutation in blood (*n* = 8) and (**B**) fibroblast (*n* = 7) samples. (**C**) RNA-seq heatmap of all significant DEGs with *P*_adj_ < 0.05 and abs(log_2_FC) ≥ 0.58 between BOS (blue, *n* = 8) and control (pink, *n* = 11) blood found 1097 DEGs, with 590 of 1097 (53.8%) being more upregulated and (**D**) 155 DEGs between BOS (*n* = 7) and control (*n* = 7) fibroblasts, with 125 of 155 DEGs (80.6%) being more upregulated in BOS patients. (**E**) Blood RNA-seq gene ontology highlighted enrichment of genes related to nervous system development and canonical Wnt signaling pathway. (**F**) Fibroblast RNA-seq gene ontology highlighted enrichment of genes related to nervous system development. (**G**) Volcano plots for BOS compared to control RNA-seq in blood (*x* axis) and fibroblast (*y* axis) identified a core subset of 25 shared dysregulated transcripts, with 21 of 25 DEGs (84%) dysregulated in the same direction.

We also calculated whether the *ASXL1* reference and mutant alleles were equally represented in patient blood (Figure 2A, *n* = 8) and fibroblast (Figure 2B, *n* = 7) RNA-seq data. We calculated the variant allele frequency (VAF) at the mutation site across each BOS sample (Supplemental Figure 7). For germline disorders, we would expect the VAF of the mutant allele to represent between 30% and 70% of the reads covering the mutation site (0.3 < VAF < 0.7). Most BOS samples fell within the expected germline range (Figure 2, A and B, and Supplemental Table 10), suggesting that these late-truncating alleles escape nonsense-mediated decay.

One key exception to the expected germline allele distribution was Pt6, with a truncating mutation at amino acid 672 who had undergone treatment with actinomycin D and vincristine for Wilms tumor years prior to sample collection. In fibroblast RNA-seq, this sample had an *ASXL1* allele ratio of 36.9% pathogenic allele, which is within expected germline levels. However, in Pt6’s blood RNA-seq, 100% of reads (27/27) over the *ASXL1* mutation contained the pathogenic mutation and no reference allele was identified. This loss of heterozygosity may have occurred as a selective growth advantage during tumorigenesis, during treatment, or could represent new clonal hematopoiesis.

To assess the pathogenesis of truncating *ASXL1* mutations, we examined the effects on global transcription across different tissues. *ASXL1* is ubiquitously expressed across the body at low levels, and is more highly expressed in skin (32.55 TPM) than in whole blood (8.03 TPM) (Supplemental Table 4). We expected a much higher number of significantly differentially expressed genes (DEGs) in blood RNA-seq than in fibroblast RNA-seq because blood RNA represents a bulk assessment of heterogeneous cell types that can introduce an extra source of differential expression. We observed significantly more gene expression dysregulation in blood, with 2118 significant DEGs (Figure 2C), compared with fibroblast RNA-seq, with 177 significant DEGs (Figure 2D). Complete lists of DEGs in each data set are in Supplemental Table 11 for BOS fibroblasts and Supplemental Table 12 for BOS blood.

We identified an outlier in our RNA-seq analysis of blood samples: Pt7 (Figure 2C). Age 29 at sample collection (Supplemental Table 1), Pt7 was one of the very few older patients given that BOS is associated with high infant mortality (27%) and most patients do not survive into early adulthood (56). Given the extremely low prevalence of BOS and selection of donated samples, the sample size restricted our ability to independently assess Pt7 for effects of age. Furthermore, collecting samples from age-matched controls is difficult given that matched controls would be young, healthy children. We strove to correct for the effects of age through including an age-matched control, Ctrl19, at age 28 (Supplemental Table 2), integrating analyses across different -omics assays with a range of control group ages, and assessing significant genes against any age-specific bias. Furthermore, significant genes that were identified for RNA-seq blood analysis in this text were examined to see whether Pt7 had transcript expression more similar to other BOS cases before downstream analysis.

After filtering for fold change (abs[log_2_FC] ≥ 0.58), we found 1097 significant DEGs in blood and 155 significant DEGs in fibroblasts. Both RNA-seq analysis of blood and fibroblasts identified larger proportions of DEGs that are upregulated in BOS patients than in controls. In blood, 590 of 1097 DEGs (53.8%) were upregulated (Figure 2C), and in fibroblasts, 125 of 155 DEGs (80.6%) (Figure 2D). In blood, top DEGs included *GRIK5* (glutamate receptor, kainate 5; log_2_FC = 3.8), *VANGL2* (log_2_FC = 3.8), and *GREM2* (gremlin 2, BMP antagonist; log_2_FC = –2.5). Top DEGs in the fibroblast RNA-seq data set include *UGT3A2* (UDP glycosyltransferase family 3 member A2; log_2_FC = 4.8), *VANGL2* (log_2_FC = 2.5), *GRIK5* (log_2_FC = 2.5), and *GREM2* (log_2_FC = –2.1). Gene ontology (GO) analyses (see Supplemental Methods) were performed using the list of significant DEGs. Despite neither tissue being neural in origin, regulation of neuron projection development (*P*_adj_ = 9.82 × 10^–5^ in blood, *P*_adj_ = 0.02 in fibroblasts) was identified as a GO biological process enriched in both BOS tissues (Figure 2, E and F, and Supplemental Tables 13 and 14). These enrichments were driven by genes that play key roles in early morphogenesis, particularly neurodevelopmental processes.

Our findings from GO analyses in blood and fibroblasts also identified some gene expression changes that are tissue specific. Blood DEGs were enriched for hematological processes such as T cell activation (*P*_adj_ = 3.23 × 10^–8^), neutrophil activation (*P*_adj_ = 1.90 × 10^–5^), axogenesis (*P*_adj_ = 1.90 × 10^–5^), and leukocyte cell-cell adhesion (*P*_adj_ = 3.62 × 10^–5^) (Figure 2E and Supplemental Table 13). Fibroblast DEGs were enriched for structural cell processes such as dysregulation of potassium ion transport (*P*_adj_ = 0.004) and regulation of membrane potential (*P*_adj_ = 0.02) (Figure 2F and Supplemental Table 14).

To examine potential common, cross-tissue effects of truncating *ASXL1* mutations, we correlated all our BOS blood RNA-seq DEGs and all our BOS fibroblast RNA-seq DEGs to identify shared dysregulated transcripts (Figure 2G). We identified a core subset of 25 genes dysregulated across both tissue types, with 21 of 25 (84%) DEGs dysregulated in the same direction, suggesting a strong tissue-independent *ASXL1* effect that supersedes tissue type. Notable genes include *VANGL2*, *GRIK5*, and *GREM2*.

### Differential chromatin accessibility in BOS allows for aberrant activation of developmental and morphogenic pathways.

To correlate gene expression profiles with chromatin accessibility (Figure 1D), ATAC-seq was performed on BOS (*n* = 6) and control (*n* = 6) fibroblast cells. Chromatin accessibility, identified by increased reads over a genomic region in ATAC-seq, positively correlated with gene expression (52, 58).

**Figure 3:**
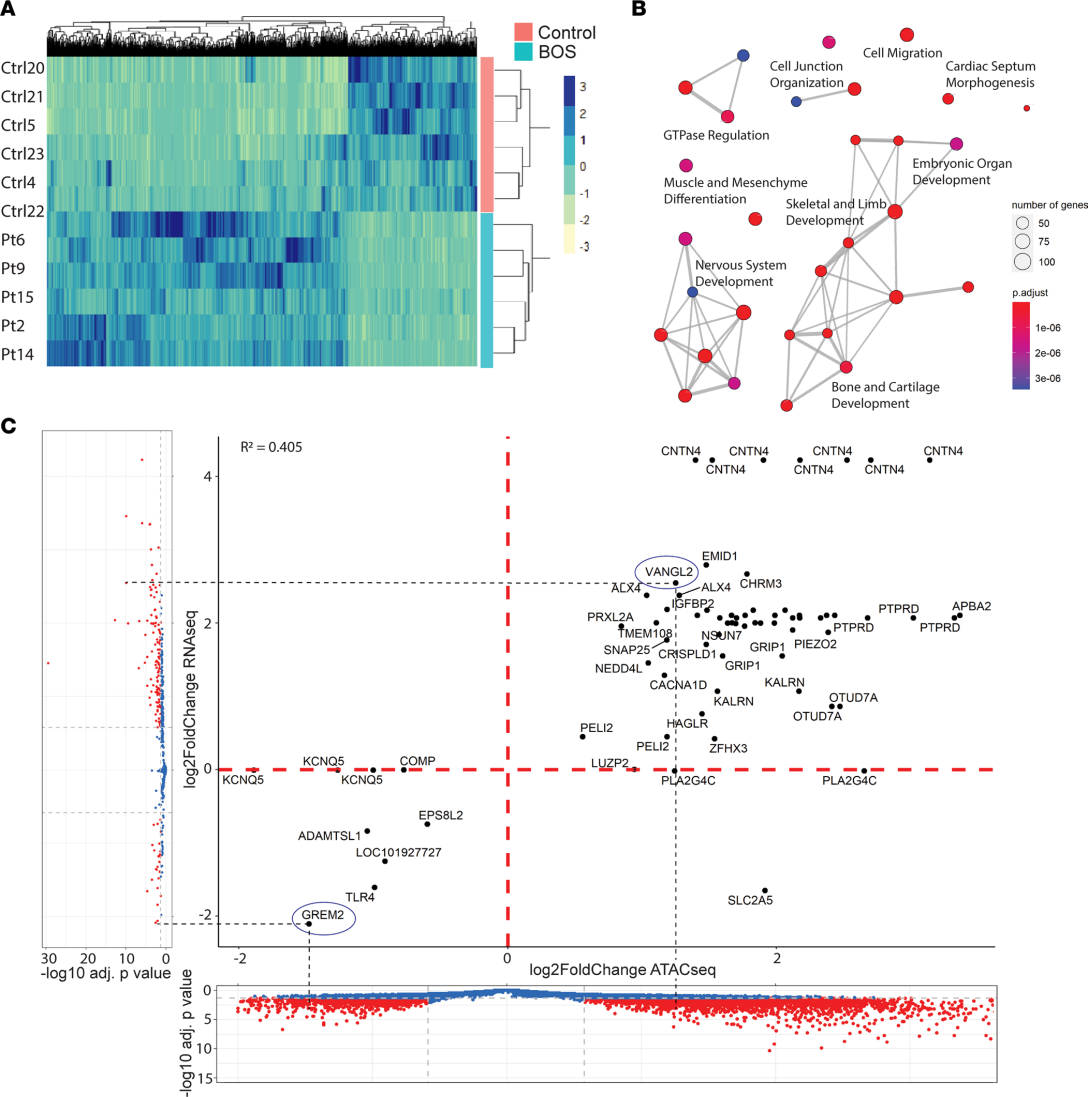
Epigenetic dysregulation in BOS patient–derived fibroblasts drives transcriptomic dysregulation. (**A**) ATAC-seq heatmap of all significant DEGs with *P*_adj_ < 0.05 and abs(log_2_FC) ≥ 0.58 between BOS (blue, *n* = 7) and control (pink, *n* = 7) fibroblasts found 4336 DEGs in fibroblasts, with 3036 of 4336 (70.0%) being more upregulated. (**B**) Gene set enrichment showed key dysregulated pathways, including nervous system development. (**C**) Integration of chromatin accessibility (ATAC-seq, *x* axis) and gene expression (RNA-seq, *y* axis) in BOS patient (*n* = 7) compared with control fibroblasts (*n* = 7) identified a positive correlation (*R*^2^ = 0.405) between chromatin accessibility and gene expression. We identified a set of 37 common dysregulated transcripts (right). DEGs were considered significant (red) for abs(log_2_FC) ≥ 0.58 and *P*_adj_ > 0.05.

All ATAC-seq libraries had the expected distribution of fragment lengths, with an average of 52% of fragments being small (<200 bp in length) representing open chromatin regions, and progressively fewer fragments of larger size spanning nucleosomes (>300 bp in length) (Supplemental Figure 8 and Supplemental Table 15). Of note, BOS patient fibroblasts had a mean insert size of 184 bp, representing more open chromatin regions, while control fibroblasts had a median insert size of 242 bp, representing less open chromatin regions. Principal component analysis (PCA) identified clear separation of BOS and control samples on PC1 (47% variance) (Supplemental Figure 9A), as did heatmap analysis of the significant peaks (Figure 3A). A total of 4336 significant peaks (*P*_adj>_0.05, abs[log_2_FC] > 0.58) corresponding to differentially accessible regions were identified, with 3036 peaks (70.02%) more differentially open and 1300 peaks (29.98%) more closed in BOS patients compared with controls (Supplemental Figure 9B and Supplemental Table 16). This mapped to a total of 3054 unique genes, with 763 of 3054 genes (25.0%) represented by more than one significant differentially accessible peak (Supplemental Table 17). These included *APBA2* (amyloid β precursor protein binding family A member 2) represented by 8 peaks, *KCNQ5* (potassium voltage-gated channel subfamily Q member 5) represented by 3 peaks, and *OTUD7A* (OTU deubiquitinase 7A), *PRXL2A* (peroxiredoxin like 2A), and *PELI2* (pellino E3 ubiquitin protein ligase family member 2) each represented by 2 peaks. Peaks were annotated using HOMER to the nearest gene or regulatory element (see Supplemental Methods) (57, 59). The top differentially accessible peaks mapped to *FBXL20* (log_2_FC = –8.3), *LINGO1* (log_2_FC = –7.2), *RUNX3* (log_2_FC = 4.4), and *CTNNB1* (gene encoding β-catenin; log_2_FC = 3.8).

We identified gene set enrichments in multiple key developmental systems, including muscle development and differentiation (*P*_adj_ = 9.11 × 10^–13^), skeletal system development (*P*_adj_ = 1.0 × 10^–9^), regulation of neurogenesis (*P*_adj_ = 1.35 × 10^–9^), limb morphogenesis (*P*_adj_ = 1.38 × 10^–8^), renal system development (*P*_adj_ = 2.14 × 10^–8^), and cardiac septum morphogenesis (*P*_adj_ = 2.67 × 10^–8^) (Figure 3B and Supplemental Table 18). Key motifs that were identified in the differentially accessible chromatin regions of our ATAC-seq data suggest core factors that drive dysregulation of the DEGs. Motif enrichment analysis of our ATAC-seq data using both known motif and de novo methods identified significant enrichment of the following transcription factor binding sites: JunB (*P*_adj_ = 1 × 10^–208^, BOS 18.43%, background 5.23%), RUNX1 (*P*_adj_ = 1 × 10^–203^, BOS 26.45%, background 10.09%), and Fra1 (*P*_adj_ = 1 × 10^–196^, BOS 17.44%, background 4.95%) (Supplemental Table 19).

### Chromatin accessibility is correlated with dysregulation of gene expression in BOS.

To determine whether some of the transcriptional dysregulation identified in BOS occurs through chromatin accessibility, we integrated DEGs from fibroblast RNA-seq (BOS *n* = 6, control *n* = 6) with differentially accessible chromatin regions identified through ATAC-seq (BOS *n* = 6, control *n* = 6). This revealed a positive correlation (*R*^2^ = 0.405) between increased chromatin accessibility and increased gene expression of the same gene (Figure 3C). Seventy-one differentially accessible chromatin regions aligned to 37 of 117 unique DEGs (20.9%) in the fibroblast RNA-seq data set (Supplemental Table 20). Where a DEG was represented by more than 1 significantly differentially accessible peak, we confirmed that the peaks were concordant in direction of change. A subset of these peaks mapped to the promoter-TSS (transcriptional start site) region, which usually indicates stronger effects on transcription. Fourteen of 71 (19.7%) peaks mapped to promoter-TSS regions, including the differentially accessible peak for *VANGL2*. This corresponded to 13 of 37 (35.1%) unique DEGs in the fibroblast RNA-seq data (Supplemental Figure 10). These peaks showed a clear association between increased promoter accessibility and increased transcription.

A subset of the DEGs identified through ATAC-seq translated across tissue types and were also found to be dysregulated in RNA-seq of BOS fibroblast and blood samples (Table 1). We identified 7 genes that were significant and differentially expressed across -omics levels (ATAC-seq and RNA-seq) and across tissues (blood and fibroblasts): *VANGL2*, *PRXL2A*, *APBA2*, *OTUD7A*, *KCNQ5*, *PELI2*, and *GREM2* (Supplemental Figures 11–13). These genes play significant roles in body patterning, neuron function, neural plate development, and ubiquitination, among other functions.

### Truncating ASXL1 mutations dysregulate DNAm, and contribute to changes in gene expression.

Endogenous CpG methylation levels at promoter regions are negatively correlated with gene expression (Figure 1D) (58). Thus, increased DNA CpG methylation or more closed chromatin is correlated with lower gene expression. To generate a BOS-specific DNAm signature, we profiled genome-wide DNAm in blood from individuals with BOS (*n* = 13) compared to sex- and age-matched control individuals (*n* = 26) (Supplemental Table 2) (51). DNAm identified 8596 differentially methylated CpG sites (FDR < 0.05) associated with an ENSEMBL gene, with 5773 CpG sites overlapping genes and mapping to 3803 unique genes. Seven hundred sixty-three differentially methylated CpG sites met a threshold of abs(Δβ) greater than 0.10 (10% DNAm difference) using linear regression modeling (Supplemental Table 21). These 763 differentially methylated CpG sites mapped to 163 unique genes, with 71 of 163 genes (43.6%) represented by more than one differentially methylated CpG site. After further filtering for effect size at a threshold of abs(log_2_FC) greater than 0.58, we found 24 unique genes remaining, represented by 50 differentially methylated CpG sites, including proteasome 20S subunit α8 (*PSMA8*) represented by 8 sites, Ellis van Creveld (*EVC*) represented by 6 sites, and *LRP5* represented by 3 sites (Supplemental Table 22).

**Figure 4:**
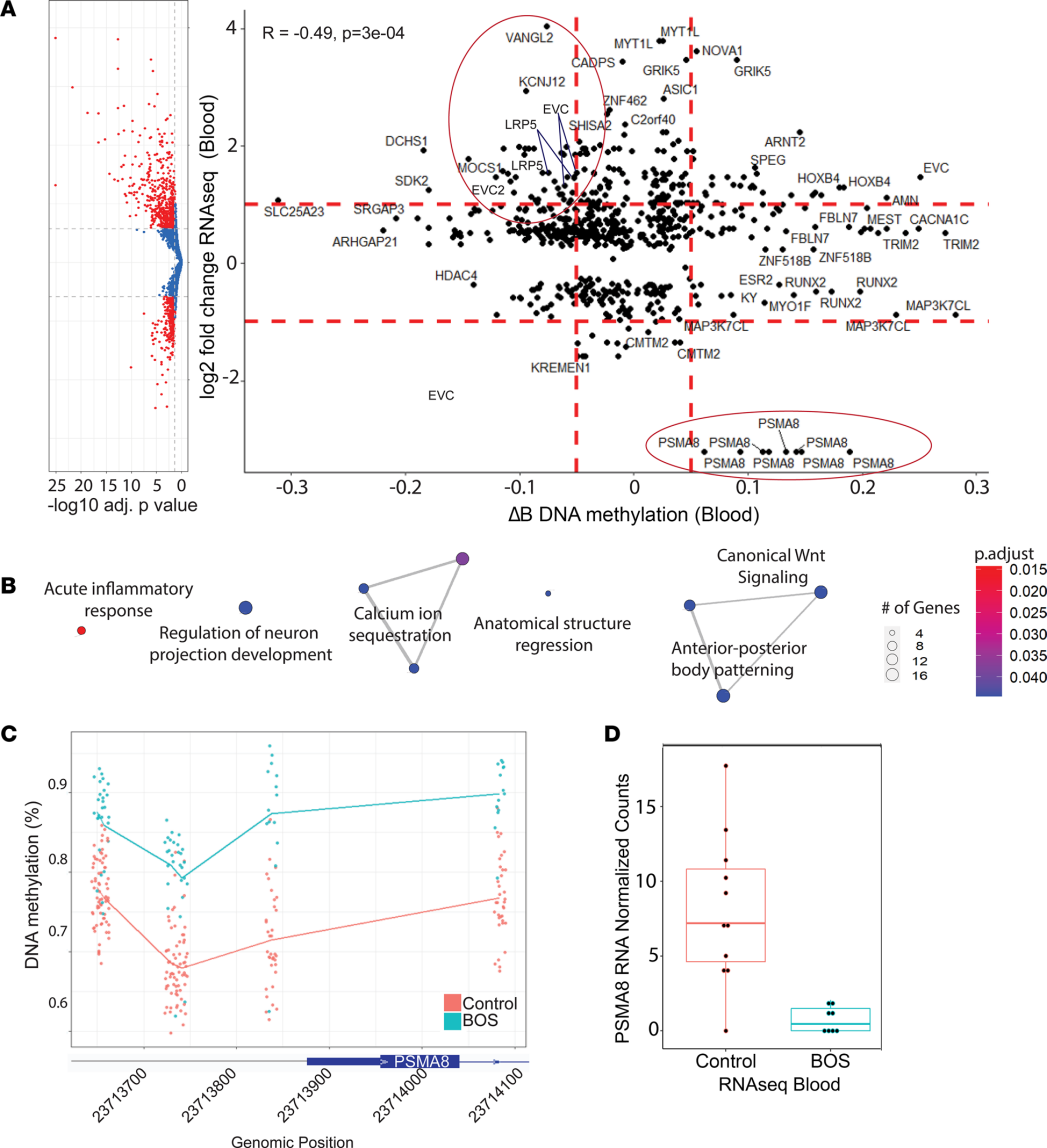
DNA methylation drives transcriptomic dysregulation in BOS samples and identifies common dysregulated transcripts enriched in Wnt signaling genes. (**A**) Integration of patient blood samples across DNAm (BOS *n* = 13, control *n* = 26) and RNA transcriptomic (BOS *n* = 8, control *n* = 11) dysregulation identified 672 differentially methylated CpG sites (*P*_adj_ < 0.05) that correlated to 341 RNA-seq DEGs (*P*_adj_ < 0.05). These significant DEGs were further filtered for RNA-seq abs(log_2_FC) ≥ 0.58, and DNAm abs(Δβ) ≥ 0.05, shown by the dotted red lines. After filtering, we retained 50 of 672 CpG sites (7.44%) and 24 of 341 unique genes (7.04%). (**B**) Analysis of enriched biological processes identified canonical Wnt signaling, anterior-posterior body patterning, regulation of neuron projection development, and other biologically relevant pathways. (**C**) In BOS patients, *PSMA8*, which encodes a key component of the β-catenin destruction complex, is hypermethylated in blood DNAm across 8 CpG sites (Δβ 6.1% to 18.9%) and (**D**) shows strong downregulation in blood RNA-seq (log_2_FC = –2.92). Control sample–normalized counts ranged from 0 to 17.7 (whiskers), with a mean (horizontal line) of 8.1, and quartile bounds (box limits) of 4.6 and 10.8. BOS normalized sample counts ranged from 0 to 2.1, with a mean of 0.8, and quartile bounds of 0 and 1.5.

To examine the likelihood of statistically significant differential methylation resulting in downstream transcriptional effects, we correlated BOS blood DNAm and RNA-seq expression profiles. Endogenous CpG methylation levels at promoter regions are negatively correlated with gene expression (58) (Figure 1D). We first identified differentially methylated CpG sites (FDR < 0.05) and DEGs from RNA-seq (*P*_adj_ < 0.05) separately. We found 672 differential CpG sites that mapped to 341 unique DEGs in the blood RNA-seq data set, with 143 genes represented by more than 1 differential CpG site (Supplemental Table 23). Genes represented by more than 1 CpG site were analyzed for concordance across the same gene and analyzed for their correlation with gene expression values for each individual sample. We filtered for highly differentially methylated sites (abs[Δβ] > 5%) and highly differentially expressed transcripts (abs[log_2_FC] > 1.5). After filtering, we retained 50 of 672 CpG sites (7.44%), which corresponded to 24 of 341 unique genes (7.04%) (Figure 4A). Eleven genes were represented by more than 1 CpG site, including *PSMA8* with 8 CpG sites and *GRIK5* with 2 CpG sites. *VANGL2* was represented by 1 CpG site. The correlation between these DNAm differences and transcriptomic changes indicates a steady-state effect of dysregulated DNAm on gene expression.

Three differentially methylated CpG sites, within the TSS, were identified for *EVC* and *EVC2*. *EVC* and *EVC2* were both hypomethylated at the TSS (–5% and –3.5%, respectively) and transcriptionally upregulated (log_2_FC = 1.50 and 1.54, respectively) in BOS patient blood.

We also generated BOS DNAm data using BOS (*n* = 8) and control fibroblasts (*n* = 8). DNAm identified 444 CpG sites at a nominal *P* value of 0.005 and abs(Δβ) greater than 0.10 (Supplemental Table 24). These CpG sites did not pass the FDR cutoff of 0.05 and so were not considered significant. Compared with blood, the fibroblast DNAm analysis showed significantly lower significance and effect level, which is consistent with our RNA-seq findings between blood and fibroblasts. This is likely a result of the homogeneity that is inherent to fibroblast cell culture compared with the heterogeneity of primary tissue, particularly blood. This, in conjunction with the reduced number of DEGs already identified in fibroblast RNA-seq compared with blood RNA-seq, likely resulted in no significant correlations between fibroblast DNAm and RNA-seq.

### Gene set enrichment analysis of DNAm and RNA-seq identifies activation of canonical Wnt pathway signaling in BOS samples.

The overlapping findings from the DNAm and RNA-seq data sets prompted us to run GO analysis for common gene targets, using 2 complementary methods, GREAT (60) and clusterProfiler v3.12.0 (61). We filtered for GOs with *P*_adj_ less than 0.05 for significance. In both analyses, significant gene set enrichments were identified in the Wnt signaling pathway (GO: 0060070, 17 genes; *P*_adj_ = 0.045), anterior-posterior pattern specification process (GO: 0007389, 19 genes; *P*_adj_ = 0.045), and regulation of neuron projection development (GO: 0010975, 19 genes; *P*_adj_ = 0.045), among other biologically relevant pathways (Figure 4B and Supplemental Table 25). Key genes that are enriched in these pathways include *PSMA8*, *WNT7A*, *FZD3*, *LRP5*, and *LRP6* in canonical Wnt signaling and body pattern specification. The latter pathway enrichment was also driven by strong dysregulation in *VANGL2*. These overlapping gene targets and pathways between methylome and transcriptome in patients with BOS suggest strong, coordinated, epigenetically driven effects of truncating *ASXL1* mutations.

*PSMA8* is a key driver gene in the canonical Wnt signaling enrichment identified in blood DNAm and RNA-seq data. We identified significant hypermethylation of *PSMA8* across all 8 CpG sites identified across the promoter and TSS region, with Δβ from 6.1% to 18.9% (Figure 4C, Supplemental Figure 14, A–F). *PSMA8* was also downregulated at the transcriptional level in BOS patient blood (log_2_FC = –2.92; Figure 4D). *PSMA8* was not expressed in BOS (*n* = 7) or control (*n* = 7) fibroblasts, with normalized read counts of 0 in all samples, and a read count of 1 in 1 BOS fibroblast sample (Supplemental Figure 14G).

### Truncating ASXL1 mutations disrupt canonical and noncanonical Wnt signaling.

**Figure 5:**
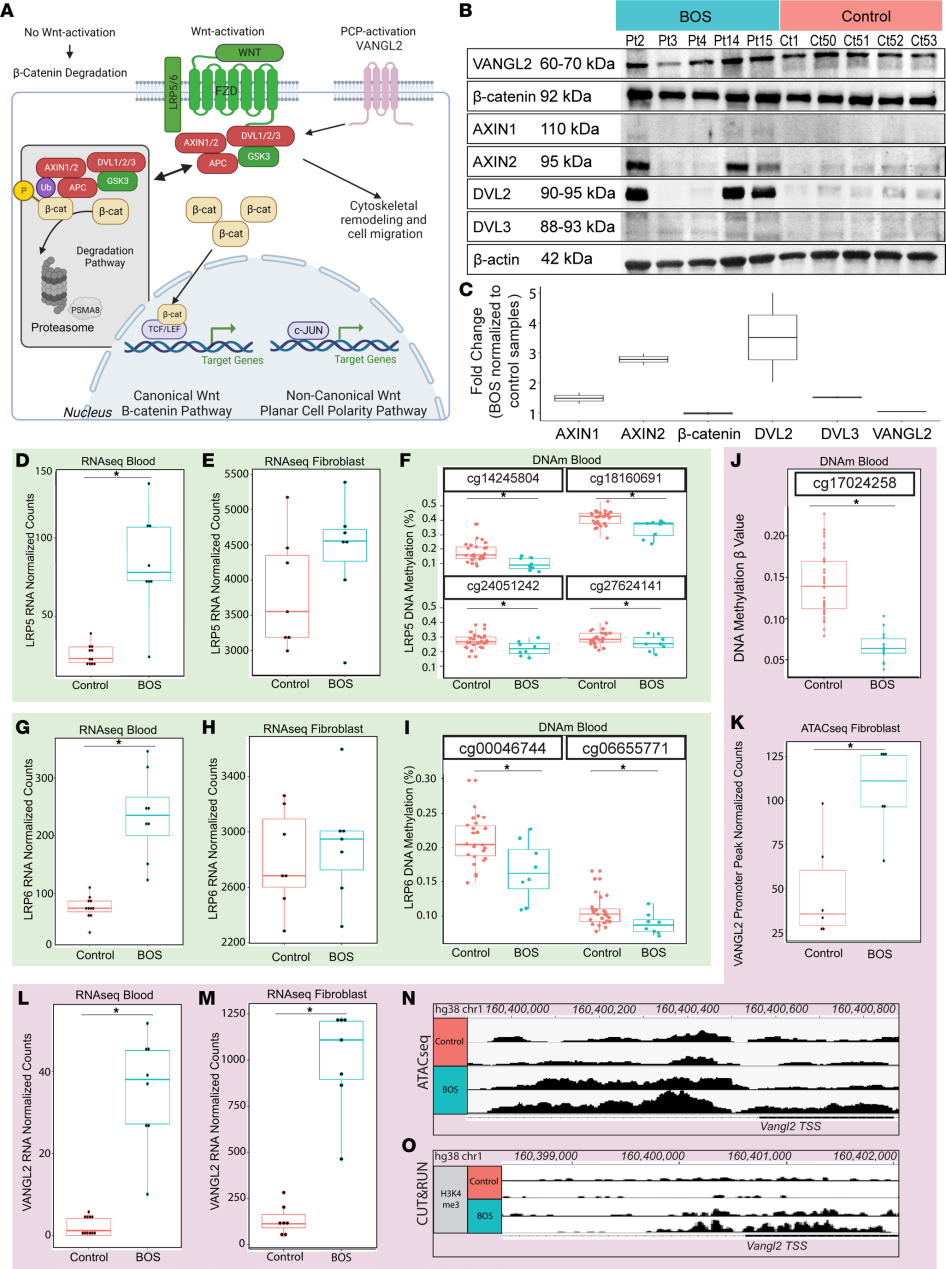
Truncated ASXL1 dysregulates the canonical and noncanonical Wnt signaling pathways. (**A**) The canonical Wnt signaling pathway (left) is activated when Wnt ligand stimulates its receptors. This inactivates the β-catenin destruction complex, allowing nuclear translocation of β-catenin and activation of target genes. Van Gogh–like 2 (VANGL2) intersects with the canonical pathway through activation of Dishevelled (DVL) to activate noncanonical pathways (right) and cell migration. (**B**) Whole-cell lysate (15 μg) of representative BOS- (*n* = 5) and control-derived (*n* = 5) fibroblasts show downstream Wnt pathway activation at the protein level through staining for VANGL2, β-catenin, axis-inhibition protein 1 (AXIN1), AXIN2, DVL2, and DVL3. (**C**) ImageJ (NIH) quantification identified an increase of 1.5-fold for AXIN1, 2.8-fold for AXIN2, 3.5-fold for DVL2, and 1.5-fold for DVL3 averaged across BOS patient samples compared to controls. This was repeated 2 times. In BOS patient samples, the Wnt pathway coreceptor *LRP5* (green) transcriptional upregulation in (**D**) blood (log_2_FC = 1.64, *P*_adj_ = 3.58 × 10^–9^) and (**E**) fibroblast RNA-seq and (**F**) DNA hypomethylation in blood (Δβ –3.5% to –8.0%, FDR < 0.05) at multiple CpG sites. Similarly, *LRP6* (green) shows that transcriptional upregulation in (**G**) blood RNA-seq (log_2_FC = 1.63, *P*_adj_ = 1.17 × 10^–12^), (**H**) fibroblast RNA-seq, and (**I**) DNA hypomethylation in blood (Δβ –2.7% to –4.0%, FDR < 0.05) BOS samples exhibit strong dysregulation of *VANGL2* across tissue and assay types (pink). For BOS samples, *VANGL2* is (**J**) hypomethylated (Δβ –7.6%) at CpG site cg17024258, and shows (**K**) increased chromatin accessibility at the 5′ UTR (log_2_FC = 1.20). *VANGL2* has significant transcriptional upregulation in (**L**) blood RNA-seq (log_2_FC = 3.80) and (**M**) fibroblast RNA-seq (log_2_FC = 2.55). In representative BOS patient samples, the *VANGL2* promoter shows (**N**) increased chromatin accessibility and (**O**) increased H3K4me3 marks compared with control. The box-and-whisker plots in **D**–**I**, **L**, and **M** show the mean (horizontal line), range (whiskers), and IQR [upper and lower box boundaries]. *LRP5* RNA-seq blood: control, 25.6 (18.3–39.1) [20.7–30.4]; BOS, 84.2 (24.1–134.6) [72.8–106.8]. *LRP5* RNA-seq fibroblast: control, 3829 (2996–5177) [3187–4351]; BOS, 4392 (2824–5390) [4268–4719]. *LRP6* RNA-seq blood: control, 75.0 (32.2–110.1) [67.8–86.6]; BOS, 234.5 (123.1–346.3) [199.9–266.9]. *LRP6* RNA-seq fibroblast: control, 2804 (2286–3262) [2602–3094]; BOS, 2904 (2381–3596) [2725–3006]. *VANGL2* RNA-seq blood: control: 2.3 (0–5.7) [0–4.1]; BOS, 35.3 (10.1–51.8) [27.2–45.2]. *VANGL2* RNA-seq fibroblast: control, 134.8 (39.4–282.8) [90.8–163.4]; BOS, 1002.7 (464–1235.6) [894.7–1210.1].

Through integration of these data, we identified dysregulation of the canonical Wnt pathway (Figure 5A) in gene set enrichment analysis of RNA-seq (Figure 2E) and DNAm studies (Figure 4B), driven by a consistent pattern of canonical Wnt/β-catenin pathway upregulation in the RNA-seq DEGs (Supplemental Table 26). Briefly, we identified upregulation of Wnt ligands *WNT1* (log_2_FC = 0.87), *WNT7A* (log_2_FC = 0.97), and *WNT10B* (log_2_FC = 0.53), and upregulation of the Wnt ligand receptor *FZD3* (log_2_FC = 0.87).

Western blot quantification of representative BOS fibroblasts (*n* = 5) and control (*n* = 5) fibroblasts identified no significant differences in 2 key proteins that are typically regulated through spatial distribution, VANGL2 and β-catenin, using bulk whole-cell lysate (Figure 5, B and C, and Supplemental Figure 16; see complete unedited blots in the supplemental material). Using a commercial anti-Wnt antibody kit, we identified an increase of 1.5-fold for AXIN1, 2.8-fold for AXIN2, 3.5-fold for DVL2, and 1.5-fold for DVL3 averaged across BOS patient samples compared with controls (Figure 5C). While the ASXL1, histone marks, VANGL2, β-catenin, and β-actin levels were consistent between BOS samples, Pt3 and Pt4 exhibited control-like levels of AXIN2, DVL2 and, to some extent, DVL3.

RNA-seq and DNAm studies identified aberrant upregulation and hypomethylation of Wnt signaling coreceptor genes *LRP5* (Figure 5, D–F) and *LRP6* (Figure 5, G–I). We identified significant transcriptional upregulation of *LRP5* (log_2_FC = 1.64, *P*_adj_ = 3.58 × 10^–9^; Figure 5, D and E) and *LRP6* (log_2_FC = 1.63, *P*_adj_ = 1.17 × 10^–12^; Figure 5, G and H), 4 hypomethylated DNAm sites for *LRP5* (Δβ –3.5% to –8.0%, FDR < 0.05; Figure 5F), 2 of which reside in a CpG island, and 2 hypomethylated DNAm sites for *LRP6* (Δβ –2.7% to –4.0%, FDR < 0.05), both of which reside in a CpG island (Figure 5I).

We also identified transcriptional upregulation of *AXIN2* (log_2_FC = 0.95) and downregulation of *GSK3B* (log_2_FC = –0.28), consistent with canonical Wnt signaling activation. Endogenous *AXIN2* mRNA and protein expression is directly induced by Wnt pathway activation (62, 63) and used as a proxy reporter for Wnt pathway signaling. Furthermore, in BOS patient samples, the Wnt pathway transcription factors *TCF7* (log_2_FC = 0.81) and *LEF1* (log_2_FC = 0.86) were both significantly transcriptionally upregulated.

### Multiomics identifies altered epigenetic profile in VANGL2, a noncanonical Wnt signaling gene, in BOS.

*VANGL2* first came to our attention as one of the most highly overexpressed DEGs in BOS blood and fibroblast RNA-seq data (Figure 2G and Supplemental Figure 15, A and B). Integration of RNA-seq, ATAC-seq, and DNAm data again identified *VANGL2* as differentially regulated in all data sets (Figure 3C and Figure 4A). In BOS blood samples, we observed *VANGL2* transcriptional upregulation coupled with hypomethylation (Δβ = –7.6%) at the TSS of *VANGL2* CpG site cg17024258 (Figure 5J) and an inverse relationship between hypermethylation and transcriptional upregulation in individual samples (Supplemental Figure 15C). The 5′ UTR of *VANGL2* showed a 2.3-fold increase in chromatin accessibility by ATAC-seq (Figure 5K).

These findings were supported by differential *VANGL2* gene expression in BOS patients versus control samples. Cross-tissue RNA-seq integration (Figure 2G) showed that *VANGL2* was significantly overexpressed in both BOS patient blood (log_2_FC = 3.8; Figure 5L) and in fibroblasts (log_2_FC = 2.55; Figure 5M).

Visualization of ATAC-seq data in IGV identified a clear increase in reads for BOS patients at the *VANGL2* TSS, representing more open chromatin (Figure 5N). Similarly, CUT&RUN for H3K4me3, a transcription-activating histone mark, showed significantly stronger H3K4me3 binding at the *VANGL2* TSS for BOS compared with controls (Figure 5O). *ASXL1* has been previously shown to affect histone modifications, specifically H3K4me3, H3K27me3, and H2AK119ub, and our data further corroborate that truncating mutations in *ASXL1* result in loss of its gene repressive functions.

## Discussion

We utilized a multiomics approach to profile primary BOS patient samples. For the first time to our knowledge, we directly link *ASXL1* mutations to activation of the canonical and the noncanonical Wnt signaling pathway, namely the PCP pathway. Both pathways are essential in developmental patterning. The PCP pathway defines cell polarity and is essential to morphogenesis and diverse cellular processes (64). The data demonstrate that upregulation of Wnt pathways in BOS samples can be attributed to disruption of the epigenetic landscape, ultimately resulting in transcriptomic dysregulation.

Most of the studies on human ASXL1 function are in the context of myeloid leukemia and propose that truncating *ASXL1* mutations result in haploinsufficiency (11, 36), dominant-negative, or gain-of-function effects (31, 37, 38). In some reports, in vivo deletion of *Asxl1* led to mild phenotypes of myeloid and lymphoid cell frequencies (7), while in other reports, *Asxl1*-knockout mice had systemic developmental defects and MDS-like presentation, suggesting a loss-of-function effect particularly in hematopoietic progenitor cells (11, 36). However, most *ASXL1* mutations identified in human myeloid malignancies and in BOS do not harbor a deletion and, instead, harbor C-terminal nonsense or frameshift mutations. These mutations are hypothesized to escape nonsense-mediated decay and may potentially have a dominant-negative or gain-of-function effect (31). Our findings of comparable ASXL1 transcript and protein expression between BOS and control samples (Figure 1D, Figure 2, A and B, and Supplemental Figure 2) are consistent with this hypothesis and show no evidence of degradation or nonsense-mediated decay. The dysregulation observed in BOS may be a result of the combination of haploinsufficiency of the full-length transcript and translation of a short and nonfunctional protein that results in mistargeting of ASXL1 functions. Furthermore, developmental context may also play a role. This hypothesis is supported by induced pluripotent stem cell (iPSC) studies conducted by Matheus et al. (9).

We also found comparable levels of wild-type and pathogenic transcripts in BOS patients. This adds further evidence to existing literature showing that nonsense-mediated decay and haploinsufficiency do not play a role in BOS pathogenesis (37, 65), which is instead mediated through misexpression and impact, potentially through gain of function, of a truncated ASXL1 protein.

### High-penetrance mutations in severe monogenic disorders supersede effects of age, sex, and genetic background in molecular assays.

Although there is strong tissue-specific gene expression (66), we propose that the high-penetrance effect of truncating *ASXL1* mutations results in a set of dysregulated genes across tissues. The 25 common DEGs across BOS blood and fibroblast data support the overarching hypothesis that truncating *ASXL1* mutations exert a strong tissue-independent effect across tissues that have *ASXL1* expression. The functions of these key genes likely drive BOS pathophysiology and targeting these genes may provide a systemic treatment for BOS.

Some key challenges to the study of rare disease are the heterogeneity of ages at sample collection and diverse genetic background of patients. Controlling for age-specific effects and genetic background are important but would require statistical power that is nearly impossible with fewer than 100 patients reported worldwide. The control individuals for DNAm were matched for age- and sex-specific effects, while ATAC-seq and RNA-seq controls were matched for sex and genetic background effects. Integration between ATAC-seq, RNA-seq, and DNAm data, and the identification of common dysregulated genes suggests that the role of truncating *ASXL1* mutations supersedes the effects of age, sex, and genetic background.

Integration of ATAC-seq and RNA-seq identified 7 genes that were commonly dysregulated (Table 1). We tested these genes specifically for age-specific effects since that was a variable we were unable to sufficiently match, given the young age of the BOS patient cohort and restricted access to samples from healthy young children who do not require procedures. In our ATAC-seq and RNA-seq assays, we primarily used related parental controls to account for genetic variation in our population. The 7 genes that were commonly dysregulated in our ATAC-seq and RNA-seq integration (Table 1) were all tested as part of a larger study by Lee et al. (67) through transcriptome analysis to classify human genes based on age-specific differential expression analysis. None of these genes showed an age-specific signature. These 7 genes have important functions in body patterning and organogenesis, nervous system development, and ubiquitination regulation. While this does not rule out age-specific differences in potential interacting proteins, it suggests that the effects of truncating *ASXL1* mutations are not significantly impacted by age.

### ASXL1 represses canonical and noncanonical Wnt signaling in normal development.

One of the major findings in this study is the linking of *ASXL1* mutations and the activation of the canonical and noncanonical Wnt signaling pathways. The canonical Wnt signaling pathway plays an important role in embryonic development, stem cell maintenance, and differentiation of cells in adults and is highly conserved across species (68). The Wnt signaling pathway is under complex regulation and functions in context-specific manners, i.e., dependent on the receptors present on the cell membrane and Wnt ligand–receptor interactions at the time of pathway activation (69).

The molecular mechanisms and specific developmental stages of the interactions between the canonical and noncanonical Wnt pathways are not well elucidated. In the absence of Wnt signaling, β-catenin is degraded by the 26S proteasome, which is composed of 2 subcomplexes, the 20S proteasome and the regulatory particle (70, 71) (Figure 5A). PSMA8 is a subunit of the 20S proteasome, and hypermethylation and transcriptional downregulation of *PSMA8* in BOS patient samples (Figure 4, C and D) suggests downregulation of the 26S proteasome–mediated degradation of β-catenin in BOS patient tissues (70). While PSMA8 is involved in numerous pathways within the cell, given the context of the many other Wnt signaling pathway genes that were identified as differentially regulated and differentially expressed (Supplemental Table 26), the hypermethylation and transcriptional downregulation of PSMA8 supports our findings of aberrant Wnt activation in RNA-seq (Figure 2E) and DNAm studies (Figure 4B) of BOS.

The β-catenin destruction complex consists of AXIN1, AXIN2, DVL1–3, GSK3B, and adenomatous polyposis coli (APC). We identified increased protein levels of AXIN1, AXIN2, DVL2, and DVL3 in BOS patient fibroblast samples. Pt3 and Pt4 exhibited control-like levels of AXIN2, DVL2, and, to some extent, DVL3. These 2 patients have truncating mutations within close proximity, at amino acid 635 and 642, respectively. This may point to a location-specific effect of ASXL1 mutations, although more work is needed to elucidate this. BOS is a heterogeneous disorder and patient-specific factors such as sex and age, or environmental effects may result in differential gene regulation. Pt3 and Pt4 are male BOS patients, while Pt2, Pt14, and Pt15 who are female BOS patients have much higher levels of Wnt pathway proteins. No significant difference was identified between male and female control samples. Thus, this may point to sex-linked differential effects of truncating ASXL1 mutations on Wnt pathway protein expression, and would benefit from further study and replication in increased numbers of patient samples.

Western blotting did not identify a difference in total whole-cell β-catenin or VANGL2 levels between BOS and control fibroblasts (Figure 5, B and C; see complete unedited blots in the supplemental material). The genes encoding these 2 proteins are both posttranslationally regulated, which may ultimately drive phenotype effects. More work is needed to elucidate the role of cellular localization of canonical and noncanonical Wnt proteins, and the role they play in BOS pathogenesis.

β-Catenin localization is important in identifying activation of the Wnt signaling pathway. In the Wnt-active state, the β-catenin destruction complex is dissociated and culminates in nuclear translocation of β-catenin (43–45) (Figure 5A). This nuclear β-catenin binds to the TCF and LEF families, and initiates transcription of Wnt pathway targets (72, 73). *TCF7*, which is transcriptionally upregulated in BOS samples, is an important regulator of self-renewal and differentiation in multipotential hematopoietic cell lines (74), which could offer insight into the pathogenesis of myeloid cancers with truncating *ASXL1* mutations.

In contrast, the noncanonical pathways, including the PCP pathway that defines cell polarity and migration, are characterized by their β-catenin–independent regulation of crucial events during embryonic development (75). However, experiments that indicate their binary utilization may be model-system and context specific (48). Given the complex interplay and cross-talk at almost every level of the Wnt signaling pathways that we are just beginning to unravel, van Amerongen and Nusse suggest moving toward a more integrated view rather than the outdated binary classification (48).

### Dysregulation of the Wnt signaling pathway identified in BOS samples may explain phenotypic presentations in BOS patients.

Wnt signaling plays a key role in hair growth and development of hair follicles. In particular, *WNT10B*, which is transcriptionally upregulated in BOS samples, promotes differentiation of primary skin epithelial cells toward the hair shaft in mice and elongation of the hair shaft in isolated rabbit whisker hair follicles (76–78). Intriguingly, many healthcare providers and parents have noted excessive hypertrichosis with rapidly growing hair and nails in BOS patients (25). It is possible that aberrant activation of Wnt signaling may play an important role here in BOS patient pathophysiology.

Other significant Wnt signaling DEGs in our data sets include *WNT1*, important in regulating cell fate and patterning during embryogenesis, and *WNT7A*, a key embryonic dorsal versus ventral patterning gene and a key regulator of normal neural stem cell renewal, proliferation, and differentiation. WNT7A activates the PCP pathway and *Wnt7a* overexpression has been shown to induce *Vangl2* overexpression and impair neurulation in mouse neural stem cells through aberrant VANGL2 polarized distribution (79, 80).

Collectively, our epigenomic, transcriptional, and protein data of the Wnt signaling pathways corroborate a distinct and consistent activation of canonical Wnt signaling in BOS samples and may offer insight into patient phenotypes.

### Upregulation of noncanonical Wnt signaling gene VANGL2 in BOS drives clinical phenotypes.

*VANGL2* was one of the most highly dysregulated genes in all our data sets, with a 9-fold increase in gene expression occurring via epigenetic dysregulation (Figures 5, J–O). VANGL2 is thought to regulate Wnt protein distribution (81) and, in its noncanonical role, has been shown to play critical roles in neurulation, cardiac development, kidney-branching morphogenesis, and regulation of hematopoiesis (49, 82–84). The PCP pathway is a highly conserved noncanonical Wnt signaling pathway important in establishing and maintaining polarity during morphogenesis. While we identified no significant difference in total VANGL2 protein at the whole-cell level between BOS and control fibroblasts (Figure 5, B and C), like β-catenin, VANGL2 function is strongly dependent on cellular localization. The asymmetric localization of VANGL2 on the plasma membrane is needed for signal transduction and subsequent polarization and organization of cells (85). The PCP pathway is also crucial for neural tube closure (86) and it has been suggested that the gradient of Wnt activity helps establish VANGL2 polarity in the neural plate during neurulation (87), with the canonical Wnt signaling required for neural crest induction and the noncanonical Wnt pathway required for neural crest migration (88).

Knockout and loss-of-function studies of *Vangl2* identified significant reduction in spine density and dendritic branching in primary-culture rat hippocampal neurons (49). In humans, homozygous mutations in *VANGL2* are embryonic lethal and cause craniorachischisis, a very severe neural tube defect encompassing anencephaly and bony defects of the spine in mice (89), whereas heterozygous *VANGL2* mutations are embryonic lethal and detected in miscarried human fetuses with severe cranial neural-tube defects (90). Neural crest cells contribute to nervous system and craniofacial development, and are critical for cardiac outflow septation and alignment (91). Perhaps not surprisingly, *Vangl2*-knockout mice exhibit cardiac outflow tract malformations and septal defects (82), as do BOS patients (25). These findings suggest that the severe neural phenotype, distinctive craniofacial features, and cardiac defects of patients with BOS (25) may be due, at least in part, to the dysregulation of *VANGL2* and downstream effects on neural crest migration.

Although the interplay between the canonical Wnt and noncanonical Wnt pathways is not well understood, recent literature has suggested that these pathways exert reciprocal pathway inhibition through competition for the common downstream coreceptor FZD (75, 92), most evident during tissue regeneration and development (93, 94). The asymmetric localization of FZD3 required for proper function is dependent on anchoring by VANGL2 through physical interaction of the 2 proteins (95). We identified upregulation of FZD3, which regulates establishment of the noncanonical Wnt/PCP pathway and is involved in neural crest cell migration and neural tube closure (96, 97). These results are supported by molecular characterization of truncating *ASXL1* mutations conducted in iPSCs (9).

A limitation of this study is that BOS pathophysiology is shaped by cellular context and developmental stage and our study is focused on differentiated cell types. Cellular and developmental context can drastically affect the molecular effects of epigenetic modifiers such as ASXL1. The effects of ASXL1 begin in early embryogenesis (98), so embryonic stem cells would be the ideal model to recapture cell types where ASXL1 is maximally impacting progenitors of clinical phenotypes. These multiomics approaches will benefit from being performed in patient-derived stem cell models and through samples generated with introduction of truncating *ASXL1* mutations using DNA editing systems. Moving into stem cell models will yield additional insights into the role of ASXL1 in stem cell homeostasis and differentiation.

In conclusion, we present a comprehensive multiomics analysis of BOS using primary patient samples. This study encompasses the largest -omics study of patients with BOS, a very rare genetic disorder. We present the first analyses to our knowledge of RNA-seq and ATAC-seq conducted on primary BOS patient samples. Through DNAm analysis, identifying binding proteins, deciphering chromatin accessibility, and gene expression analysis, we add to growing literature on ASXL1 and the pathogenesis of *ASXL1* truncating mutations. Our integrated methods identified dysregulation of key transcripts such as *VANGL2* through direct chromatin modifications. This suggests that physiological levels of truncating *ASXL1* mutation lead to loss of the repressive function of ASXL1. Importantly, pathways that were dysregulated across multiple assays are involved in neural development, which shed light on the severe intellectual and neural features in BOS patients, and Wnt/β-catenin and noncanonical Wnt PCP pathways that represent key, drug-targetable pathways. Our findings have major implications for identifying potential therapeutics for diseases harboring *ASXL1* mutations.

## Methods

### Cell culture.

Patient-derived fibroblast cell lines were grown in DMEM (Gibco, 11-995-073), 10% FBS, 1% nonessential amino acids (100×, Gibco, 11140-050), and 1% penicillin/streptomycin at 37°C in 5% CO_2_ incubators.

### Western blotting.

Whole-cell lysates were extracted with Cell Lysis Buffer (10×, Cell Signaling Technology, 9803S), cytoplasm and nuclei extracted using the CE/NER Kit (Thermo Fisher Scientific, 78833), and histones extracted as per established protocol (99). See Supplemental Methods.

### RNA-seq.

Fibroblasts were grown to 80%–90% confluence in T75 flasks before total RNA extraction via RNeasy Plus Mini Kit (Qiagen, 74136). Blood samples were first treated with QIAseq FastSelect −Globin (Qiagen, 334376). All samples were then prepared with QIAseq FastSelect −rRNA HMR (Qiagen, 334386) and TruSeq Stranded Total RNA Library Prep Gold (Illumina, 20020599), before sequencing to a minimum of 30 million paired-end 150-bp reads per sample. See Supplemental Methods. RNA-seq data have been deposited in the NCBI Gene Expression Omnibus database (GEO GSE230696).

### ATAC-seq.

Fibroblast lines were cultured to 80%–90% confluence in T75 flasks and 50,000 freshly isolated cells per line were treated with Tn5 transposase, as per established protocol (100). Libraries were sequenced to a minimum of 40 million paired-end reads per sample, with 75-bp length. See Supplemental Methods.

### CUT&RUN.

CUT&RUN libraries were prepared with the established CUT&RUN Assay Kit (Cell Signaling Technology, 86652S). See Supplemental Methods. Samples were analyzed using the established NF-Core CUT&RUN analysis pipeline (101).

### DNAm.

See Supplemental Methods.

### RT-qPCR.

RT-qPCR was conducted using TaqPath 1-Step RT-qPCR Master Mix, CG (Applied Biosystems, A15300) according to the manufacturer’s protocol (document 100020171, rev 1.00) using a QuantStudio 3 (Thermo Fisher Scientific). See Supplemental Methods for further information.

### Statistics.

RNA-seq, ATAC-seq, and CUT&RUN reads were aligned to the human genome (hg38) and featureCounts (v1.6.5) (see Supplemental Methods) was used to generate count matrices for genes or chromatin regions. DESeq2 (v1.24.0), which internally normalizes sample library size and utilizes the negative binomial distribution for testing, was used to identify DEGs (RNA-seq) or differentially accessible chromatin regions (ATAC-seq, CUT&RUN) after adjusting for sample sex, which was identified as a covariate to adjust for through PCAs generated based on the top 500 most variably expressed genes. Genes or regions were identified as significant when Benjamini-Hochberg–adjusted *P* values (*P*_adj_) were less than 0.05. We further filtered for highly dysregulated significant genes or regions using an abs(log_2_FC) of 0.58 or greater, corresponding to an abs(FC) of 1.5 or greater with reference to control samples. GO overenrichment tests were completed using clusterProfiler v3.12.0 by submitting DEGs against all genes from the Gencode hg38 annotation, v31 (see Supplemental Methods). GOs were classified as significantly enriched when *P*_adj_ (Benjamini-Hochberg) was less than 0.05 (hypergeometric test).

DNAm β values were calculated using minfi (102), and differentially methylated sites were identified by running Limma regression modeling, while adjusting for the covariates of age, sex, and ethnicity. Our significance cutoff was an FDR of less than 0.05 (Benjamini-Hochberg). We filtered for significantly methylated CpG sites with abs(Δβ) greater than 0.10, where Δβ represents the difference in average DNAm (β) between BOS and controls. GREAT (60) was run on significant CpG sites to identify biological mechanisms that were dysregulated, filtering for GOs with *P*_adj_ less than 0.05 for significance.

### Study approval.

This project was approved by the UCLA IRB protocol 11-001087. In conjunction with Bianca Russell and the ASXL Biobank at UCLA, written informed consent from participants and guardians were obtained for skin punch biopsies and blood samples. Deep phenotyping and review of medical records was also collected.

## Author contributions

VAA, ZA, and IL designed and conceptualized the study and wrote the manuscript. IL, MS, and AW performed data generation and transcriptomic and epigenomic data analysis for patient-derived samples. BER and REACH coordinated sample collection. IL, AH, and AN performed Western blotting and quantification. ZA and RW performed data generation and analysis of DNAm data. All authors contributed to the writing and editing of the manuscript.

## Supplementary Material

Supplemental data

Supplemental tables 1-26

## Figures and Tables

**Table 1:**
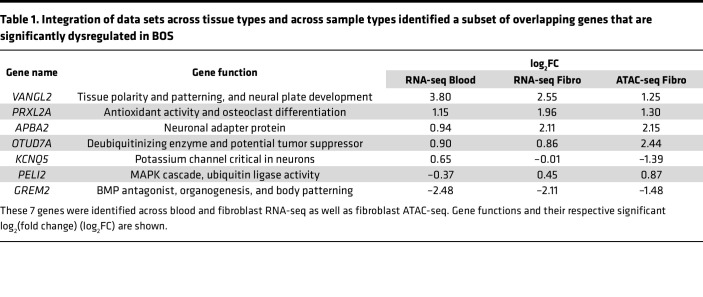
Integration of data sets across tissue types and across sample types identified a subset of overlapping genes that are significantly dysregulated in BOS

## Version 1 04/13/2023

In-Press Preview

## Version 2 05/22/2023

Electronic publication
