# Supplementary material for: Multiomics of Bohring-Opitz syndrome truncating ASXL1 mutations identify canonical and noncanonical Wnt signaling dysregulation
Source: JCI Insight. 2023 May 22;8(10):e167744. doi: 10.1172/jci.insight.167744 (PMC10322691; doi:10.1172/jci.insight.167744)
Supplement: Supplemental data [file jciinsight-8-167744-s106.pdf]

SUPPLEMENTAL METHODS

Sample Collection

All individual level-data was de-identified prior to analysis and samples were collected for follow-up experiments. We collected blood from 12 BOS individuals and 38 controls, and skin punch biopsies from 6 individuals and 183 controls. We used two independent samples (blood and patient-derived fibroblast) from 4/18 BOS individuals to filter out noise of genetic background. Whole blood was processed to isolate DNA, RNA and PBMCs and skin-punch biopsy samples were processed to create patient-derived skin fibroblasts in the Translational Pathology Core Laboratory at UCLA.

Cell Culture cont.

All cell lines were tested for mycoplasma (MycoAlert PLUS Mycoplasma Detection Kit, Lonza #LT07-318) on a monthly basis.

Transfections

For transfection experiments, HEK293T cells and CACO2 cells were grown in DMEM (Gibco™, #11-995-073), 10% FBS, and 1% PenStrep at 37℃ in 5% CO2 incubators. CACO2 cells were a gift from the Tontonoz lab at the University of California, Los Angeles.

Whole Cell Lysis Protein Extraction

Cells were harvested in ice-cold 1X cell lysis buffer (10X, Cell Signaling #9803S) containing 1X Halt™ Protease and Phosphatase Inhibitor Cocktail (100X, Thermo Scientific™ #PI78442). Soluble lysate fractions were isolated by constant agitation at 4℃ for 30 minutes at 1000 rpm and then centrifugation at 20,000 × *g*, for 20 min at 4 °C.

Cytoplasmic and Nuclear Extractions

1 million cells were harvested into a 1.5ml eppendorf tube and centrifuged at 500 ×g for 5 min to form a pellet. Cells were washed with ice cold PBS with 0.5mM Sodium Butyrate and centrifuged at 500 × g for 2 min to form a pellet. After centrifugation, the supernatant was discarded. Ice-cold CERI with 1X Halt™ Protease and Phosphatase Inhibitor Cocktail (100X, Thermo Scientific™

#PI78442) was added to each cell pellet. Cytoplasmic and nuclear extraction was conducted using the Thermo Scientific CE/NER Kit (#78833) according to protocol.

Histone Extraction

Histones were extracted as per established protocol (1).

Western Blot

Protein was quantified using the Pierce BCA Protein Assay Kit (Thermo Scientific #23225). For Western blots, we used 15ug or 30μg of whole cell lysate, 10μg of cytoplasmic extract, 3ug of nuclear extract or 1ug of acid-extracted histone per well for 30 minutes at 130V. Proteins were then transferred to 0.2uM nitrocellulose membranes (BioRad #1704271) using the semi-dry TransBlot for 7 min at 25 mA. The membranes were blocked with 5% milk in 1x TBST and then probed with primary antibodies (1:1000) overnight at 4℃. The antibodies for whole cell lysate, cytoplasmic and nuclear extracts were ASXL1 (abcam, #ab228009), DVL2 (30D2) (Cell Signaling, #3224), DVL3 (Cell Signaling, #3218), AXIN1 (C76H11) (Cell Signaling, #2087), AXIN2 (Thermofisher, #PA521093), VANGL2 (Proteintech, #21492-1-AP), β-Catenin (Invitrogen, #71-2700), and hFAB Rhodamine Anti β-Actin (Bio-Rad, #12004163). For antibodies used in supplemental figures, see Supplemental Table 6. The antibodies for histone extracts were histone H3 core (D1H2) (Cell Signaling, #4499), Tri-Methyl-Histone H3Lys4 (C42D8) (Cell Signaling, #9751), Tri-Methyl-Histone H3Lys27 (C36B11) (Cell Signaling, #9733), Ubiquityl-Histone H2ALys119 (D27C4) (Cell Signaling, #8240).

Membranes were then probed with secondary antibodies (1:10,000), at room temperature for one hour according to manufacturer dilutions. These secondary antibodies were Goat anti-Rabbit IgG Highly Cross-Adsorbed, Alexa Fluor™ 680 (Invitrogen #A-32734) and Goat anti-Mouse IgG Highly Cross-Adsorbed, Alexa Fluor™ 488 (Invitrogen #A-11001). Blots were imaged on a Biorad ChemiDoc. For a full list of antibodies, see Supplemental Table 6.

Blood Sample Collection and Processing.

Peripheral blood samples were collected into EDTA tubes for DNA extraction and PBMC extraction, CPT tubes for PBMC extraction and PAXgene™ Blood RNA Tube (BDBiosciences, 762165) for RNA extraction. Whole blood DNA was extracted using the Qiagen Blood and Cell Culture DNA Midi Kit (Qiagen #13343), and whole blood RNA was extracted using the MagMAX™ for Stabilized Blood Tubes RNA Isolation kit (Thermo Fisher Scientific, #4451893) with DNase treatment. All samples were quantified on the nanodrop, Qubit 4.0 and Tapestation Bioanalyzer for sample quality control. DNA and RNA were stored at -80C.

RNA-sequencing cont.

Fibroblast and blood samples were quantified on the Qubit 4.0 and Tapestation Bioanalyzer 4150 using High Sensitivity RNA Tapestation (Agilent, #5067-5579) to ensure high-quality RNA (RIN $\geq$ 9.0 for fibroblasts, RIN $\geq$ 5.3 for blood). All libraries were quantified on the Qubit 4.0 and library quality was assessed using Agilent D1000 DNA Tapestation (Agilent, #5067-5582) before multiplexing and sequencing on a NovaSeq flow cell for a minimum of 30 million paired-end 150bp reads per sample. Fastq files were processed through our best practice bioinformatic in-house pipeline (Supplemental Figure 19).

RNA-seq Bioinformatic Analysis. (Supplemental Figure 19)

Samples were demultiplexed after sequencing, and fastq sequencing files were processed using established pipelines in the Arboleda lab. Raw read quality, adaptor content, and duplication rates were assessed with *FastQC v0.11.8*.(2) Raw reads were then aligned against the Gencode human genome version hg38 (GR38) version 31 using STAR *2.7.0e* (3) with default parameters. Gene counts from raw reads were generated using featureCounts *1.6.5* (4) from the Subread package. Only reads that uniquely mapped to exons of a gene were counted per gene. Differential expression analysis was completed using DESeq2 *v1.24.0 (5),* adjusting for sample gender. Multidimensional scaling based on the top 500 genes with the highest variance was performed and verified that BOS samples clustered together and away from controls (Supplemental Figures

17A and 18A). We confirmed effective rRNA removal (Supplemental Figures 17B and 18B). Differential expression analysis was performed, adjusting for sample sex. Genes with a p-adjusted (Benjamini-Hochberg) less than 0.05 were classified as significantly differentially expressed (Wald's test), and fold changes (5) were shrunk using approximate posterior estimation for GLM coefficients. We further filtered our differentially expressed genes (DEGs) for abs(log2FC) ≥ 0.58, corresponding to an absolute log fold change ≥ 1.5. Gene ontology (6, 7) over-enrichment tests were completed using clusterProfiler *v3.12.0 (8)* by submitting differentially expressed genes against all genes from the Gencode hg38 annotation, version 31. Gene ontologies were classified as significantly enriched when p-adjusted (Benjamini-Hochberg) was less than 0.05 (hypergeometric test). *HOMER v4.9 findMotifs.pl (9)* was used to identify enrichment of motifs within differentially expressed genes' promoters. Differentially expressed genes were compared against HOMER's default human, RefSeq-based promoter set, which yielded de novo and known motif enrichments for motif lengths of 8, 10, and 12. Sequencing tracks were visualized using Integrated Genomic Viewer (IGV 2.9.4) and GVIZ.

ATACseq cont.

After purification and PCR amplification for library generation, the libraries were double-sided bead purified using AMPure XP beads (Beckman, A63881) to remove contaminating primer dimers. All libraries were quantified on the Qubit 4.0 and library quality was assessed using Agilent High Sensitivity DNA Tapestation (Agilent, 5067-5584) before multiplexing and sequencing on a Nextseq 550 for a minimum of 40 million paired-end reads per sample with 75bp length.

ATACseq Bioinformatic Analysis. (Supplemental Figure 20)

Fastq files were processed through our best practise bioinformatic in-house pipeline (Supplemental Figure 20). Quality of reads were assessed using FastQC. Raw reads were then aligned to GENCODE Human genome version hg38 (GR38) version 31 using BWA-MEM (10).

BAM files were then sorted, indexed, filtered against chrX, chrY, and MT reads using *SAMtools*. *Picard* tools were then used to generate insert size histograms and remove duplicates from BAM files. Narrow peaks from each sample were called using MACS2 callpeak (11); any peak that overlapped by at least one base was then merged using BEDtools merge (12) *merge*. Reads overlapping merged peaks were counted using featureCounts (4). Technical replicates clustered closely on PC analysis and were collapsed during analysis. Peaks were annotated using annotatePeaks.pl from HOMER to the nearest gene or regulatory element, including exon, intron, promoter, 5' untranslated region (UTR), 3' UTR, and other genomic features (9). *DESeq2* was used to identify differentially open peaks between disease and control samples, adjusting for sample gender. Peaks with p-adjusted value (Benjamini-Hochberg) less than 0.05 were classified as significantly differentially open (Wald's test), and fold changes were shrunk using approximate posterior estimation for GLM coefficients. Significant peaks were identified as promoter peaks if their distance from their respective closest gene was less than 1kb upstream or 2kb downstream relative to the gene transcription start site. Gene ontology over-enrichment tests were completed using clusterProfiler by submitting the closest genes to significantly differentially open peaks against all genes from the Gencode hg38 annotation, version 31. Gene ontologies were classified as significantly enriched when p-adjusted (Benjamini-Hochberg) was less than 0.05 (hypergeometric test). Enriched de novo and known motifs were identified in significant peaks using findMotifsGenome.pl from HOMER (9). Significant peaks were identified as promoter peaks if their distance from their respective closest gene was less than 1kb upstream or 2kb downstream relative to the gene transcription start site. Sequencing tracks were visualized using Integrated Genomic Viewer (IGV 2.9.4) and GVIZ.

CUT&RUN.

CUT&RUN libraries were prepared using the SimpleChIP ChIP-seq DNA Library Prep Kit for Illumina (Cell Signaling, #56795S) with SimpleChIP ChIP-seq Multiplex Oligos for Illumina (Cell Signaling, #47538S) according to manufacturer guidelines, with modifications for CUT&RUN as

noted in the CUT&RUN Assay Kit (Cell Signaling, #86652S). The following antibodies were used for CUT&RUN libraries: IgG isotype (DA1E) (Cell Signaling, #66362, negative control, 1:20), H3K4me3 (C42D8) (Cell Signaling, #9751S, 1:50) and H3K27me3 (C36B11) (Cell Signaling, #9733S, 1:50) according to protocol. For DNA purification, the DNA Purification Buffers and Spin Columns for ChIP and CUT&RUN (Cell Signaling, #14209), with elution in 50 ul. All libraries were quantified on the Qubit 4.0 and library quality assessed using Agilent High Sensitivity DNA Tapestation (Agilent, 5067-5584) before multiplexing and sequencing on a Nextseq 550 Mid Output Kit (150 cycles) with 1% PhiX for a minimum of 5 million paired-end reads per sample with 50bp read length.

DNA methylation.

We used DNA methylation generated from BOS and control blood and fibroblasts and processed as described in (51). We calculated the absolute difference between the means of the β value for BOS patients versus controls for each CpG to obtain the delta beta (Δβ) value using linear regression modeling. We filtered for highly differentially methylated sites (|delta beta (Δβ)| > 5%). Significant CpG sites were identified if they were below FDR < 0.05. To identify biological mechanisms that were dysregulated, we queried these CpG sites using GREAT (13). We filtered for gene ontologies with padj < 0.05 for significance.

RT-qPCR.

10ng of RNA was used per well in 20ul total well volume, and each sample and primer combination was conducted in triplicate. Machine settings were for Standard Curve and Fast run mode. RNA isolated from patient blood and patient-derived fibroblast cell lines were subject to RT-qPCR to validate RNA-seq findings. RNA isolated from transfection experiments were subject to RT-qPCR to identify effects of the ASXL1 truncated plasmids or ASXL1 siRNAs on cell transcriptome.

1. Vogelauer M, et al. Stimulation of Histone Deacetylase Activity by Metabolites of Intermediary Metabolism*. *J. Biol. Chem.* 2012;287(38):32006–32016.

2. Andrews S, Others. FastQC: a quality control tool for high throughput sequence data2010;

3. Dobin A, et al. STAR: ultrafast universal RNA-seq aligner. *Bioinformatics* 2013;29(1):15–21.

4. Liao Y, Smyth GK, Shi W. featureCounts: an efficient general purpose program for assigning sequence reads to genomic features. *Bioinformatics* 2014;30(7):923–930.

5. Love MI, Huber W, Anders S. Moderated estimation of fold change and dispersion for RNA-seq data with DESeq2. *Genome Biol.* 2014;15(12):550.

6. The Gene Ontology resource: enriching a GOld mine. *Nucleic Acids Res.* 2021;49(D1):D325–D334.

7. Ashburner M, et al. Gene ontology: tool for the unification of biology. The Gene Ontology Consortium. *Nat. Genet.* 2000;25(1):25–29.

8. Yu G, et al. clusterProfiler: an R package for comparing biological themes among gene clusters. *OMICS* 2012;16(5):284–287.

9. Heinz S, et al. Simple combinations of lineage-determining transcription factors prime cis-regulatory elements required for macrophage and B cell identities. *Mol. Cell* 2010;38(4):576–589.

10. Li H. Aligning sequence reads, clone sequences and assembly contigs with BWA-MEM. *arXiv [q-bio.GN]* 2013;http://arxiv.org/abs/1303.3997. cited

11. Zhang Y, et al. Model-based analysis of ChIP-Seq (MACS). *Genome Biol.* 2008;9(9):R137.

12. Quinlan AR, Hall IM. BEDTools: a flexible suite of utilities for comparing genomic features.

Bioinformatics 2010;26(6):841–842.

13. McLean CY, et al. GREAT improves functional interpretation of cis-regulatory regions. *Nat. Biotechnol.* 2010;28(5):495–501.

Supplemental Figure 1

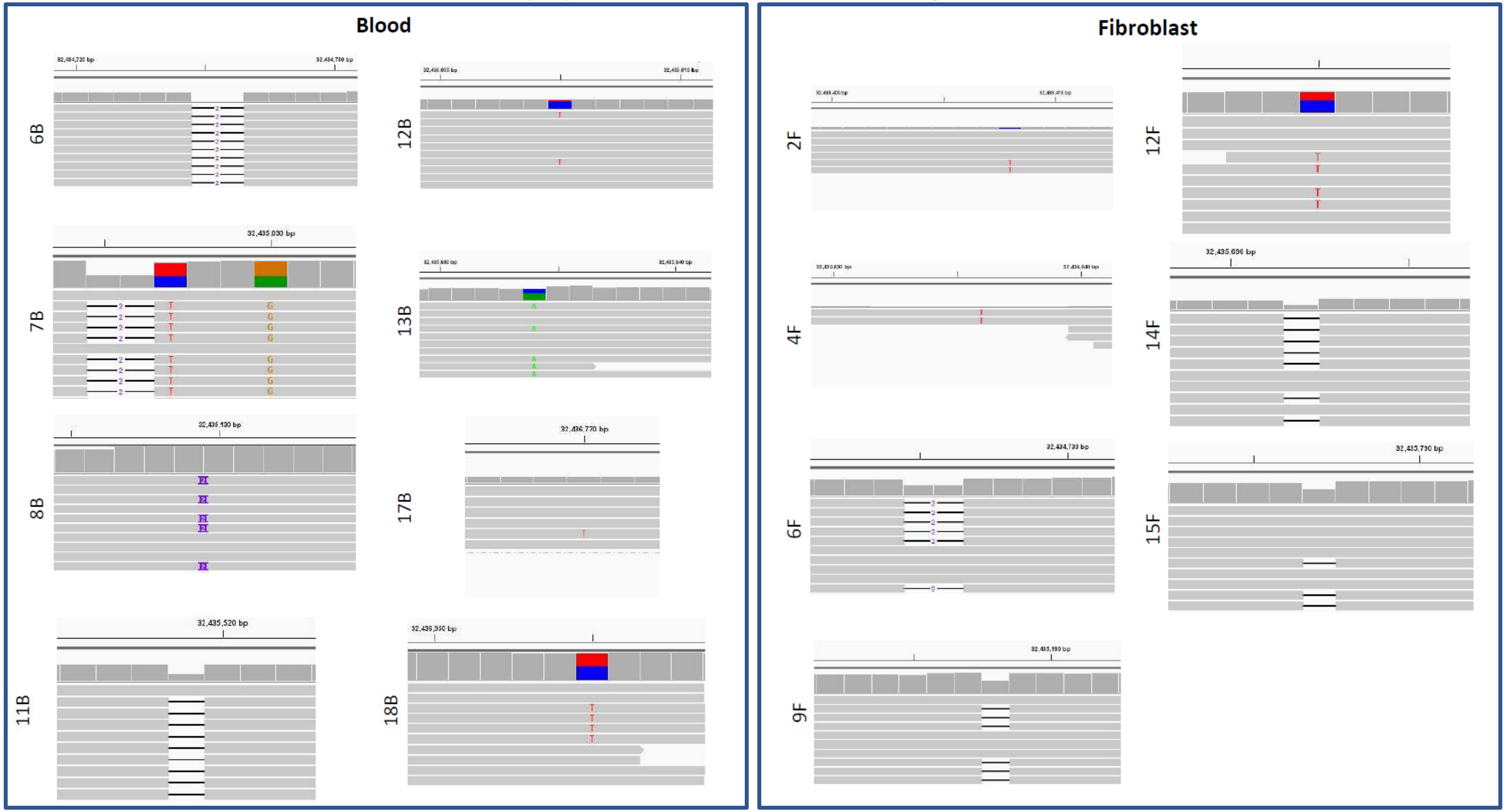

Supplemental Figure 1: Validation of *ASXL1* mutation and identity in BOS Samples.
Representative RNAseq reads (first 10 reads visualised) for each BOS patient blood (n = 8) and BOS fibroblast (n = 7) sample show patient mutation. Patient ID numbers (Figure 1A, Supplemental Table 1) and sample type (F = fibroblast, B = blood) are listed on the left of each insert. Genomic positions for hg38 *ASXL1* (20q11.21) are shown above each insert. For complete coverage and allele count see Supplemental Table 10.

Supplemental Figure 2

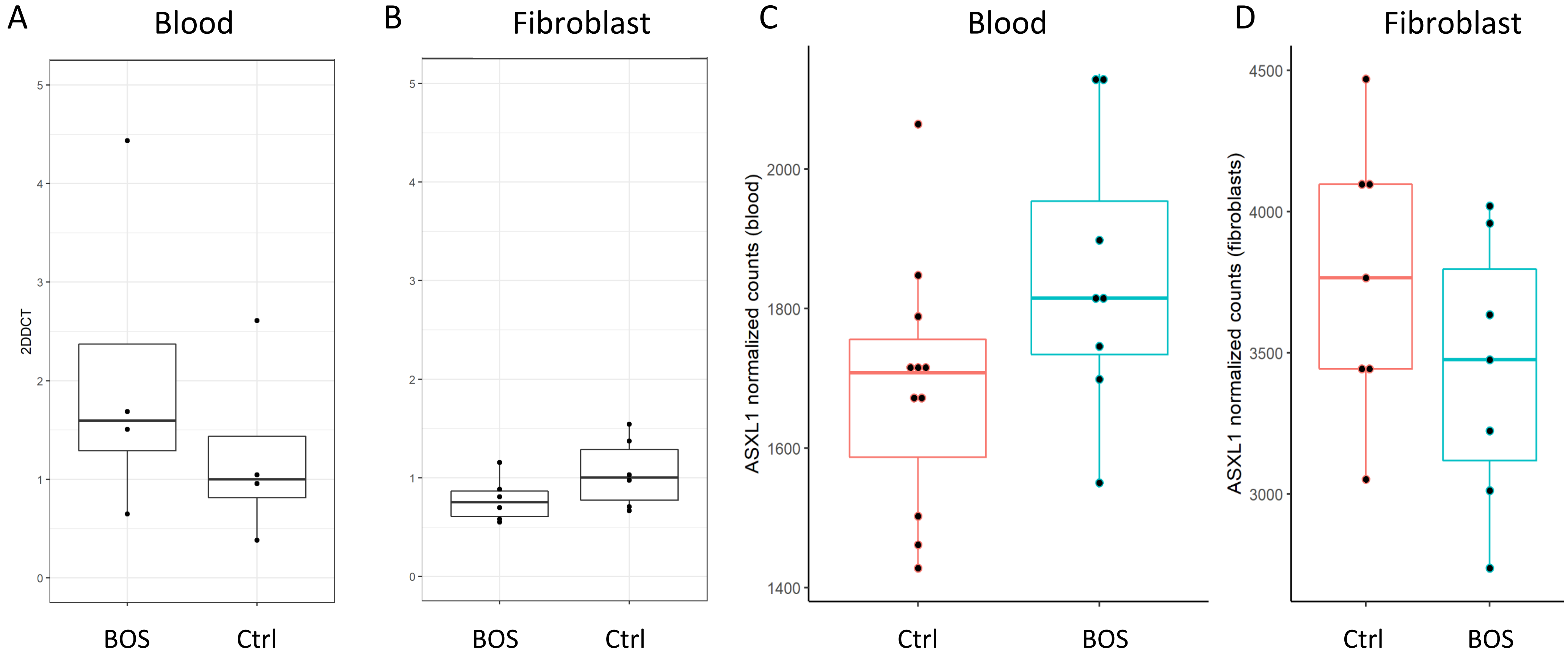

Supplemental Figure 2: Assessment of *ASXL1* expression in patient and control blood and fibroblast samples. Quantitative real-time polymerase chain reaction (qPCR) relative gene expression calculated by the delta-delta Ct method ($2^{-\Delta\Delta Ct}$) do not show significant difference in *ASXL1* expression between (**A**) representative BOS (n = 4) and control (n = 4) blood or (**B**) representative BOS (n = 6) and control (n = 6) fibroblasts. RNA-sequencing DESeq2 normalized counts also do not show significant differential expression of *ASXL1* between (**C**) BOS blood (n = 8) and control blood (n = 11) (log2FC = 0.11, padj = 0.29) or (**D**) BOS fibroblast (n = 7) and control fibroblast (n = 7) (log2FC = -0.01, padj = 0.95). For ASXL1 RNAseq blood, control sample normalized counts ranged from 1428 to 2065, with a mean of 1689, and quartile bounds of 1587 to 1756. BOS normalized sample counts ranged from 1551 to 2136, with a mean of 1848, and quartile bounds of 1734 to 1954. For ASXL1 RNAseq fibroblast, control sample normalized counts ranged from 3052 to 4469, with a mean of 3767, and quartile bounds of 3443 to 4097. BOS normalized sample counts ranged from 2738 to 4020, with a mean of 3438, and quartile bounds of 3118 and 3797.

Supplemental Figure 3

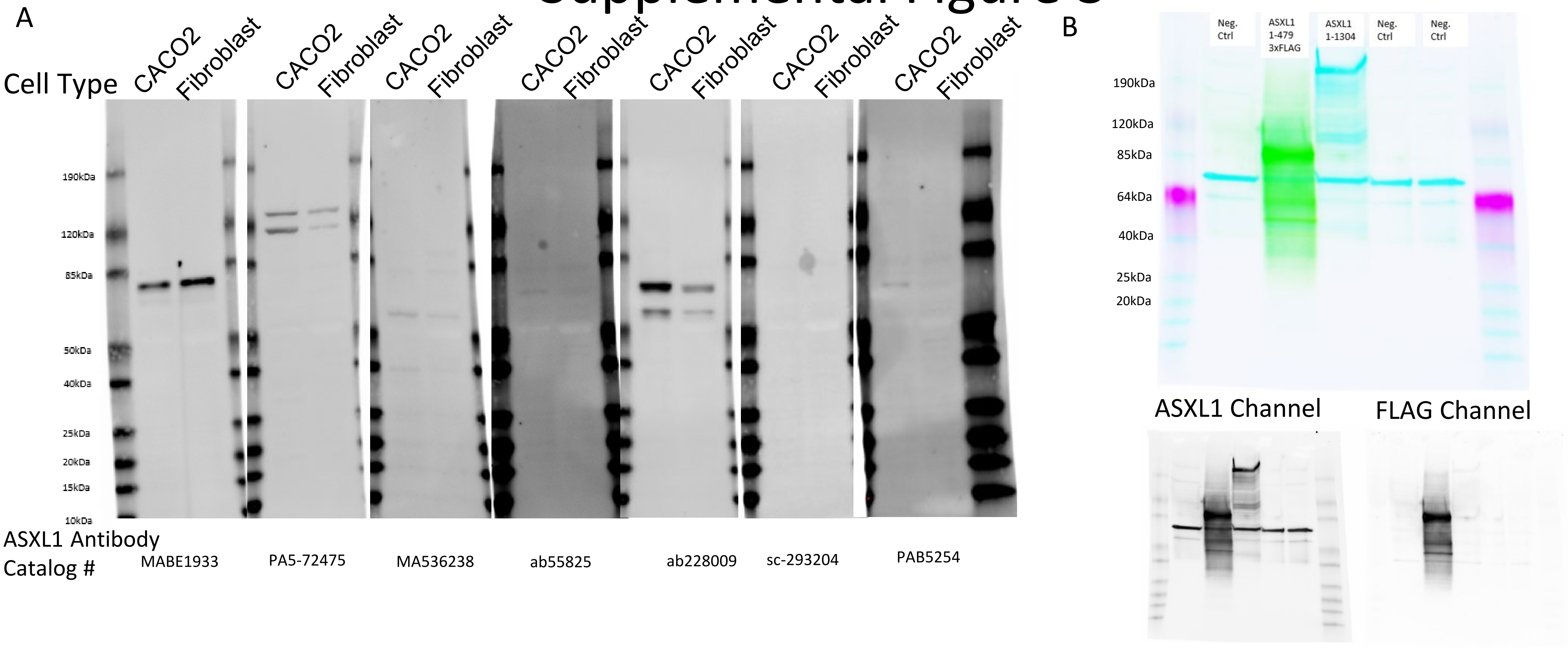

Supplemental Figure 3: ASXL1 Antibody Testing.
(**A**) An array of antibodies targeting *ASXL1* were tested on whole cell lysate (20ug) from CACO2 cells and fibroblast cells. Only antibody #ab228009 identified higher ASXL1 expression in CACO2 cells, which was expected based on ProteinAtlas data (Supplemental Table 4). (**B**) To see whether ASXL1 antibody #ab228009 could identify an exogenous and/or endogenous ASXL1 correctly, we transfected truncated ASXL1 plasmids into HEK293T cells and resolved 10ug nuclear extract on western gel. *ASXL1* antibody #ab228009 identified the truncated ASXL1 plasmids: ASXL1 1-479 + FLAGx3, and ASXL1 1-1304. ASXL1 *(*below, left*)* and FLAG (below, right) staining for the ASXL1 1-479 + FLAGx3 cell lysate showed complete overlap.

Supplemental Figure 4

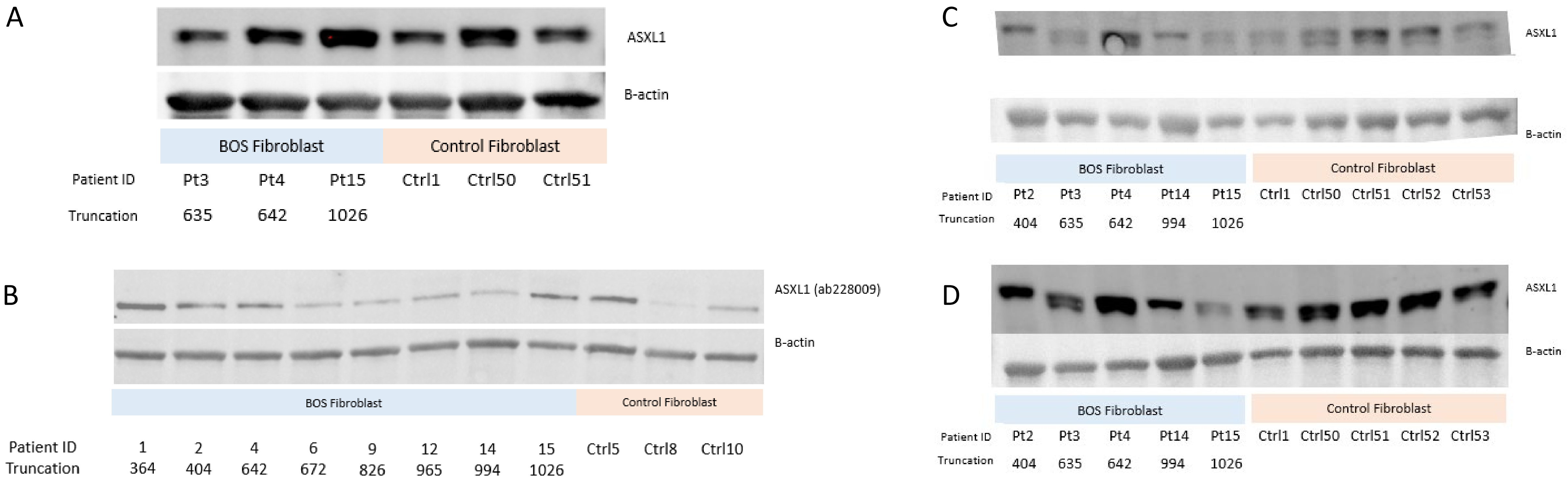

Supplemental Figure 4: Extended ASXL1 (ab228009) western blots.
Whole cell lysate from BOS patient and control fibroblast were stained with ASXL1 primary antibody (ab228009). These representative blots show (**A**) 30ug of BOS n=3 and control n=3, (**B**) 15ug of BOS n=8 and control n=3, and (**C, D**) 15ug of BOS n=5 and control n=5. No visible or quantifiable difference was identified between BOS and control histone marks (**Supplemental Table 7**).

Supplemental Figure 5

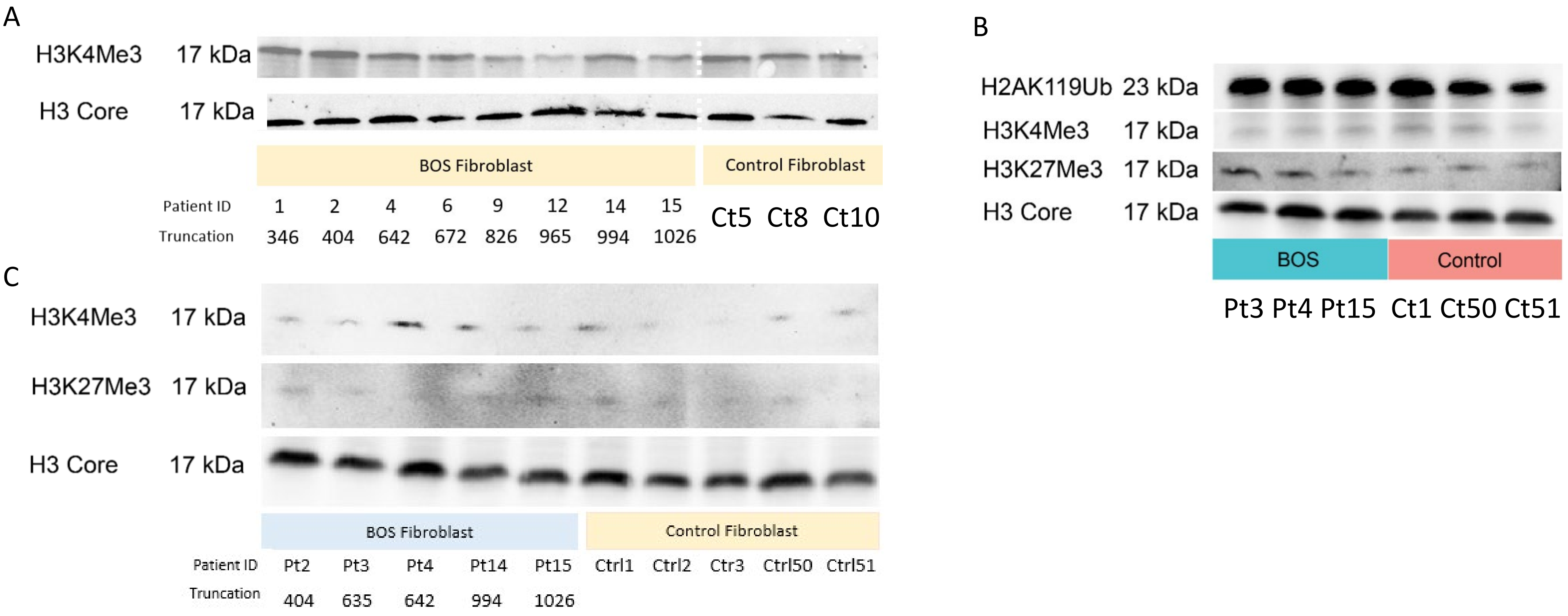

Supplemental Figure 5: Extended histone H2AK119ub and H3K4me3 western blots.
Histone extracts (1ug) from BOS patient and control fibroblast were stained with (**A**) H3K4me3, (**B**) H2AK119ub, H3K4me3 and H3K27me3, and (C) H3K4me3 and H3K27me3 primary antibodies. No visible or quantifiable difference was identified between BOS and control histone marks (**Supplemental Table 7**).

Supplemental Figure 6

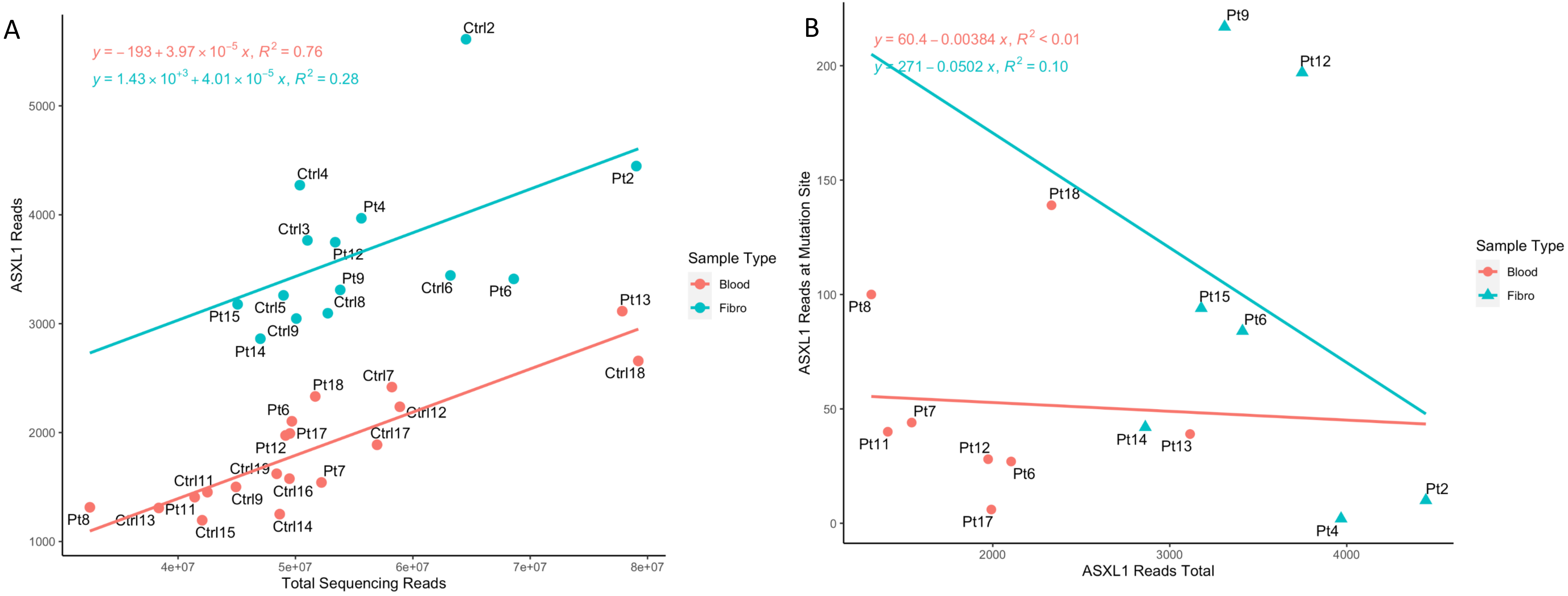

Supplemental Figure 6: Sequencing Coverage and ASXL1 Read Count

A) RNA-seq ASXL1 reads were plotted against total sequencing coverage. Blood samples (red) showed a strong correlation ($R^2 = 0.76$) between increased ASXL1 reads as total sequencing reads increased. Fibroblast samples (blue) had higher ASXL1 read count at the same sequencing coverage as blood samples, and the same positive correlation between ASXL1 read count and sequencing coverage. (**B**) For each RNA-seq BOS sample, the number of reads at the BOS-causing mutation site was identified (Supplemental Table 10) and plotted against total ASXL1 reads for that sample. No strong correlation was identified in either blood (red) or fibroblast (blue) samples.

Supplemental Figure 7

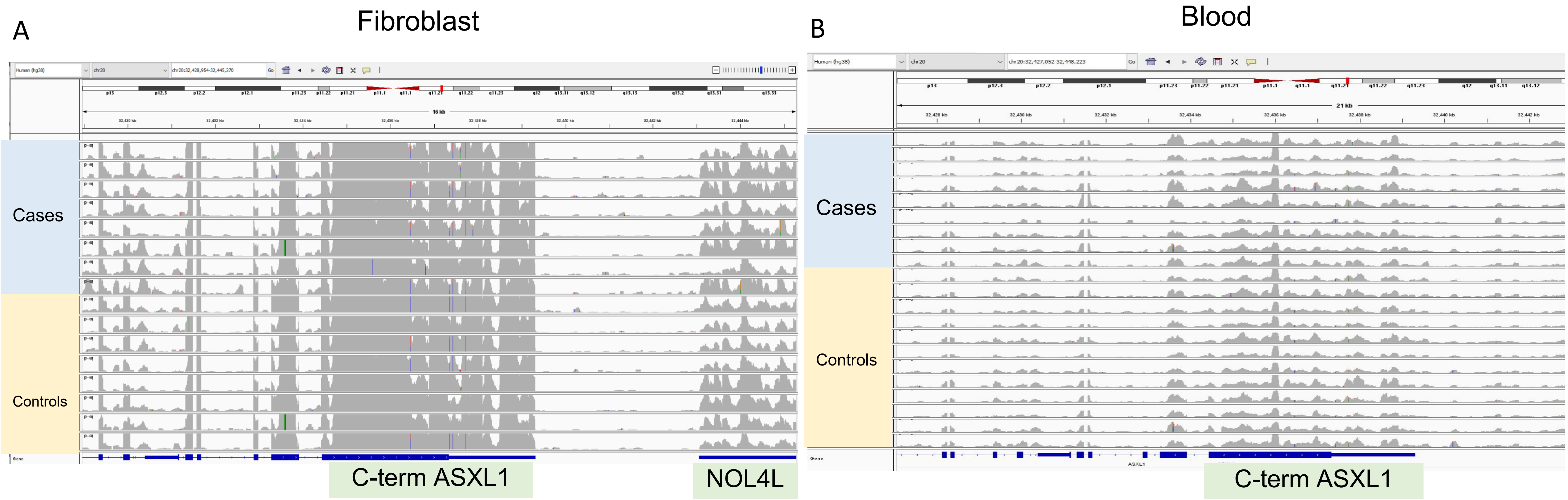

Supplemental Figure 7: Normalized ASXL1 reads in BOS patients and controls
Integrative Genome Viewer is used to show normalized read counts for ASXL1 in BOS patients and controls in (**A**) fibroblast samples and (**B**) blood samples. Similar normalized levels of ASXL1 are observed for BOS cases and controls in both tissue types at the C-terminus.

Supplemental Figure 8

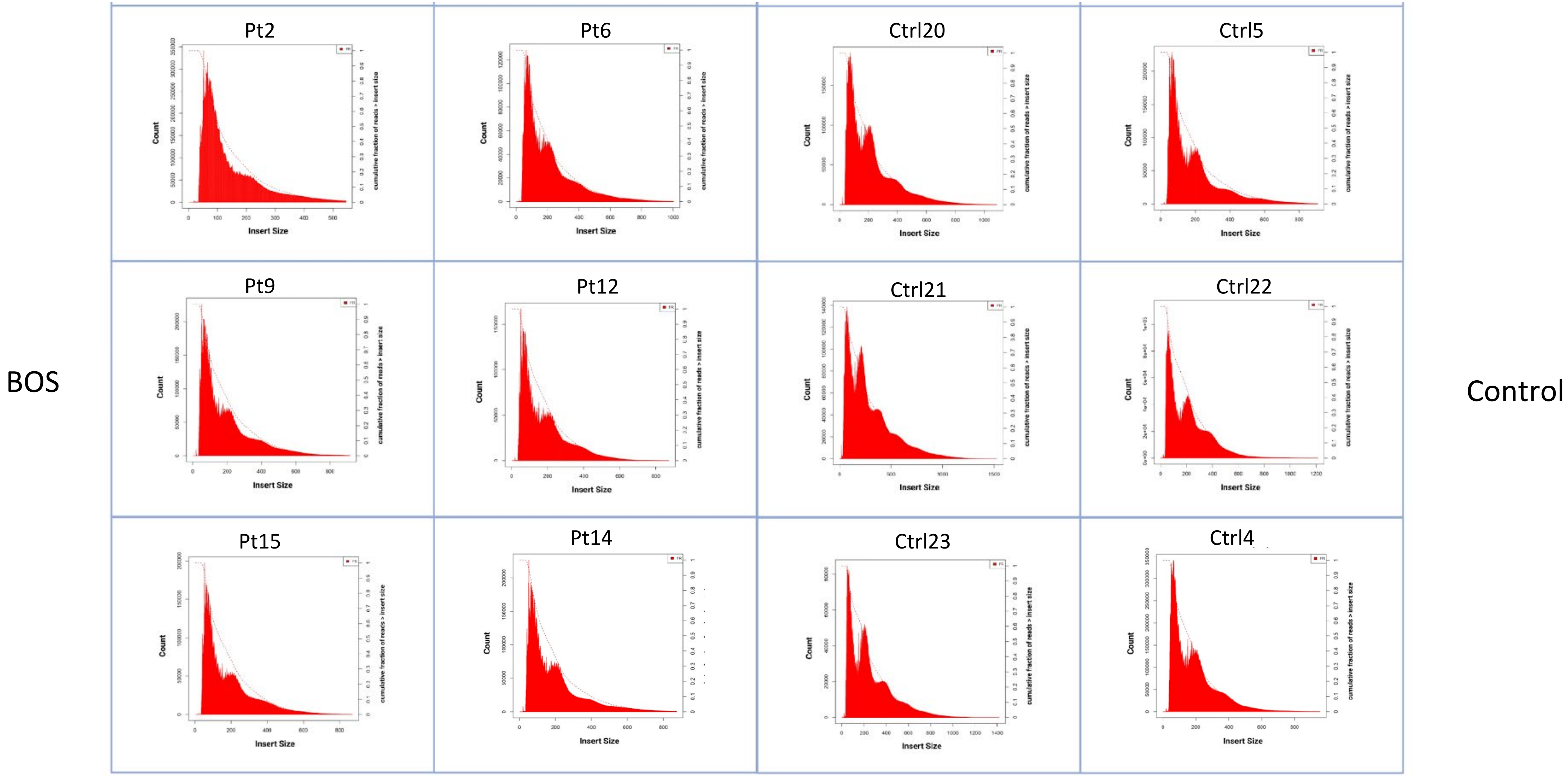

Supplemental Figure 8: ATAC-seq Sample Fragment Length

Fragment length distribution plots for each ATAC-seq sample (BOS n = 6, control n = 6) are shown. We identify more short fragment lengths in each sample, which correspond to more open chromatin, and decreasing amounts of large fragments, which correspond to less open chromatin.

Supplemental Figure 9

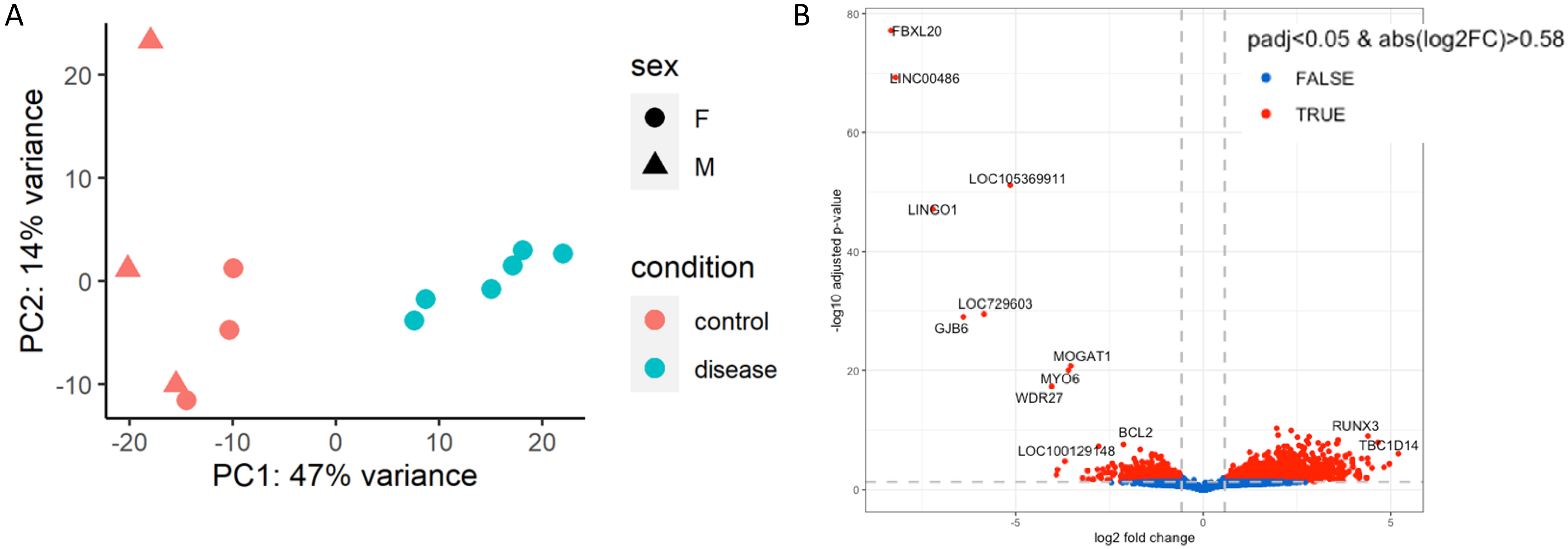

Supplemental Figure 9: ATACseq Fibroblast Library QC.
(**A**) Principal component analysis with adjustment for sex identifies strong separation of BOS (n=6) and control (n=6) samples.
(**B**) Volcano plot of ATAC-seq BOS fibroblast compared to control samples identifies differential chromatin openness. We adjusted for the covariate of sex, and identified 4336 differential peaks at our padj < 0.05 cutoff mapping to 3054 unique genes, with 3036 peaks (70.0%) more accessible in BOS samples than controls

Supplemental Figure 10

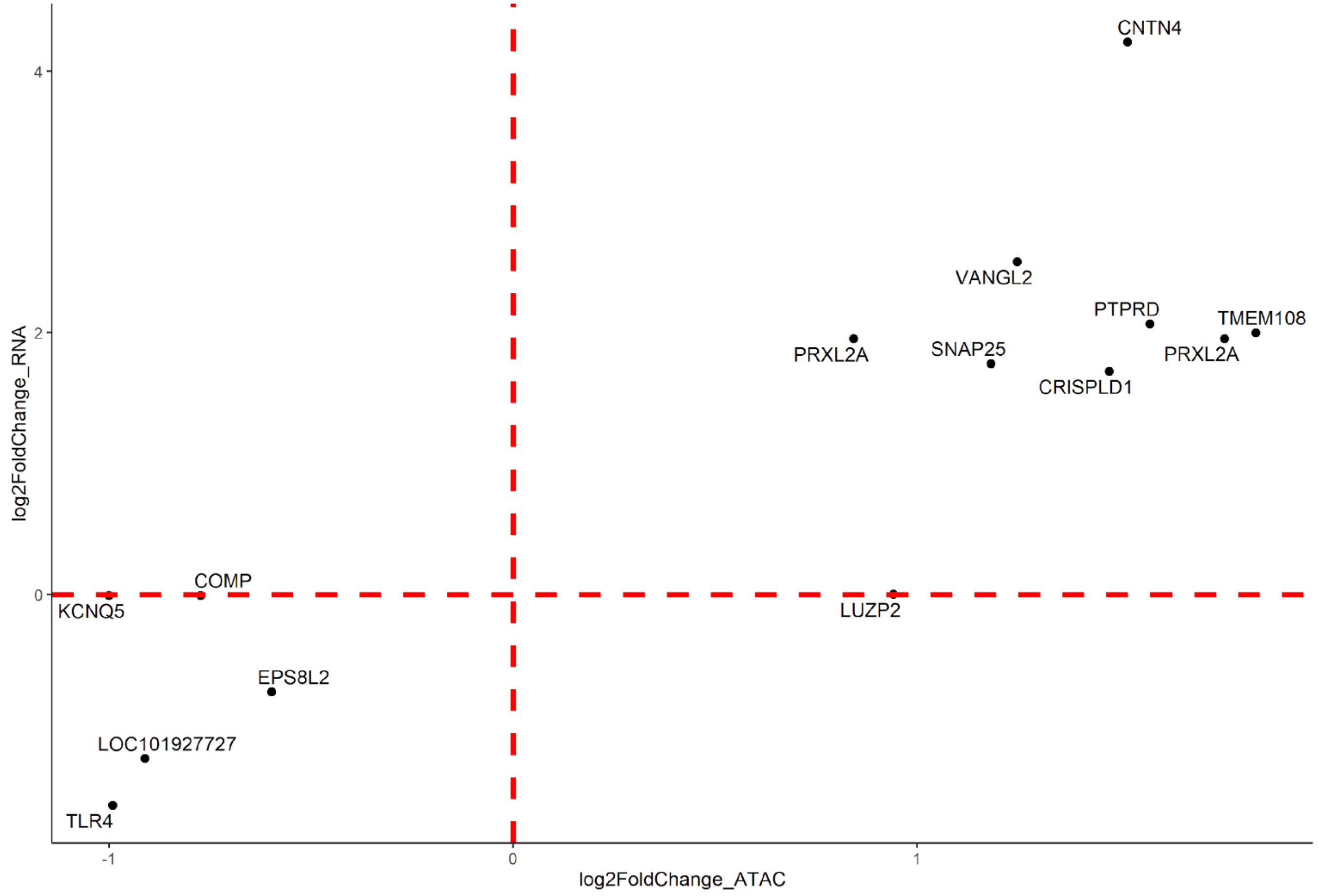

Supplemental Figure 10: Integration of differential open chromatin at transcriptional start sites with transcriptomic dysregulation in BOS fibroblasts.

Significant DEGs from BOS RNAseq fibroblast data was integrated with significant differential chromatin accessibility peaks at the transcriptional start site from BOS ATACseq fibroblast data. DEGs that overlapped are shows with the log_2FC for RNAseq (y axis) and log_2FC for ATACseq (x axis).

Supplemental Figure 11

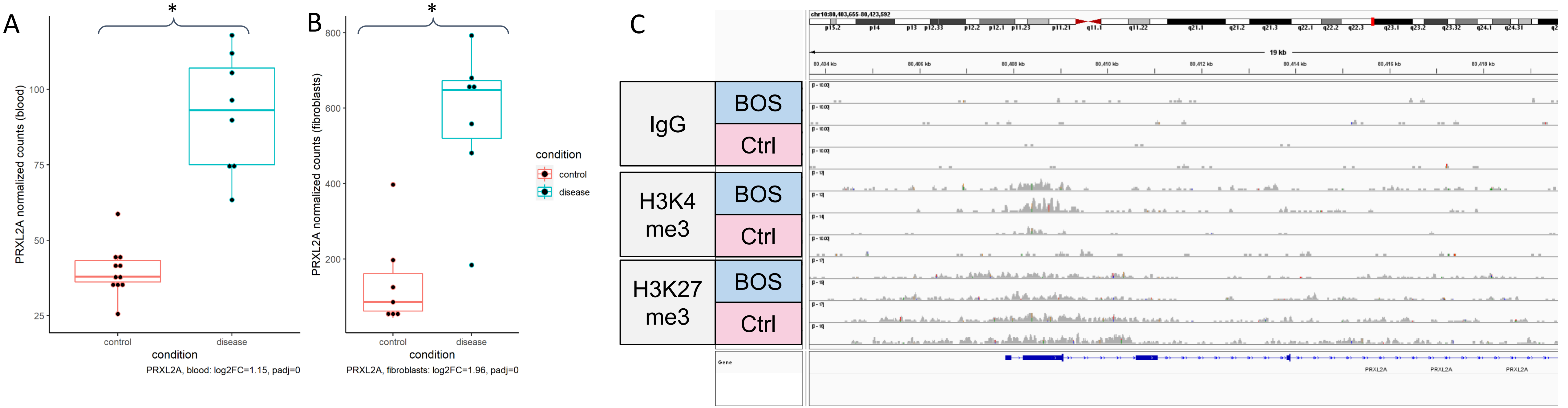

Supplemental Figure 11: Multi-ome integration identifies *PRXL2A* dysregulation in BOS samples. RNA-sequencing DESeq2 normalized counts show significant differential expression of *PRXL2A* between BOS and control samples in (**A**) blood (log2FC = 1.15, padj = 0) or (**B**) fibroblasts (log2FC = 1.96, padj = 0). (**C**) CUT&RUN identifies increased H3K4me3 binding at the *PRXL2A* transcriptional start site.

Supplemental Figure 12

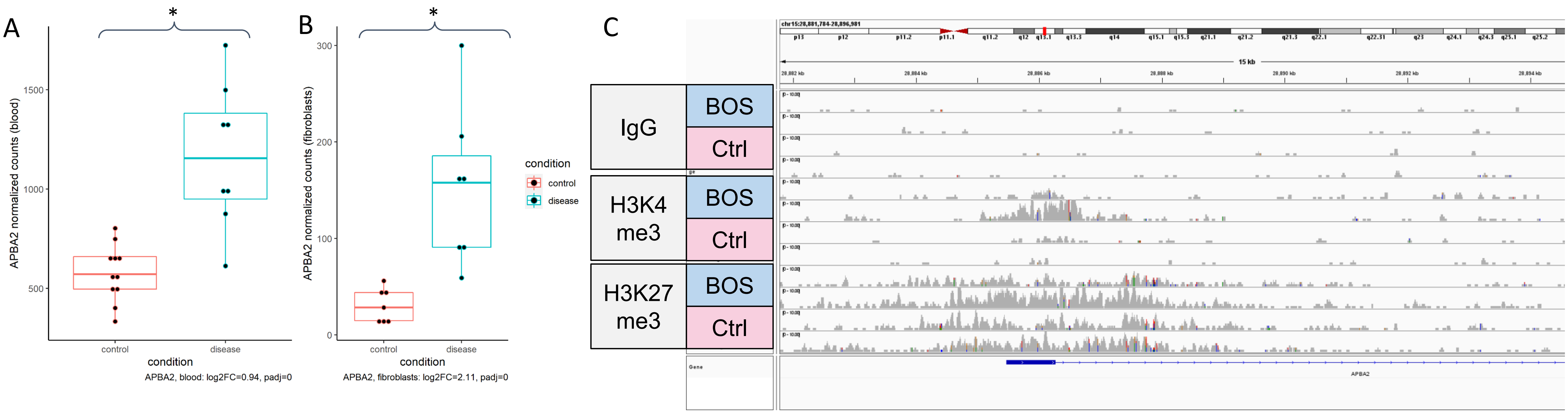

Supplemental Figure 12: Multi-ome integration identifies *APBA2* dysregulation in BOS samples. RNA-sequencing DESeq2 normalized counts show significant differential expression of *APBA2* between BOS and control samples in (**A**) blood (log2FC = 0.94, padj = 0) or (**B**) fibroblasts (log2FC = 2.11, padj = 0). (**C**) CUT&RUN identifies increased H3K4me3 binding at the *APBA2* transcriptional start site.

Supplemental Figure 13

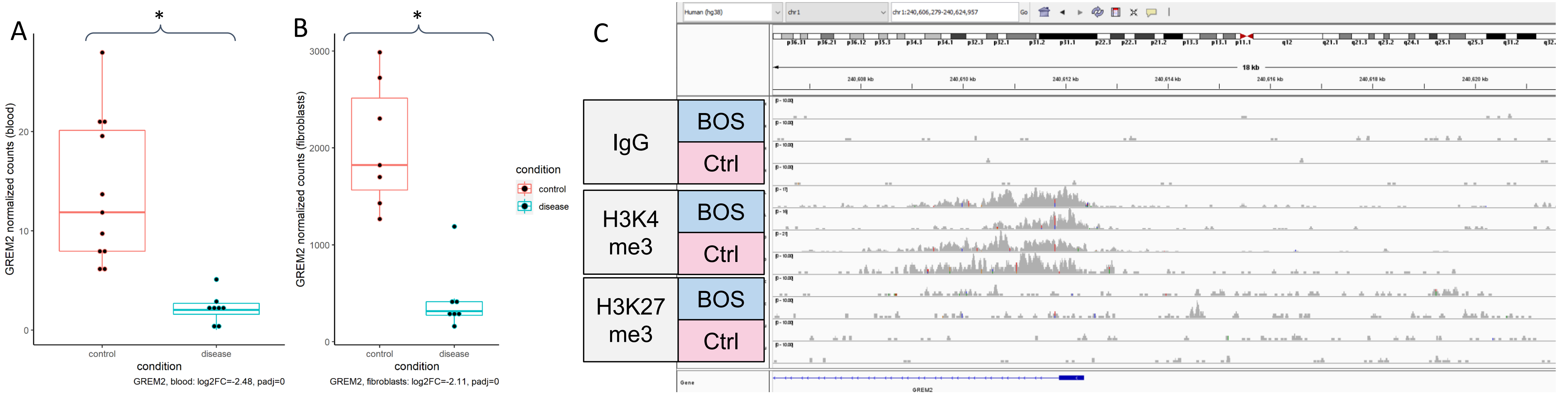

Supplemental Figure 13: Multi-ome integration identifies *GREM2* dysregulation in BOS samples. RNA-sequencing DESeq2 normalized counts show significant differential expression of *GREM2* between BOS and control samples in (**A**) blood (log2FC = -2.48, padj = 0) or (**B**) fibroblasts (log2FC = -2.11, padj = 0). (**C**) CUT&RUN identifies increased H3K4me3 binding at the *GREM2* transcriptional start site.

Supplemental Figure 14

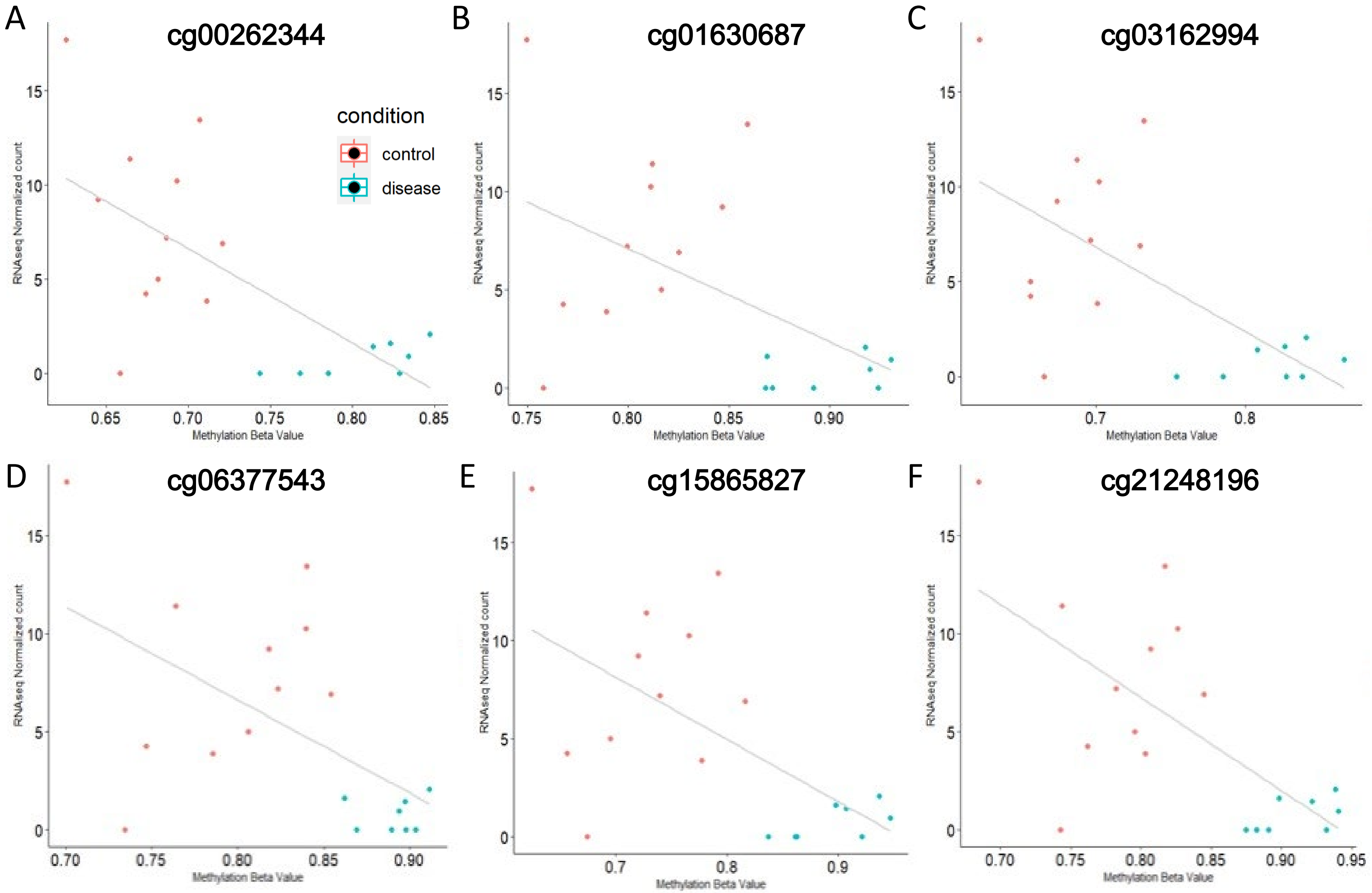

Supplemental Figure 14: Differentially methylated CpG sites at *PSMA8* in BOS patient blood.
BOS patient (blue) and control (red) samples transcriptomic normalized values from blood RNAseq (x axis) and methylation beta values from blood DNAm (y axis) were plotted for CpG site (**A**) cg00262344 (**B**) cg01630687 (**C**) cg03162994 (**D**) cg06377543 (**E**) cg15865827 (**F**) cg21248196. At each CpG sites, BOS patient samples were hypermethylated and transcriptionally downregulated compared to controls. (**G**) RNAseq did not identify *PSMA8* reads in fibroblast samples.

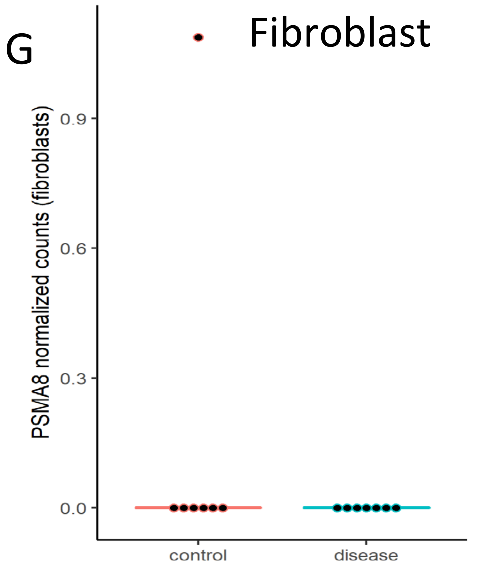

Supplemental Figure 15

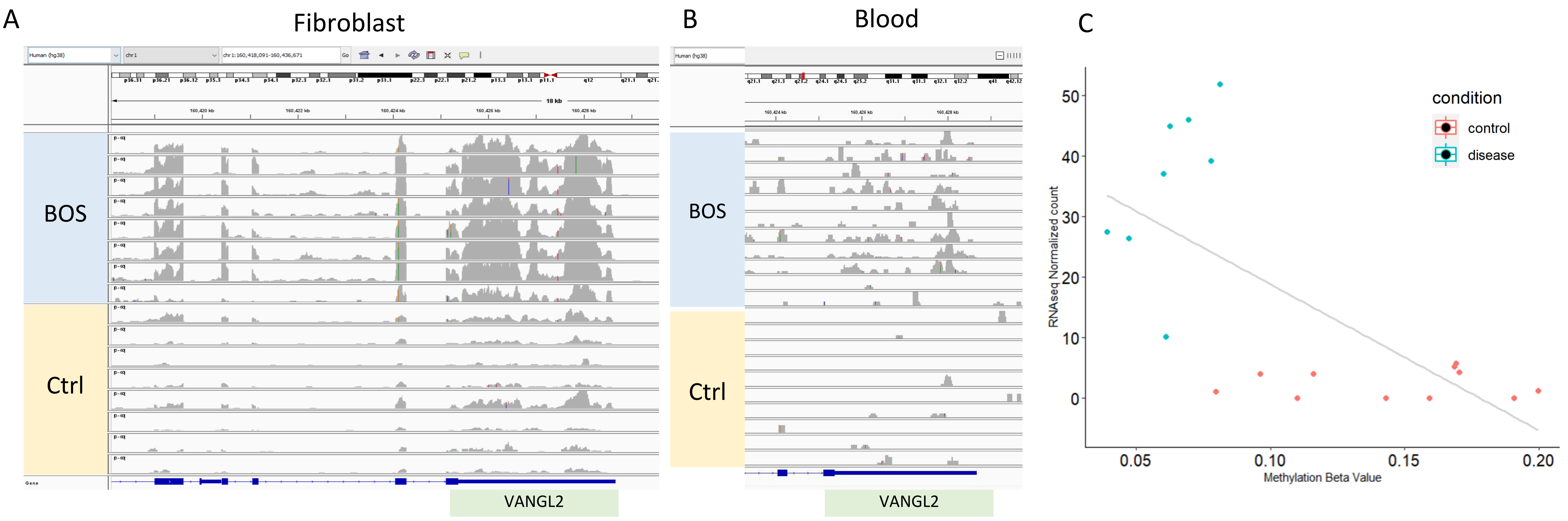

Supplemental Figure 15: *VANGL2* is one of the most highly overexpressed transcripts in BOS patient samples. Normalized *VANGL2* reads in BOS patients and controls (**A**) fibroblast samples and (**B**) blood samples show increased expression in BOS samples. (**C**) At CpG site cg17024258 located at the *VANGL2* transcriptional start site, BOS patient samples are hypomethylated (Δβ -7.6%) and transcriptionally upregulated compared to controls.

Supplemental Figure 16

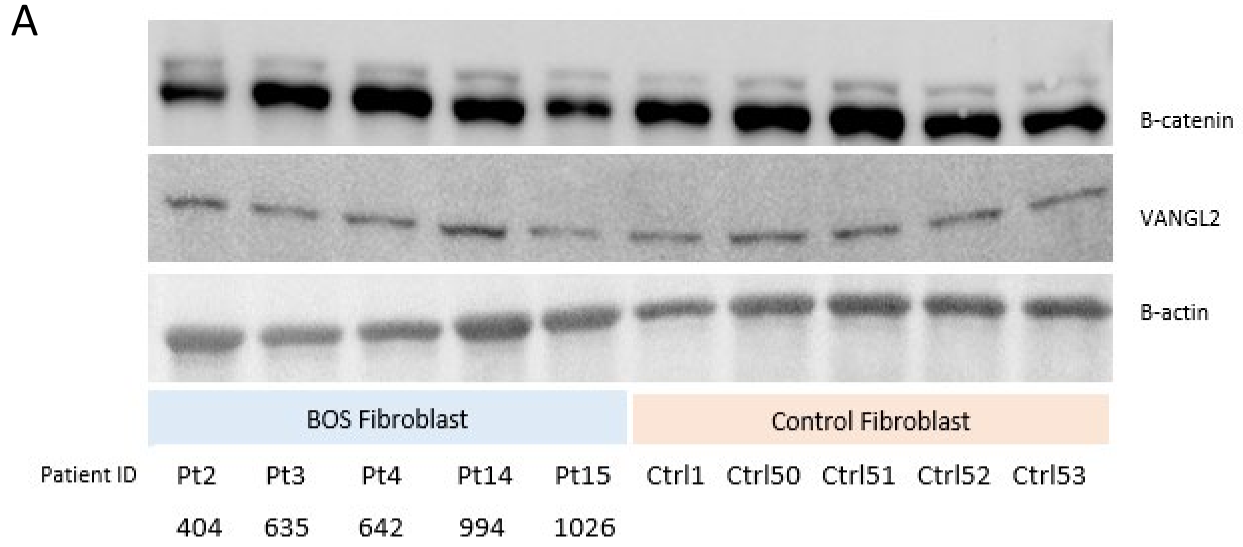

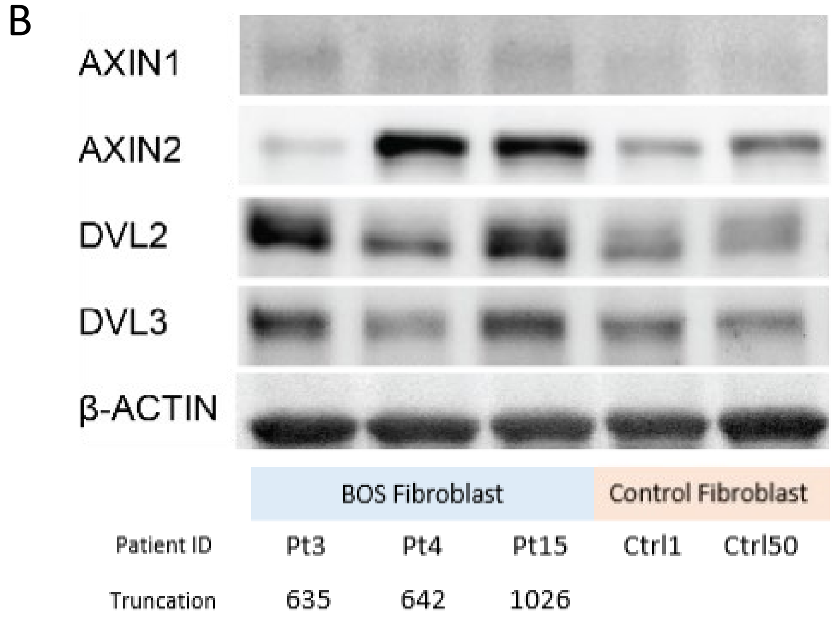

Supplemental Figure 16: Extended Wnt signaling pathway blots.
Whole cell lysate from BOS patient and control fibroblast were stained with (**A**) B-catenin and VANGL2 using 15ug of BOS n=5 and control n=5 fibroblast samples per lane. (B) 30ug of BOS n=3 and control n=3 fibroblast samples were also stained with AXIN1, AXIN2, DVL2, and DVL3.

Supplemental Figure 17

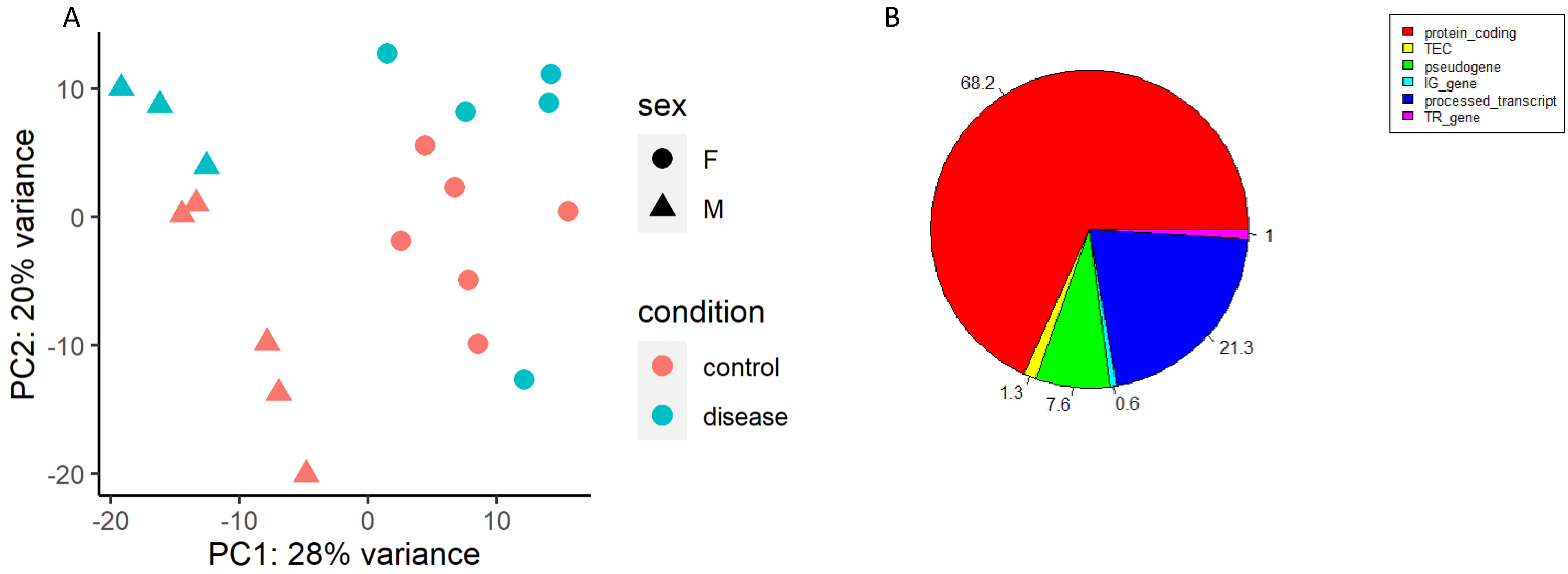

Supplemental Figure 17: RNAseq Blood Library QC.
(**A**) Principal component analysis with adjustment for sex identifies moderate separation of patient and control samples. (**B**) Mapping of reads to transcripts identified a high percentage corresponding to protein-coding genes (68.2%)

Supplemental Figure 18

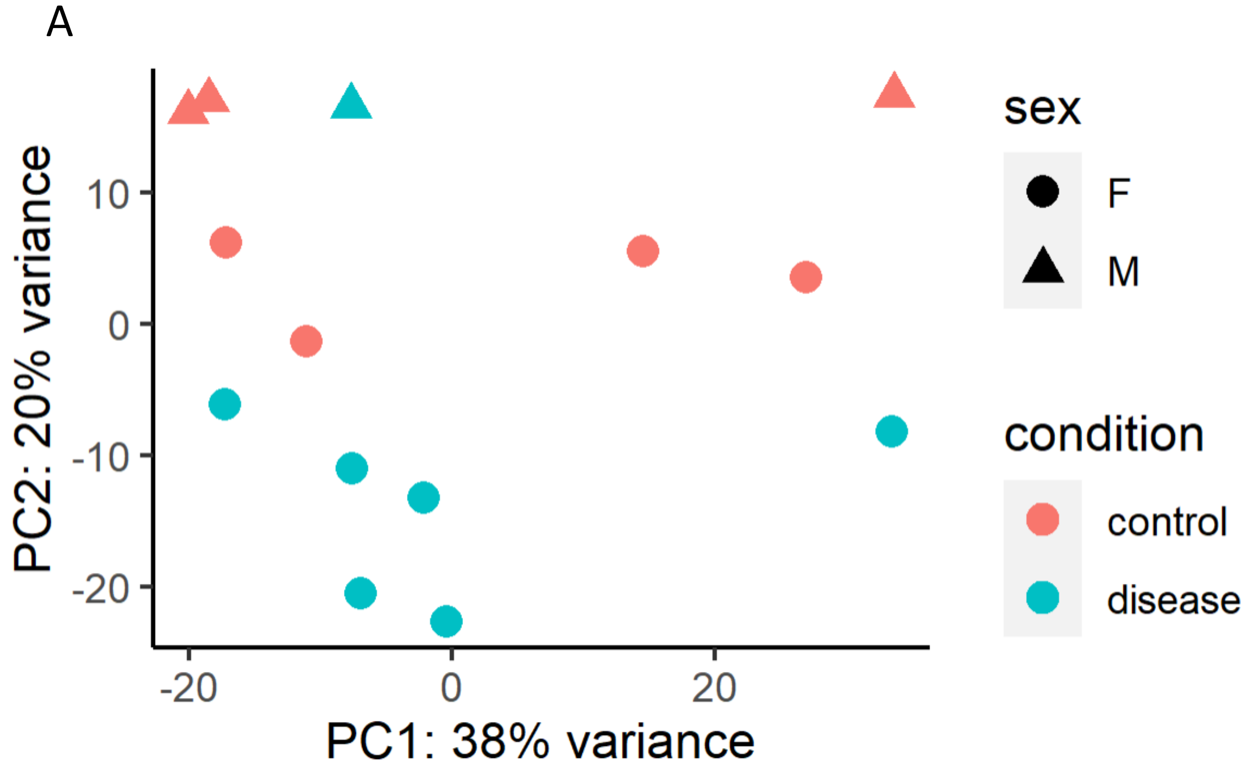

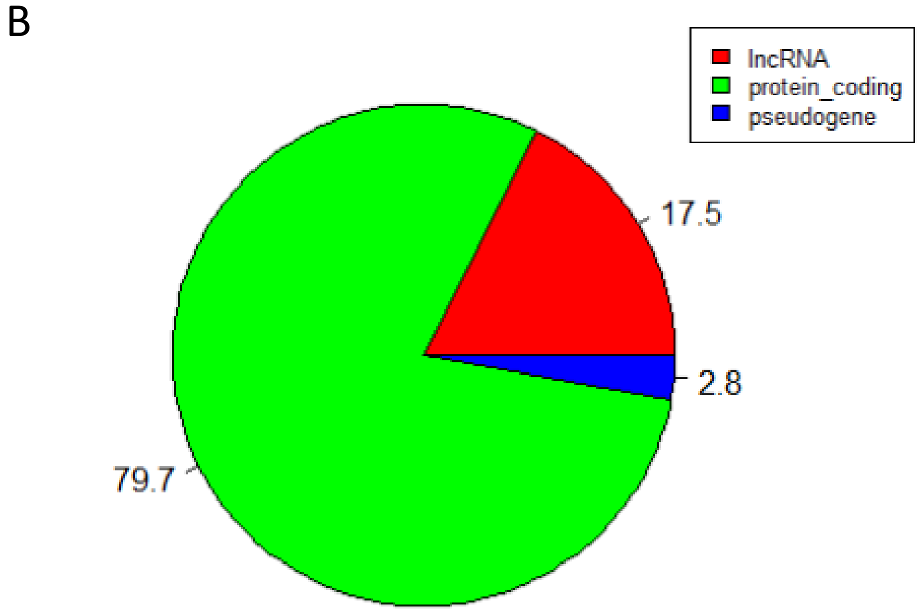

Supplemental Figure 18: RNAseq Fibroblast Library QC.
(**A**) Principal component analysis with adjustment for sex identifies moderate separation of patient and control samples. (**B**) Mapping of reads to transcripts identified a high percentage corresponding to protein-coding genes (79.7%)

Supplemental Figure 19

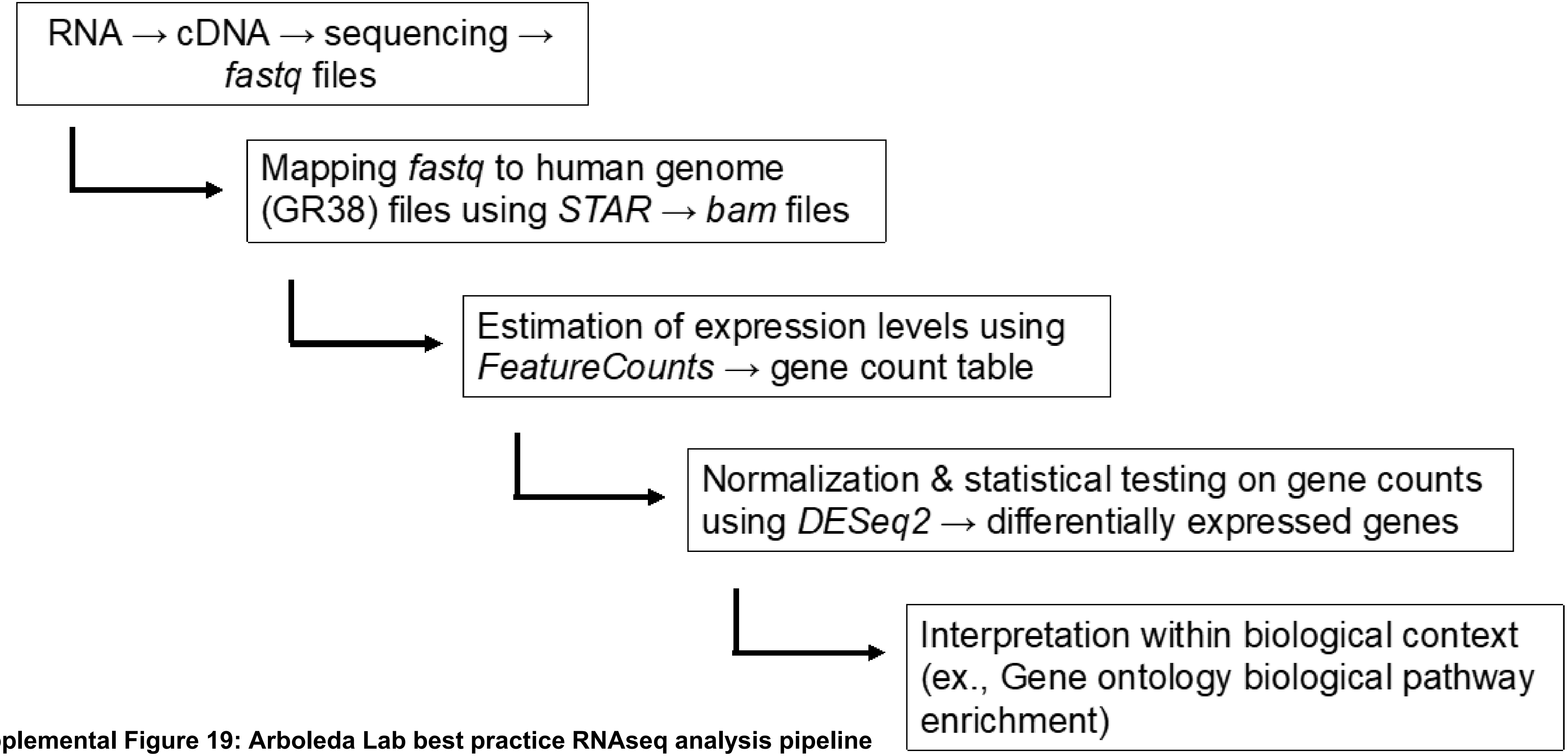

Supplemental Figure 19: Arboleda Lab best practice RNAseq analysis pipeline

Supplemental Figure 20

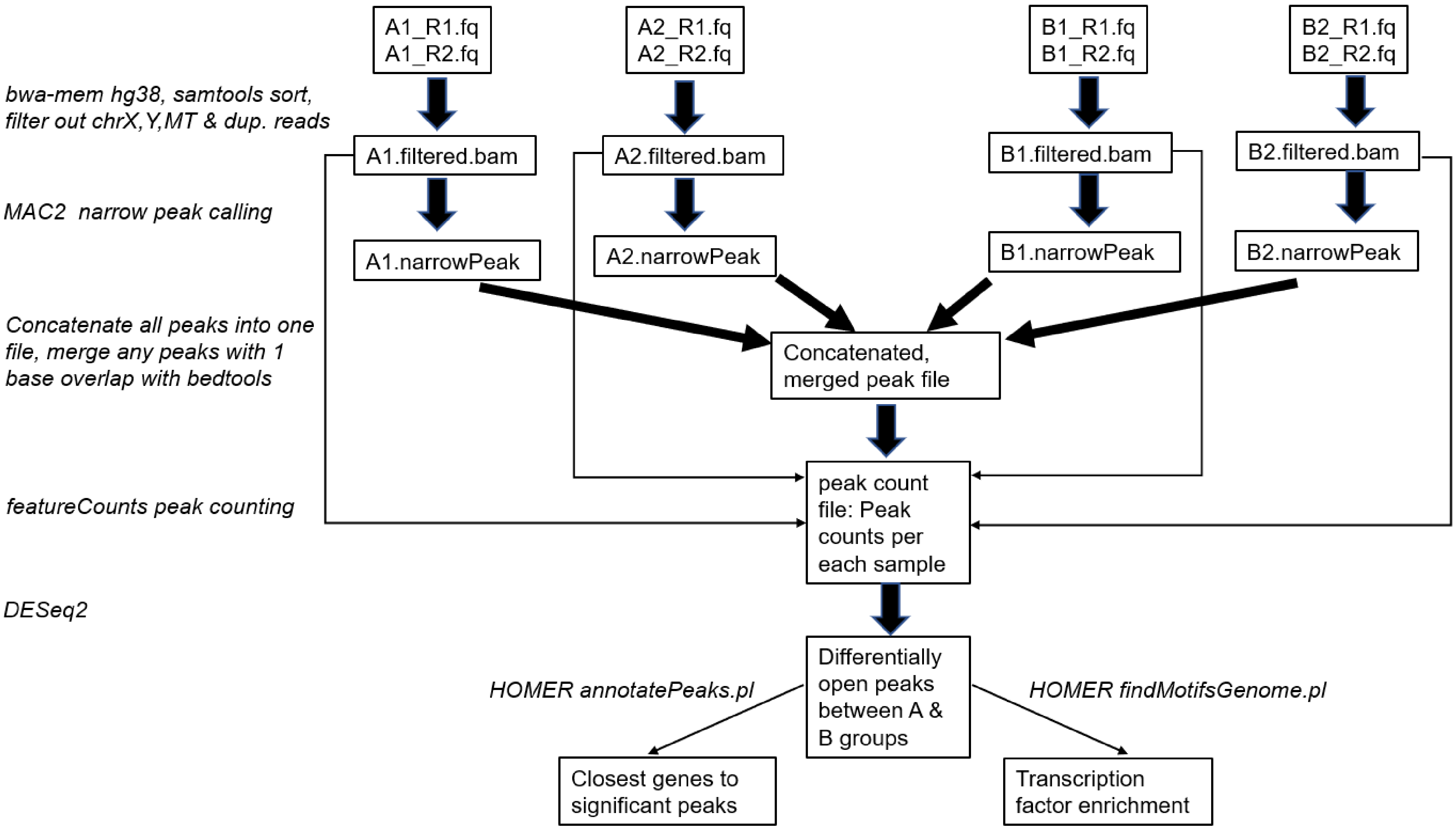

Supplemental Figure 20: Arboleda Lab best practice ATACseq analysis pipeline

SUPPLEMENTAL TABLE LEGENDS

Supplemental Table 1: Bohring-Opitz Syndrome (BOS) Patient Information and Assays
Bohring-Opitz Syndrome (BOS) patients included in our assays are identified with their patient IDs. We include mutation annotation, age, and sex, and outline the multi-omics assays that each patient's samples were used in.

Supplemental Table 2: Control Sample Information and Assays
Control samples used in our assays are shown with their age and sex. We outline the multi-omics assays that each sample was used in.

Supplemental Table 3: BOS Patient Summary Table
We show summary data (age, sex, mutation) for BOS patients in our cohort that are included in each -omics assay.

Supplemental Table 4: Expression of Key Genes in Tissues and Cell Lines
Transcripts per million (TPM) gene expression are summarized for key genes of interest. We obtained data for gene expression in relevant tissues from GTEx and ProteinAtlas, and data for cell lines from ProteinAtlas.

Supplemental Table 5: RT-qPCR Primers
PrimeTime™ RT-qPCR Primers were purchased from IDT to assay ASXL1 expression. Pre-designed primer pairs and fluorescently labeled 5′ nuclease probe that were used in our assays are listed. These are two primer sets for ASXL1, each targeting a different exonic range. and one set targeting B-actin (ACTB) which was used as a control.

Supplemental Table 6: List of Antibodies
Antibodies used in this paper are shown here with their purchasing information.

Supplemental Table 7: ImageJ Quantification of Western Blots
ImageJ quantification and calculations for western blots in Figures 1E, 1F, 5B, and 5C, Supplemental Figures 4, 5 and 16, and Supplemental uncropped, unedited blots.

Supplemental Table 8: RNA-seq Fibroblast QC and Mapping Statistics
BOS patient and control -derived fibroblasts were used in RNA-seq. Here, we show FastQC and mapping statistics for each sample.

Supplemental Table 9: RNA-seq Blood QC and Mapping Statistics
BOS patient and control blood were used in RNA-seq. Here, we show FastQC and mapping statistics for each sample.

Supplemental Table 10: ASXL1 Pathogenic and Wild-type Allele Counts in BOS patients.
RNA-seq read counts for the ASXL1 gene in BOS patients was determined. Each read was designated pathogenic if it included the truncating mutation associated with that patient, and wild-type if it did not.

Supplemental Table 11: Significant Differentially Expressed Genes in BOS Fibroblasts Identified through RNA-seq
DESeq2 was used to analyze RNA-seq gene counts from our cohort of BOS and control fibroblast samples. Samples were adjusted for the co-variate of sex based on principal component analysis. Genes were considered significant if Bonferroni adjusted $p < 0.05$ and are listed here with their adjusted p value, log_2Fold Change, and gene annotations.

Supplemental Table 12: Significant Differentially Expressed Genes in BOS Blood Identified through RNA-seq
DESeq2 was used to analyze RNA-seq gene counts from our cohort of BOS and control blood samples. Samples were adjusted for the co-variate of sex based on principal component analysis. Genes were considered significant if Bonferroni adjusted $p < 0.05$ and are listed here with their adjusted p value, log_2Fold Change, and gene annotations.

Supplemental Table 13: Gene Ontology Analysis for BOS Blood RNA-seq
Gene ontology (GO) over-enrichment tests were performed using all significant differentially expressed genes in BOS blood samples from our RNA-seq assay. P-values shown are the probability of seeing at least x number of genes out of the total input genes annotated to a particular GO term, given the proportion of genes in the whole genome that are annotated to that GO term. We filtered for gene ontologies with padj < 0.05 for significance.

Supplemental Table 14: Gene Ontology Analysis for BOS Fibroblast RNA-seq
Gene ontology (GO) over-enrichment tests were performed using all significant differentially expressed genes in BOS fibroblast samples from our RNA-seq assay. P-values shown are the probability of seeing at least x number of genes out of the total input genes annotated to a particular GO term, given the proportion of genes in the whole genome that are annotated to that GO term. We filtered for gene ontologies with padj < 0.05 for significance.

Supplemental Table 15: ATACseq Fibroblast QC and Mapping Statistics
BOS patient and control -derived fibroblasts were used in ATAC-seq. Here, we show FastQC, fragment length, and mapping statistics for each sample.

Supplemental Table 16: Significant Differentially Expressed Genes in BOS Fibroblasts Identified through ATAC-seq
DESeq2 was used to analyze ATACseq gene counts from our cohort of BOS and control fibroblast samples. Samples were adjusted for the co-variate of sex based on principal component analysis. Genes were considered significant if Bonferroni adjusted $p < 0.05$ and are listed here with their adjusted p value, log_2Fold Change, and gene annotations.

Supplemental Table 17: Unique Gene List for Differentially Expressed Genes in BOS Fibroblast ATAC-seq
Unique genes identified in BOS fibroblast ATAC-seq analysis are listed here with the corresponding number of unique differentially regulated chromatin peaks per gene at each filtering cutoff.

Supplemental Table 18: Gene Ontology Analysis for BOS Fibroblast ATAC-seq
Gene ontology (GO) over-enrichment tests were performed using all significant differentially expressed genes in BOS fibroblast samples from our ATAC-seq assay. P-values shown are the probability of seeing at least x number of genes out of the total input genes annotated to a particular GO term, given the proportion of genes in the whole genome that are annotated to that GO term. We filtered for gene ontologies with $padj < 0.05$ for significance.

Supplemental Table 19: Motif Enrichment Analysis of ATACseq DEGs in BOS Fibroblast
HOMER (Hypergeometric Optimization of Motif EnRichment) was used to analyze motif enrichment of our BOS ATAC-seq fibroblast data using both known motif and de novo methods.

Supplemental Table 20: Overlapping DEGs Identified through Chromatin Accessibility and Transcriptional Dysregulation in BOS Fibroblasts
Bonferroni adjusted $p < 0.05$ significant differentially expressed genes (DEGs) from our fibroblast RNA-seq dataset (Supplemental Table 11) and our fibroblast ATAC-seq (Supplemental Table 16) datasets were integrated to identify common dysregulated genes. We identified 25 DEGs common between the two tissue types, with 21 of these genes dysregulated in the same direction.

Supplemental Table 21: Significant Differentially Methylated CpG Sites in BOS Blood Identified through DNA methylation
Significant CpG sites between BOS patient blood and control blood were identified if they were below $FDR < 0.05$. The absolute difference between the means of the β value for BOS patients versus controls for each CpG was calculated to obtain the delta beta ($\Delta\beta$) value. We filtered for highly differentially methylated sites (|delta beta ($\Delta\beta$)| > 10%) using linear regression modeling. Genes were considered significant if below $FDR < 0.05$ and are listed here with their adjusted p value, $\Delta\beta$, and gene annotations.

Supplemental Table 22: Unique Gene List for Differentially Expressed Genes in BOS Blood DNA methylation
Unique genes identified in BOS blood DNA methylation analysis are listed here with the corresponding number of unique differentially methylated CpG peak per gene at each filtering cutoff.

Supplemental Table 23: Overlapping DEGs between RNA-seq and DNA methylation analysis of BOS blood
Bonferroni adjusted $p < 0.05$ significant differentially expressed genes (DEGs) from our blood RNA-seq (Supplemental Table 12) and our blood DNA methylation (Supplemental Table 21)

datasets were integrated to identify common dysregulated genes. We identified 341 DEGs common between the two assay types.

Supplemental Table 24: Differentially Methylated CpG Sites in BOS Fibroblast Identified through DNA methylation

CpG sites between BOS patient fibroblast and control fibroblast were identified if they were below a nominal p-value of 0.005. The absolute difference between the means of the β value for BOS patients versus controls for each CpG was calculated to obtain the delta beta (Δβ) value. We filtered for highly differentially methylated sites (|delta beta (Δβ)| > 10%) using linear regression modeling. Genes are listed here with their nominal p value, Δβ, and gene annotations.

Supplemental Table 25: Gene Ontology Analysis for BOS Blood RNA-seq and DNA Methylation Integration

Integration of DEGs from our BOS RNA-seq blood (Supplemental Table 12) and BOS blood DNA methylation (Supplemental Table 21) were analyzed using clusterProfiler *v3.12.0* to identify gene set enrichments. clusterProfiler *v3.12.0* uses all genes from the Gencode hg38 annotation, version 31, as background. We filtered for gene ontologies with padj < 0.05 for significance.

Supplemental Table 26: Dysregulation of Wnt Signaling Genes in BOS RNA-seq and ATACseq Datasets

Log_2FoldChange and Bonferroni adjusted p values for RNA-seq and ATAC-seq analyses of BOS patient samples are listed for Wnt signaling genes (KEGG pathway).

Unedited and uncropped blots for Manuscript

JCI Insight Revision #167744-INS-RG-TR-2

Full Unedited Gel for Figure 1E

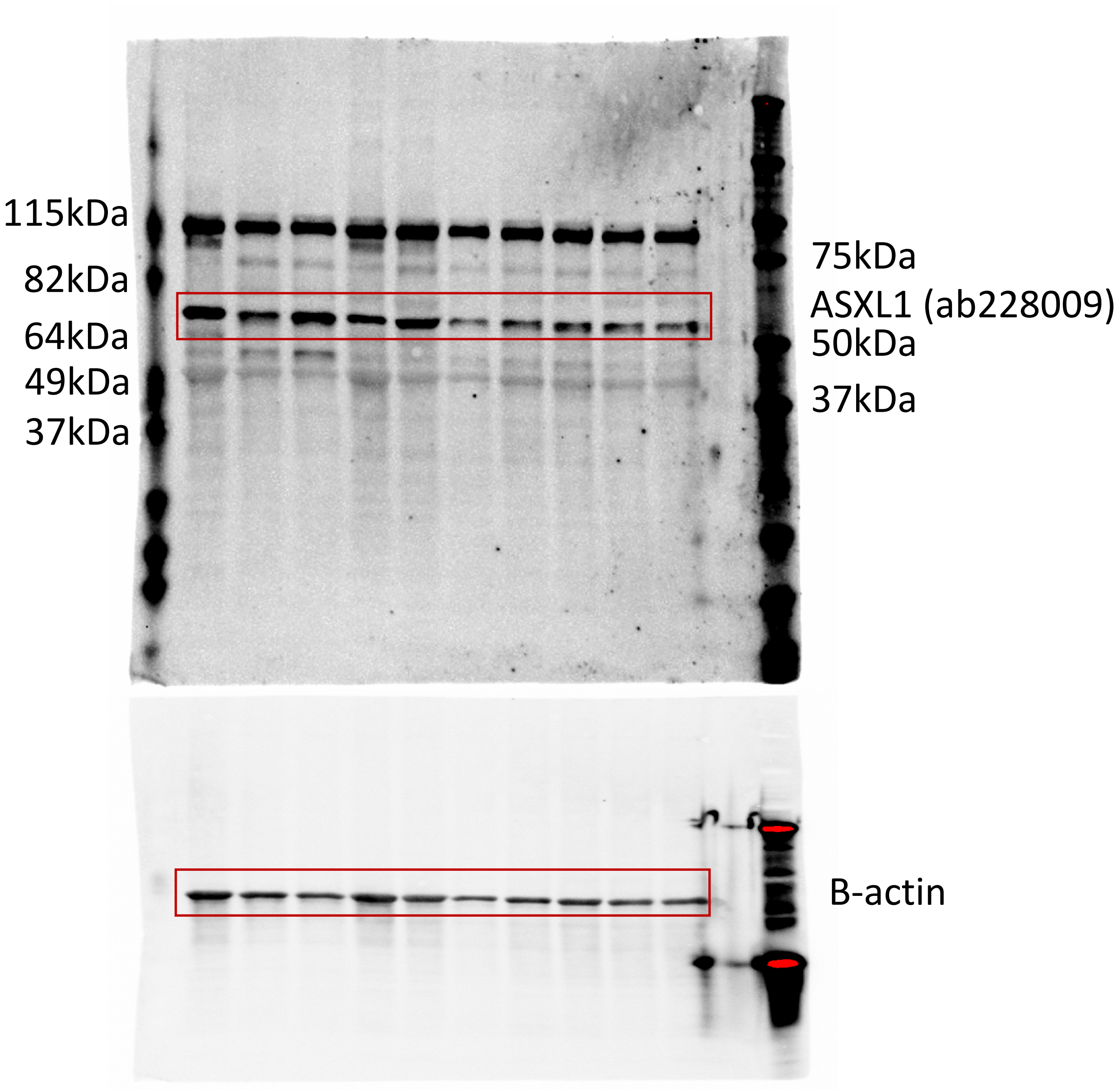

Sample ID (L to R):
1) Pt2
2) Pt3
3) Pt4
4) Pt14
5) Pt15
6) Ctrl1
7) Ctrl50
8) Ctrl51
9) Ctrl52
10) Ctrl53

Secondary: Goat Anti-Rabbit 680

Protein Ladder:

1.5ul Benchmark Pre-stained Protein Ladder (L)

1.5ul Precision Plus Pre-stained Protein Ladder (R)

Sample: 15ug whole cell lysate

Settings for gel: 130V 60 min

Settings for transfer: mixed mw

Full Unedited Gel for Supplemental Figure 4A

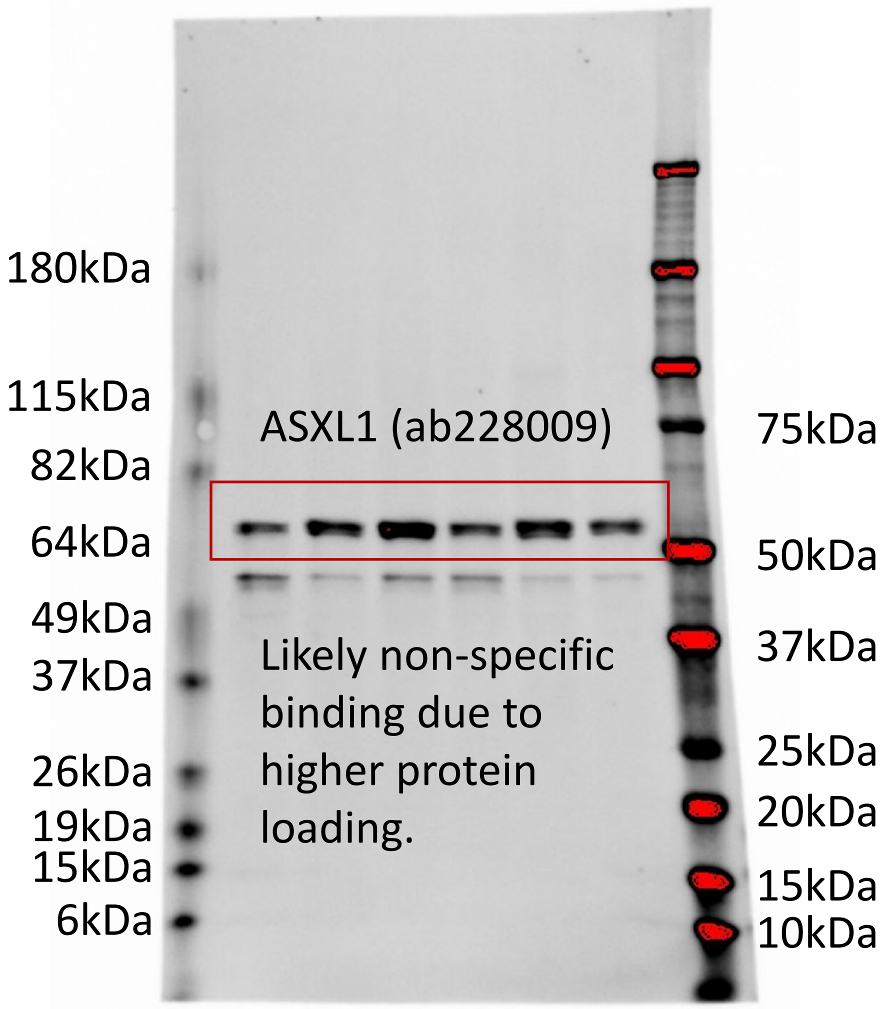

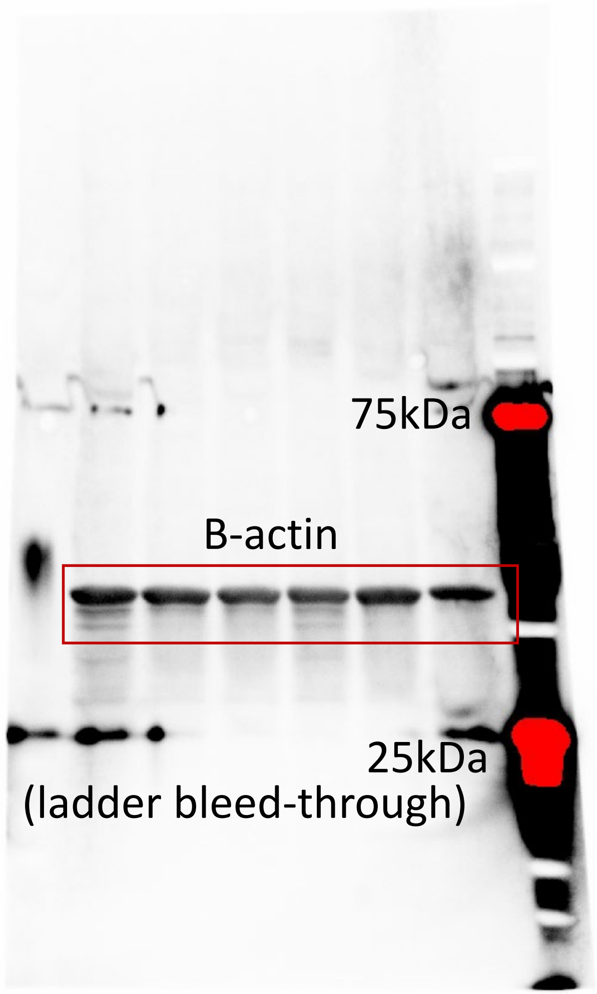

Sample ID (L to R):

1) Pt3
2) Pt4
3) Pt15
4) Ctrl1
5) Ctrll50
6) Ctrl51

Secondary: Goat Anti-Rabbit 680

Protein Ladder:

1.5ul Benchmark Pre-stained Protein Ladder (L)

1.5ul Precision Plus Pre-stained Protein Ladder (R)

Sample: 15ug whole cell lysate

Settings for gel: 130V 60 min

Settings for transfer: mixed mw

Full Unedited Gel for Supplemental Figure 4B

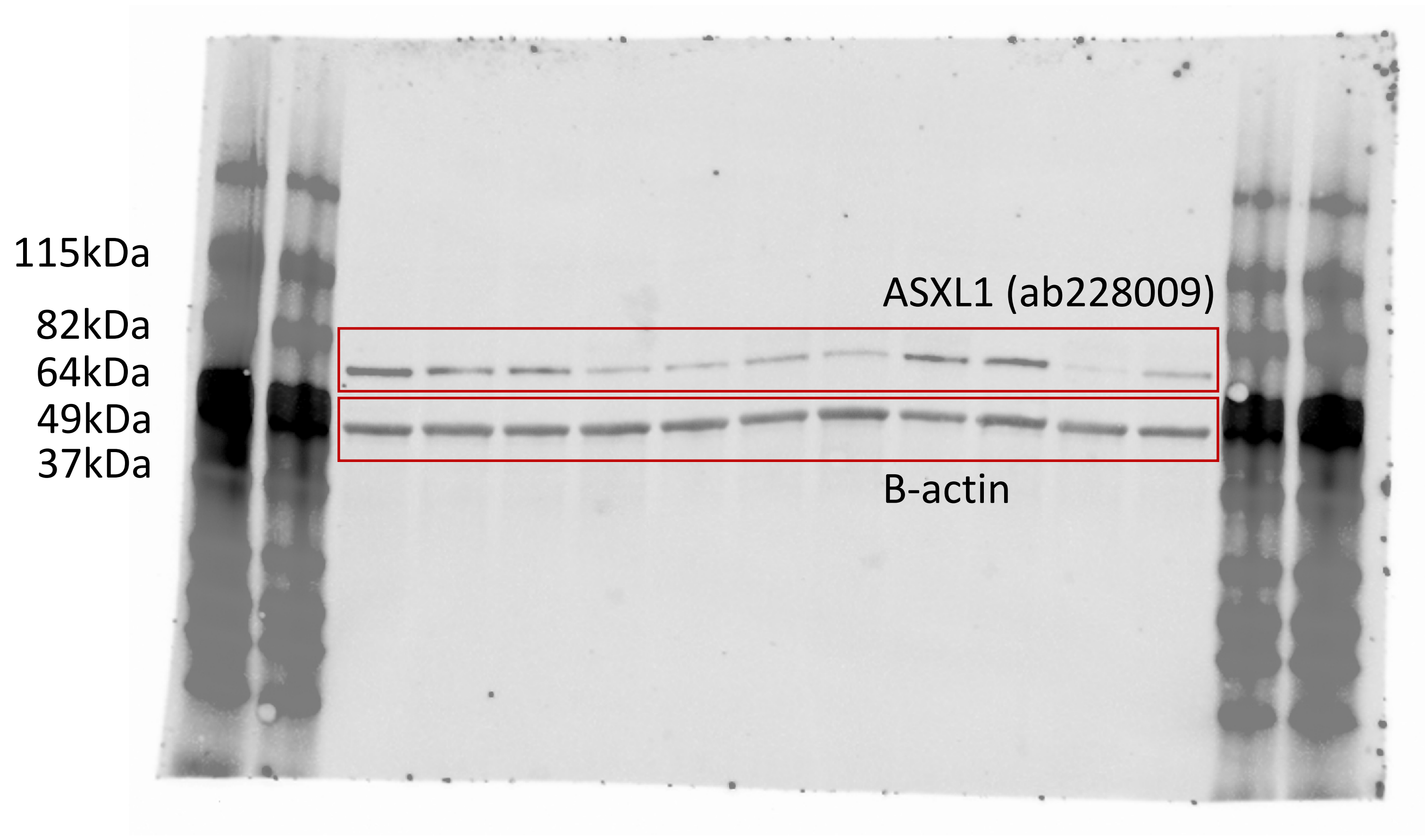

Sample ID (L to R):
1) Pt1
2) Pt2
3) Pt4
4) Pt6
5) Pt9
6) Pt12
7) Pt14
8) Pt15
9) Ctrl5
10) Ctrl8
11) Ctrl10

Secondary: Goat Anti-Rabbit 680

Protein Ladder:
2ul Benchmark Pre-stained Protein Ladder (R)
2ul Precision Plus Pre-stained Protein Ladder (R)

Sample: 15ug whole cell lysate
Settings for gel: 130V 60 min
Settings for transfer: mixed mw

Full Unedited Gel for Supplemental Figure 4C

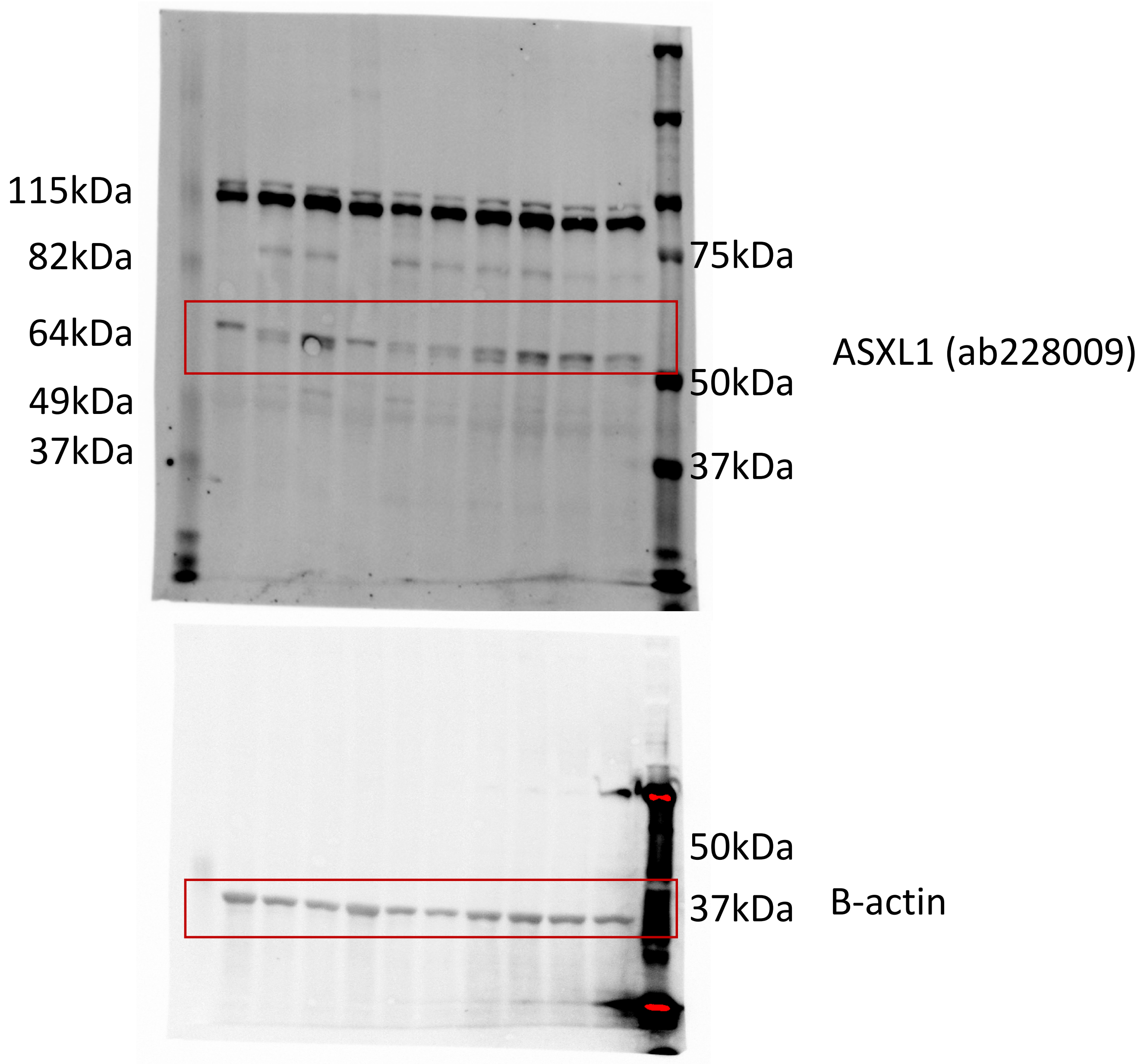

Sample ID (L to R):

1) Pt2
2) Pt3
3) Pt4
4) Pt14
5) Pt15
6) Ctrl1
7) Ctrl50
8) Ctrl51
9) Ctrl52
10) Ctrl53

Secondary: Goat Anti-Rabbit 680

Protein Ladder:

1.5ul Benchmark Pre-stained Protein Ladder (L)
1.5ul Precision Plus Pre-stained Protein Ladder (R)

Sample: 15ug whole cell lysate
Settings for gel: 130V 60 min
Settings for transfer: mixed mw

Full Unedited Gel for Supplemental Figure 4D

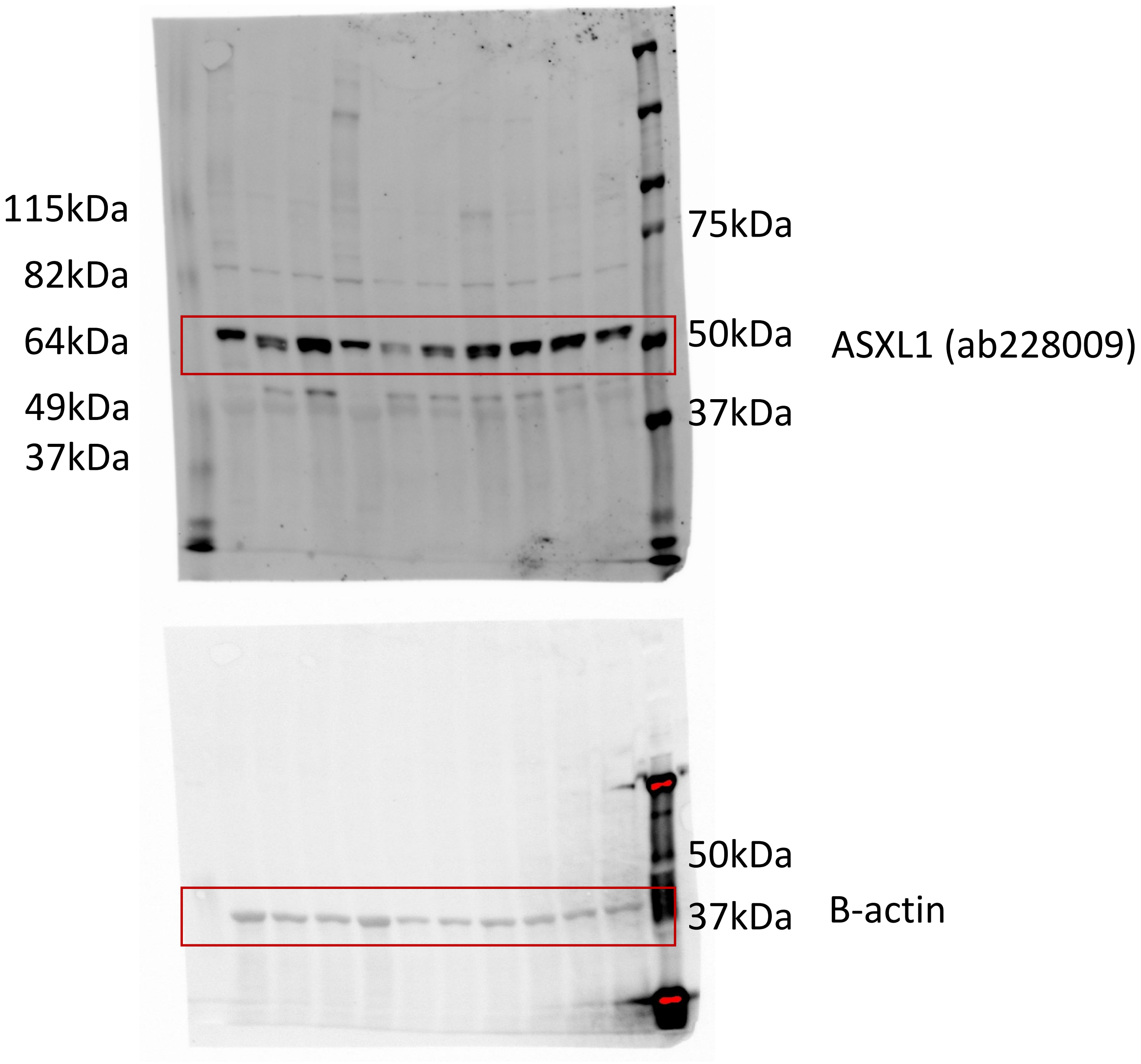

Sample ID (L to R):
1) Pt2
2) Pt3
3) Pt4
4) Pt14
5) Pt15
6) Ctrl1
7) Ctrl50
8) Ctrl51
9) Ctrl52
10) Ctrl53

Secondary: Goat Anti-Rabbit 680

Protein Ladder:

1.5ul Benchmark Pre-stained Protein Ladder (L)

1.5ul Precision Plus Pre-stained Protein Ladder (R)

Sample: 15ug whole cell lysate

Settings for gel: 130V 60 min

Settings for transfer: mixed mw

Full Unedited Gel for Figure 1F

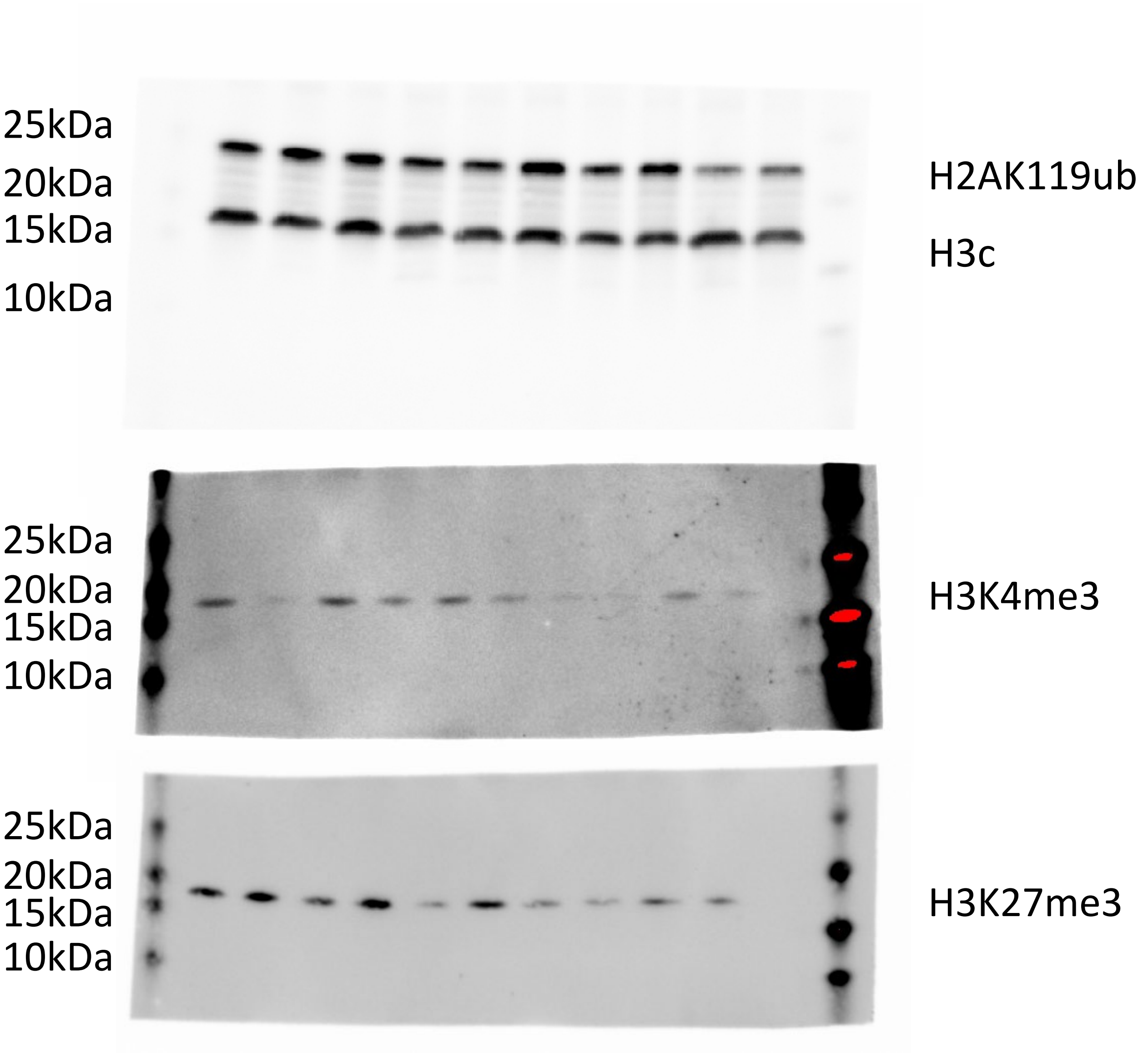

Sample ID (L to R):
1) Pt2
2) Pt3
3) Pt4
4) Pt14
5) Pt15
6) Ctrl1
7) Ctrl2
8) Ctrl3
9) Ctrl50
10) Ctrl51

Secondary: Goat Anti-Rabbit 680

Protein Ladder:

0.5ul Benchmark Pre-stained Protein Ladder (L)

0.5ul Precision Plus Pre-stained Protein Ladder (R)

Sample: 1ug histones

Settings for gel: 130V 60 min

Settings for transfer: mixed mw

Full Unedited Gel for Supplemental Figure 5A

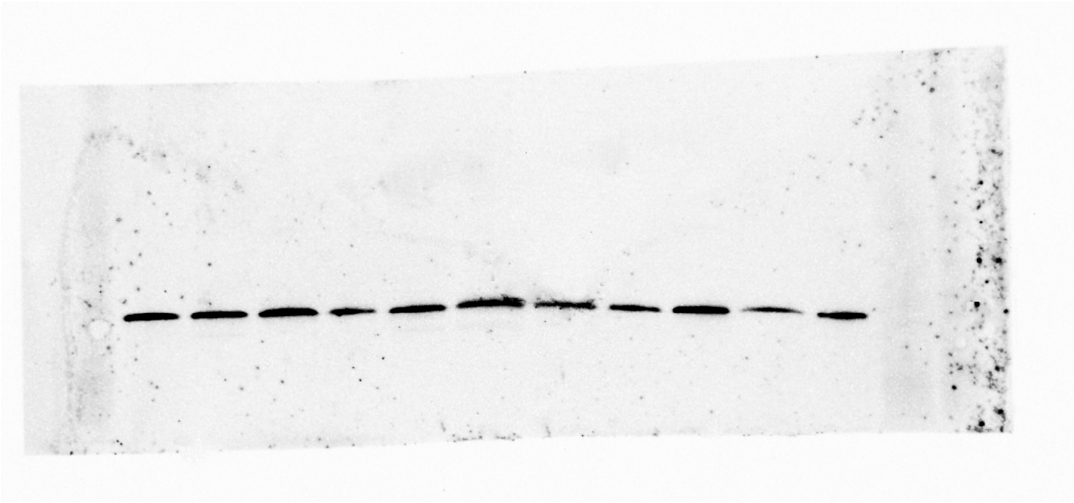

H3c

25kDa
20kDa
15kDa

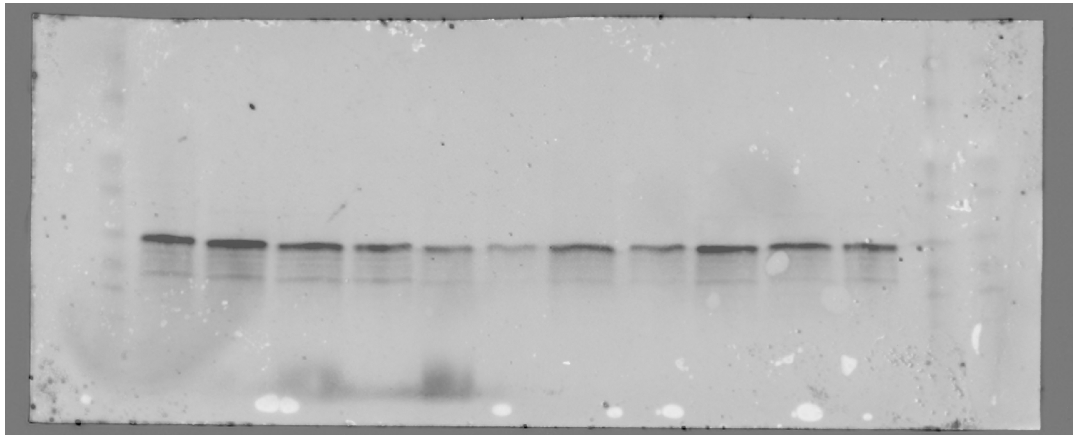

H3K4me3

Sample ID (L to R):
1) Pt1
2) Pt2
3) Pt4
4) Pt6
5) Pt9
6) Pt12
7) Pt14
8) Pt15
9) Ctrl5
10) Ctrl8
11) Ctrl10

Secondary: Goat Anti-Rabbit 680

Protein Ladder:
0.5ul Benchmark Pre-stained Protein Ladder (L)
0.5ul Benchmark Pre-stained Protein Ladder (R)

Sample: 1ug histones

Settings for gel: 130V 60 min

Settings for transfer: mixed mw

Full Unedited Gel for Supplemental Figure 5B

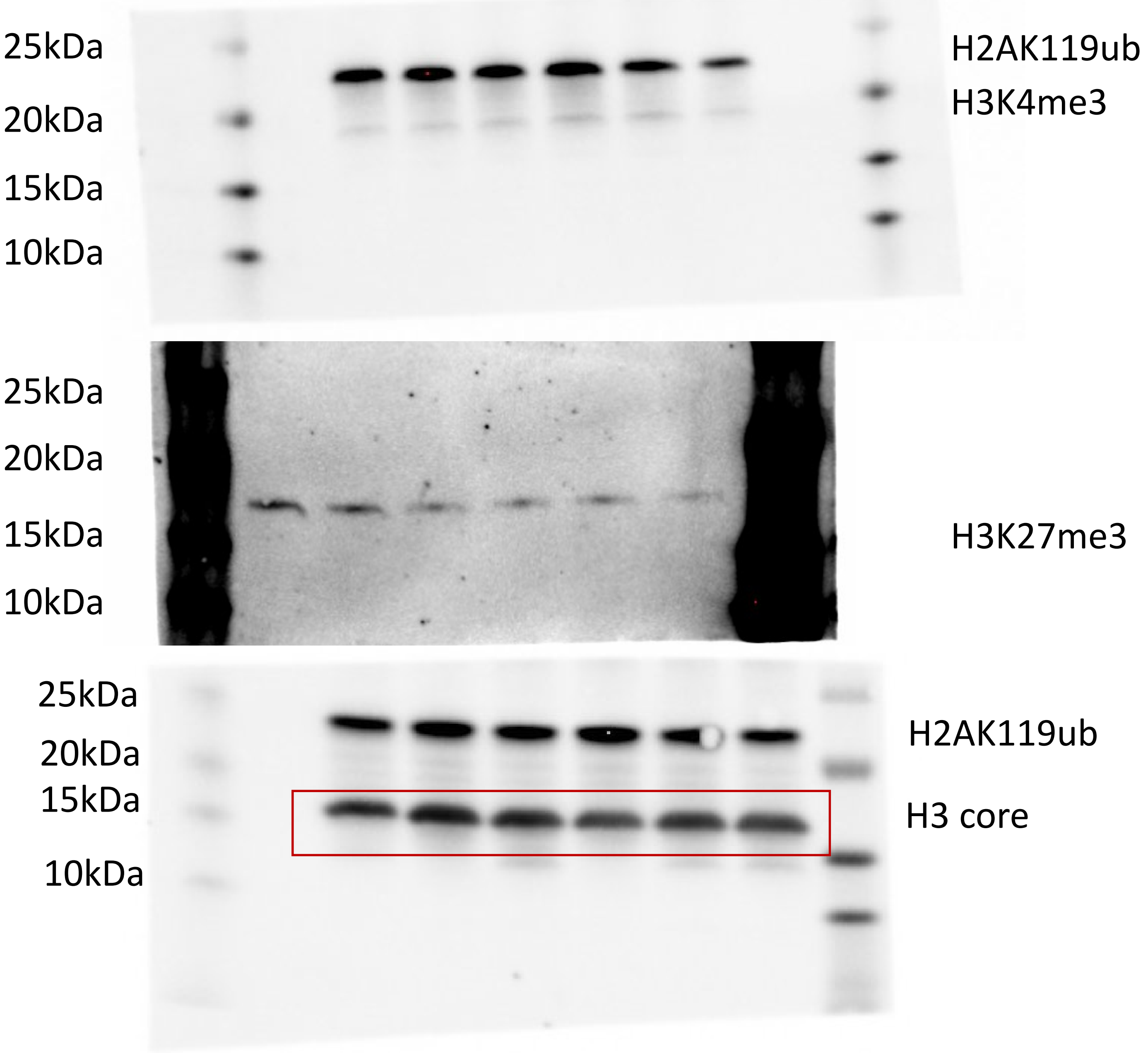

Sample ID (L to R):
1) Pt3
2) Pt4
3) Pt15
4) Ctrl1
5) Ctrl50
6) Ctrl51

Secondary: Goat Anti-Rabbit 680

Protein Ladder:
0.5ul Benchmark Pre-stained Protein Ladder (L)
0.5ul Precision Plus Pre-stained Protein Ladder (R)
Sample: 1ug histones
Settings for gel: 130V 60 min
Settings for transfer: mixed mw

Full Unedited Gel for Supplemental Figure 5C

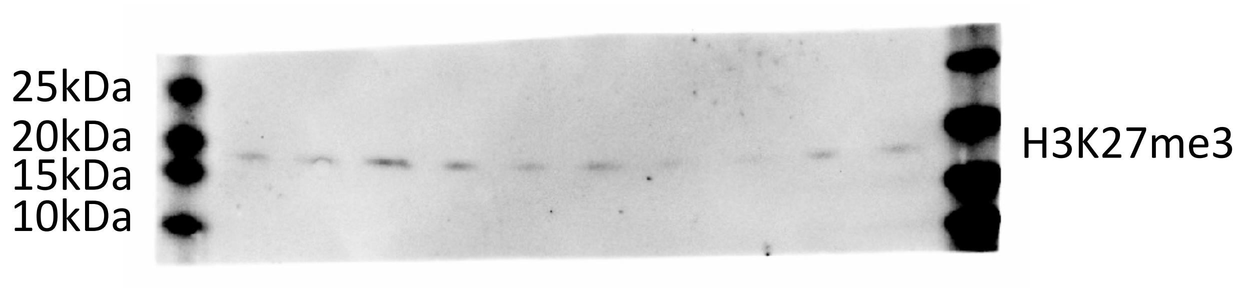

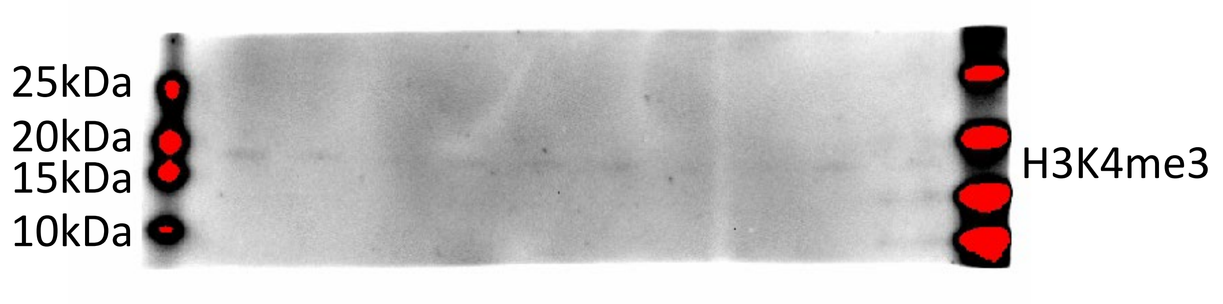

Sample ID (L to R):

1) Pt2
2) Pt3
3) Pt4
4) Pt14
5) Pt15
6) Ctrl1
7) Ctrl2
8) Ctrl3
9) Ctrl50
10) Ctrl51

Secondary: Goat Anti-Rabbit 680

Protein Ladder:

0.5ul Benchmark Pre-stained Protein Ladder (L)
0.5ul Precision Plus Pre-stained Protein Ladder (R)

Sample: 1ug histones
Settings for gel: 130V 60 min
Settings for transfer: mixed mw

Full Unedited Gel for Figure 5B

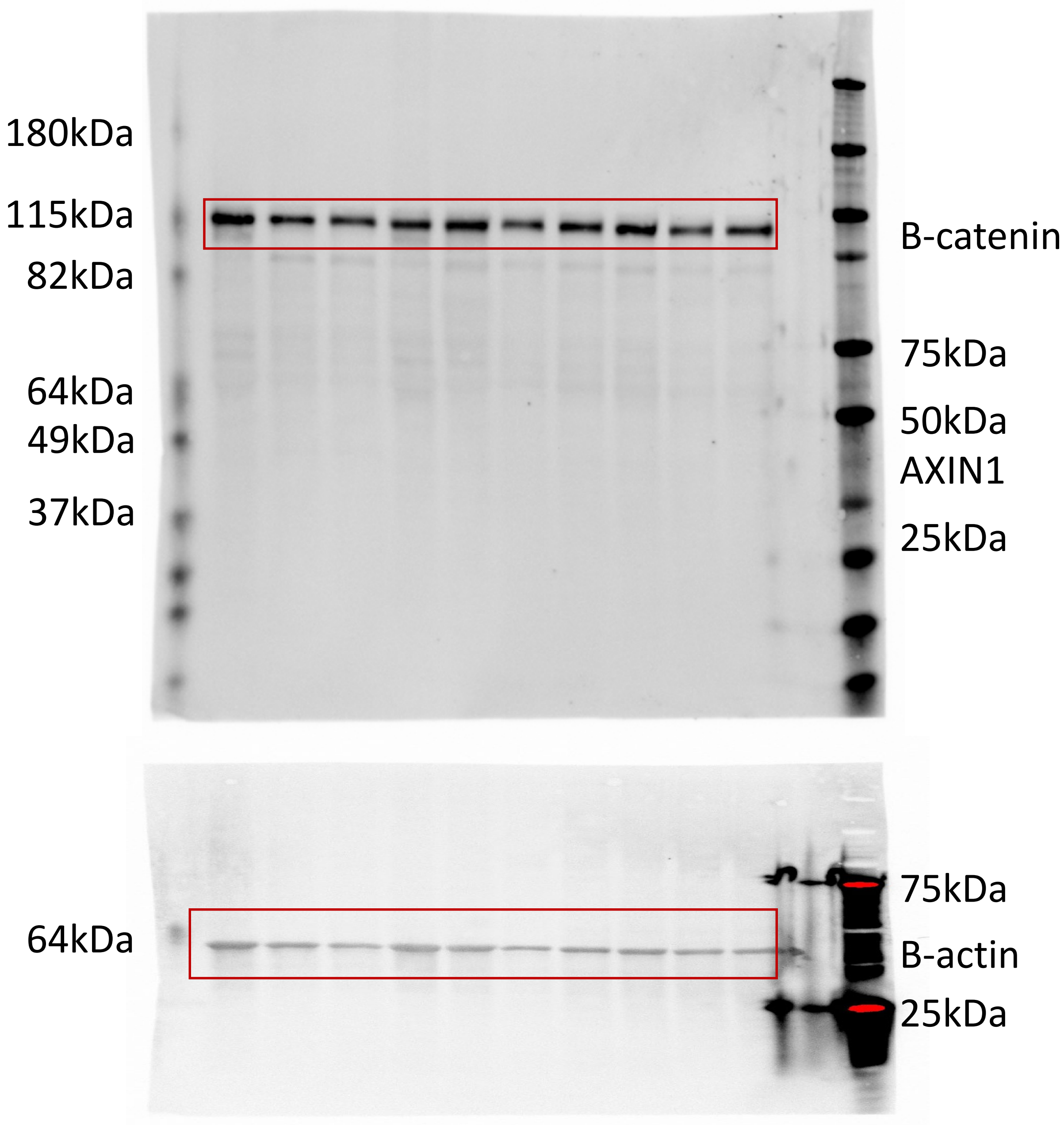

Sample ID (L to R):

1) Pt2
2) Pt3
3) Pt4
4) Pt14
5) Pt15
6) Ctrl1
7) Ctrl50
8) Ctrl51
9) Ctrl52
10) Ctrl53

Secondary: Goat Anti-Rabbit 680

Protein Ladder:

1.5ul Benchmark Pre-stained Protein Ladder (L)

1.5ul Precision Plus Pre-stained Protein Ladder (R)

Sample: 15ug whole cell lysate

Settings for gel: 130V 60 min

Settings for transfer: mixed mw

Full Unedited Gel for Figure 5B

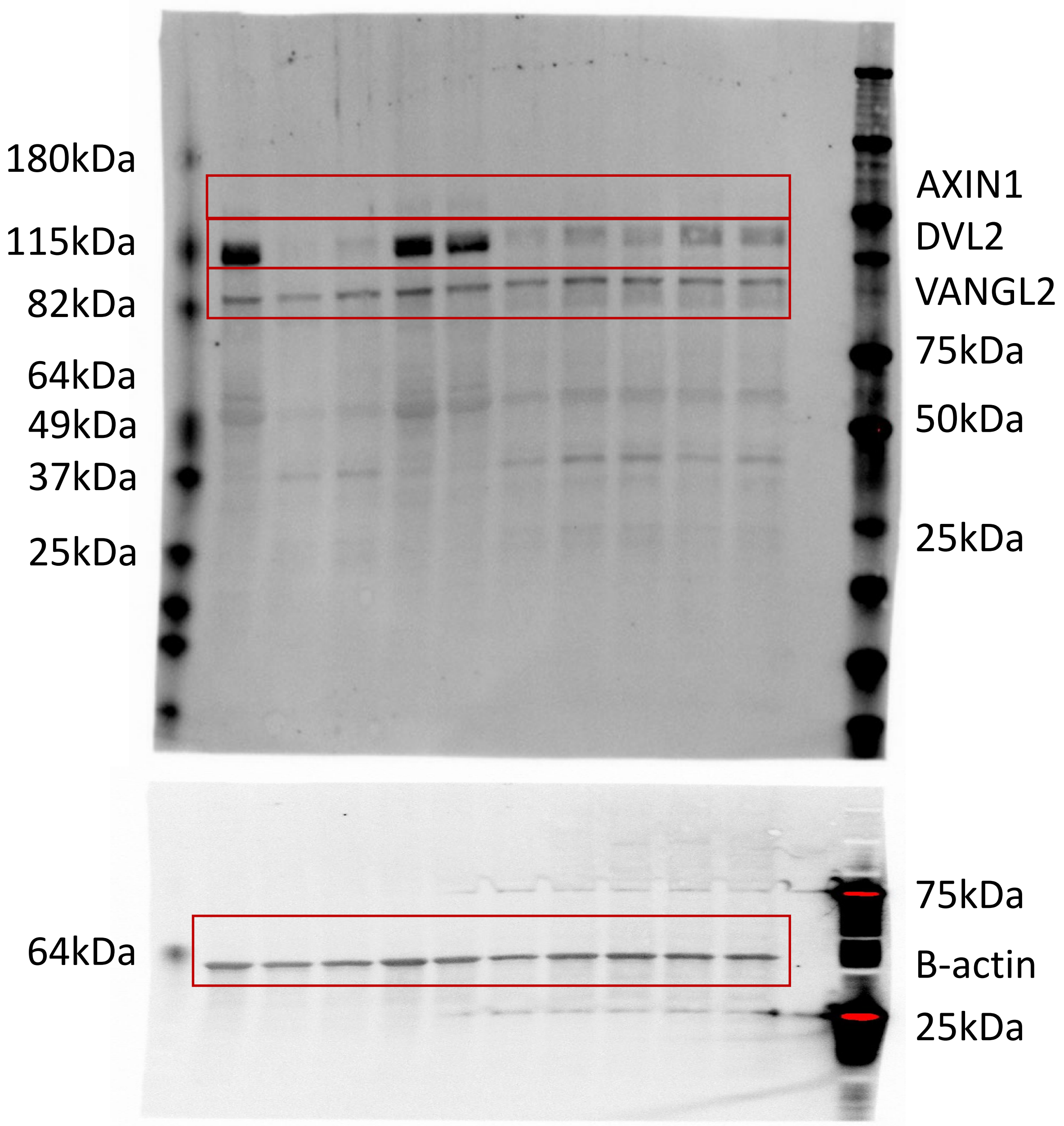

Sample ID (L to R):

1) Pt2
2) Pt3
3) Pt4
4) Pt14
5) Pt15
6) Ctrl1
7) Ctrl50
8) Ctrl51
9) Ctrl52
10) Ctrl53

Secondary: Goat Anti-Rabbit 680

Protein Ladder:

1.5ul Benchmark Pre-stained Protein Ladder (L)

1.5ul Precision Plus Pre-stained Protein Ladder (R)

Sample: 15ug whole cell lysate

Settings for gel: 130V 60 min

Settings for transfer: mixed mw

Full Unedited Gel for Figure 5B

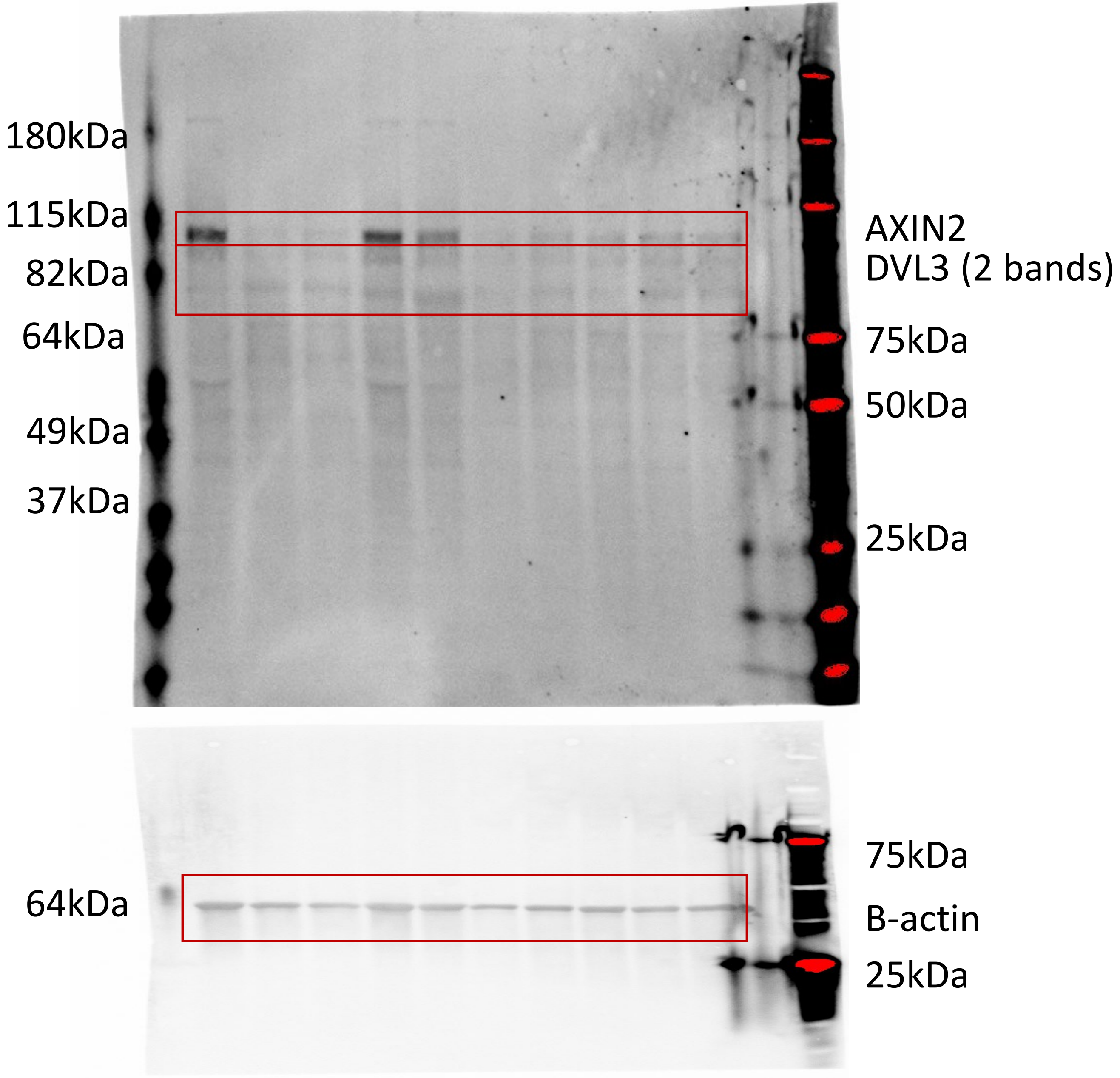

Sample ID (L to R):

1) Pt2
2) Pt3
3) Pt4
4) Pt14
5) Pt15
6) Ctrl1
7) Ctrl50
8) Ctrl51
9) Ctrl52
10) Ctrl53

Secondary: Goat Anti-Rabbit 680

Protein Ladder:

1.5ul Benchmark Pre-stained Protein Ladder (L)
1.5ul Precision Plus Pre-stained Protein Ladder (R)

Sample: 15ug whole cell lysate
Settings for gel: 130V 60 min
Settings for transfer: mixed mw

Full Unedited Gel for Supplemental Figure 16

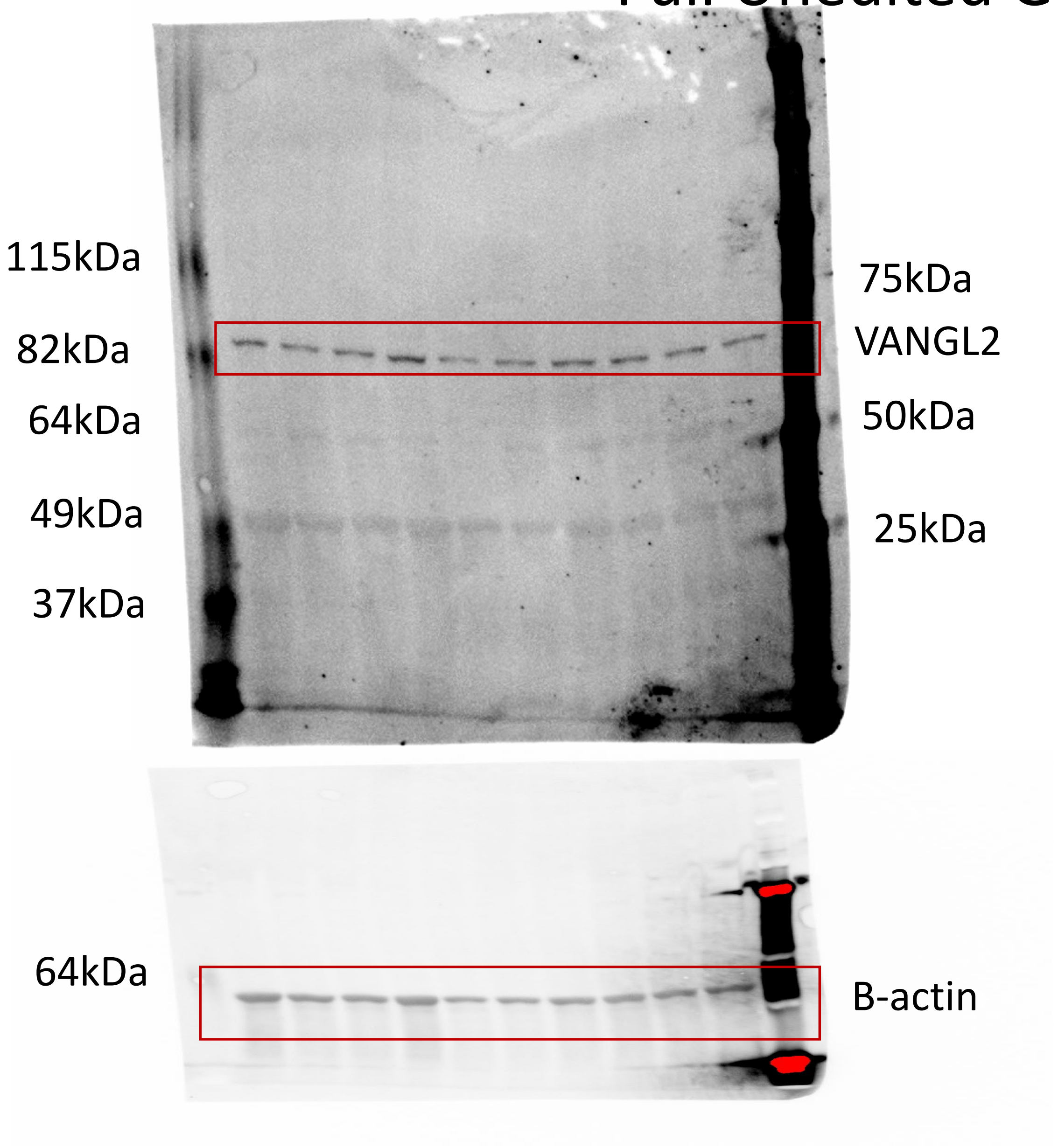

Sample ID (L to R):

1) Pt2
2) Pt3
3) Pt4
4) Pt14
5) Pt15
6) Ctrl1
7) Ctrl50
8) Ctrl51
9) Ctrl52
10) Ctrl53

Secondary: Goat Anti-Rabbit 680

Protein Ladder:

1.5ul Benchmark Pre-stained Protein Ladder (L)
1.5ul Precision Plus Pre-stained Protein Ladder (R)

Sample: 15ug whole cell lysate
Settings for gel: 130V 60 min
Settings for transfer: mixed mw

Full Unedited Gel for Supplemental Figure 16

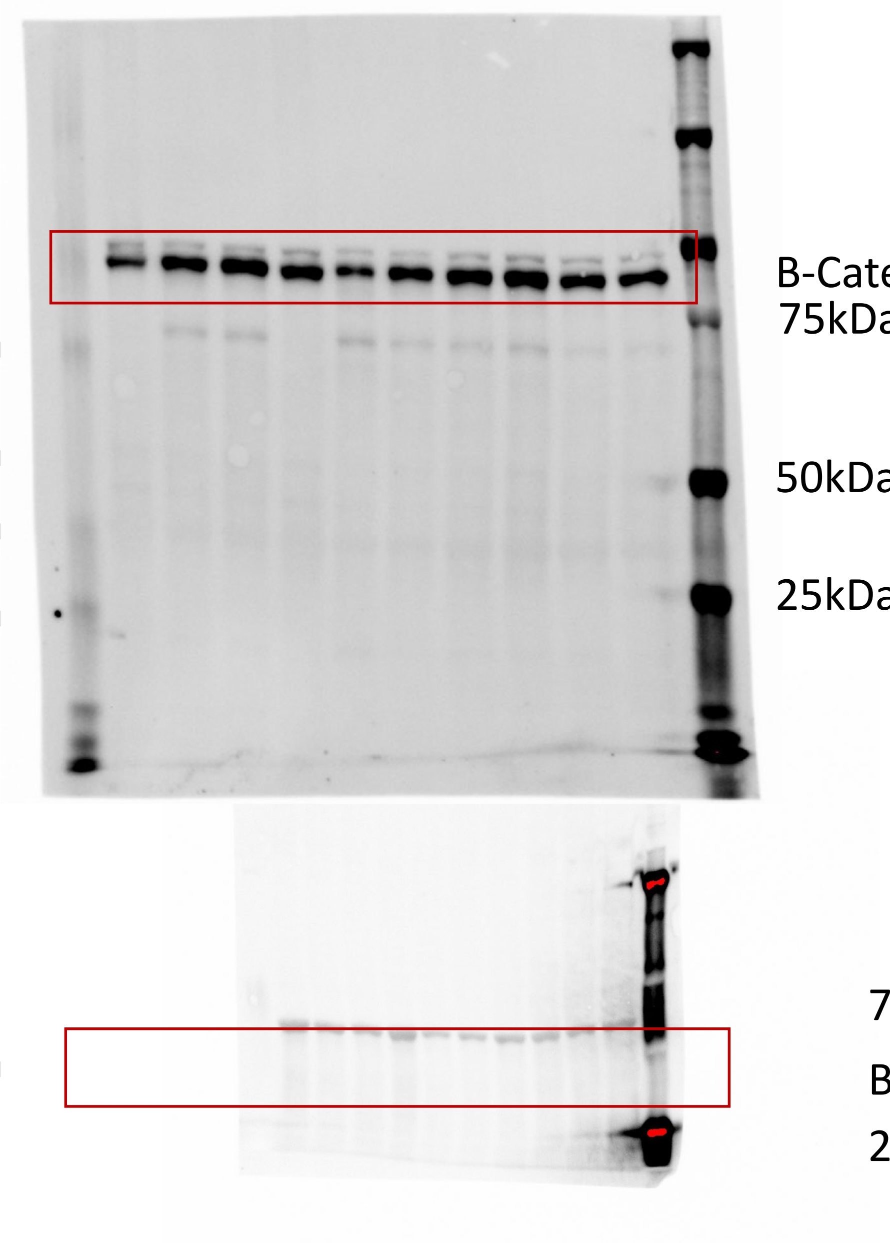

Sample ID (L to R):

1) Pt2
2) Pt3
3) Pt4
4) Pt14
5) Pt15
6) Ctrl1
7) Ctrl50
8) Ctrl51
9) Ctrl52
10) Ctrl53

Secondary: Goat Anti-Rabbit 680

Protein Ladder:

1.5ul Benchmark Pre-stained Protein Ladder (L)
1.5ul Precision Plus Pre-stained Protein Ladder (R)

Sample: 15ug whole cell lysate
Settings for gel: 130V 60 min
Settings for transfer: mixed mw

Full Unedited Gel for Supplemental Figure 16

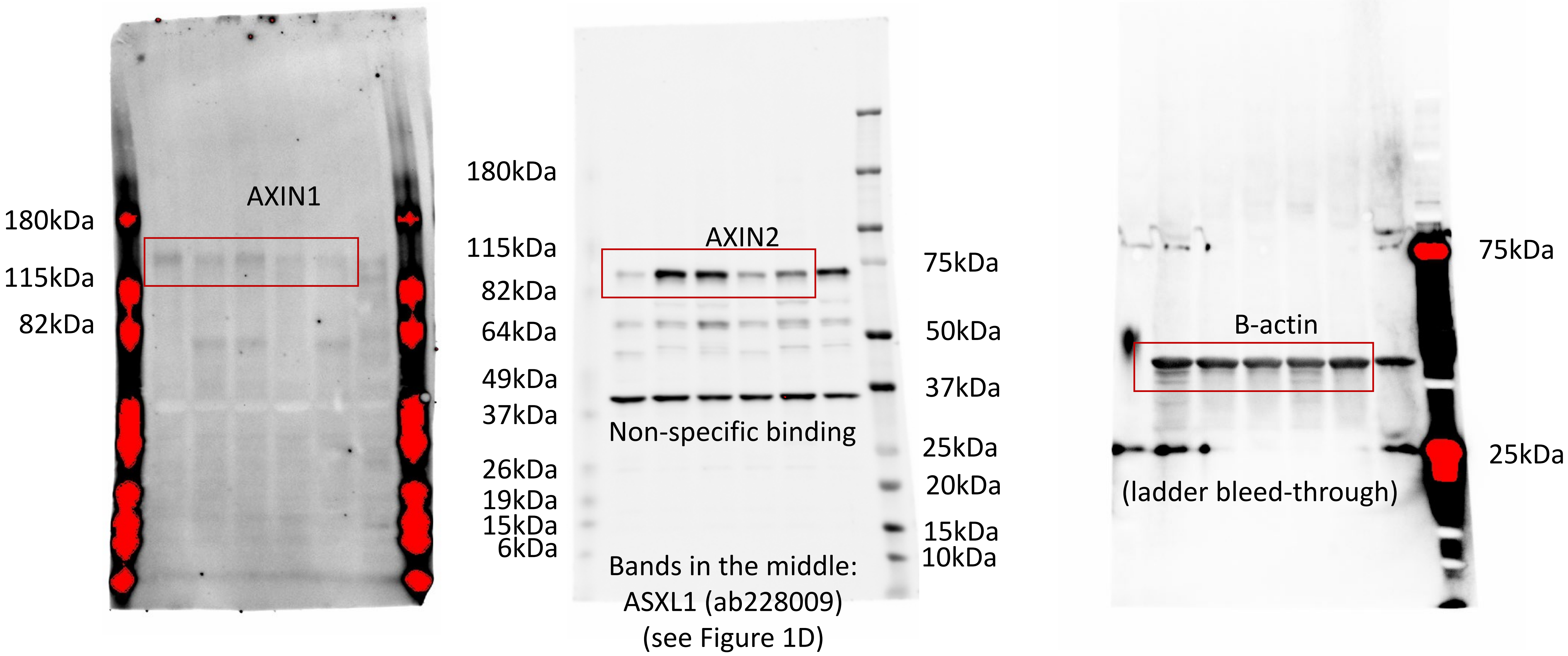

CACO2 cell lysate was used in the last lane in the AXIN1 blot, so was therefore cropped out in the fibroblast images
The last lane was cropped out of the AXIN2 blot to match other Wnt signaling blots that were run with CACO2 as the last lane

Full Unedited Gel for Supplemental Figure 16

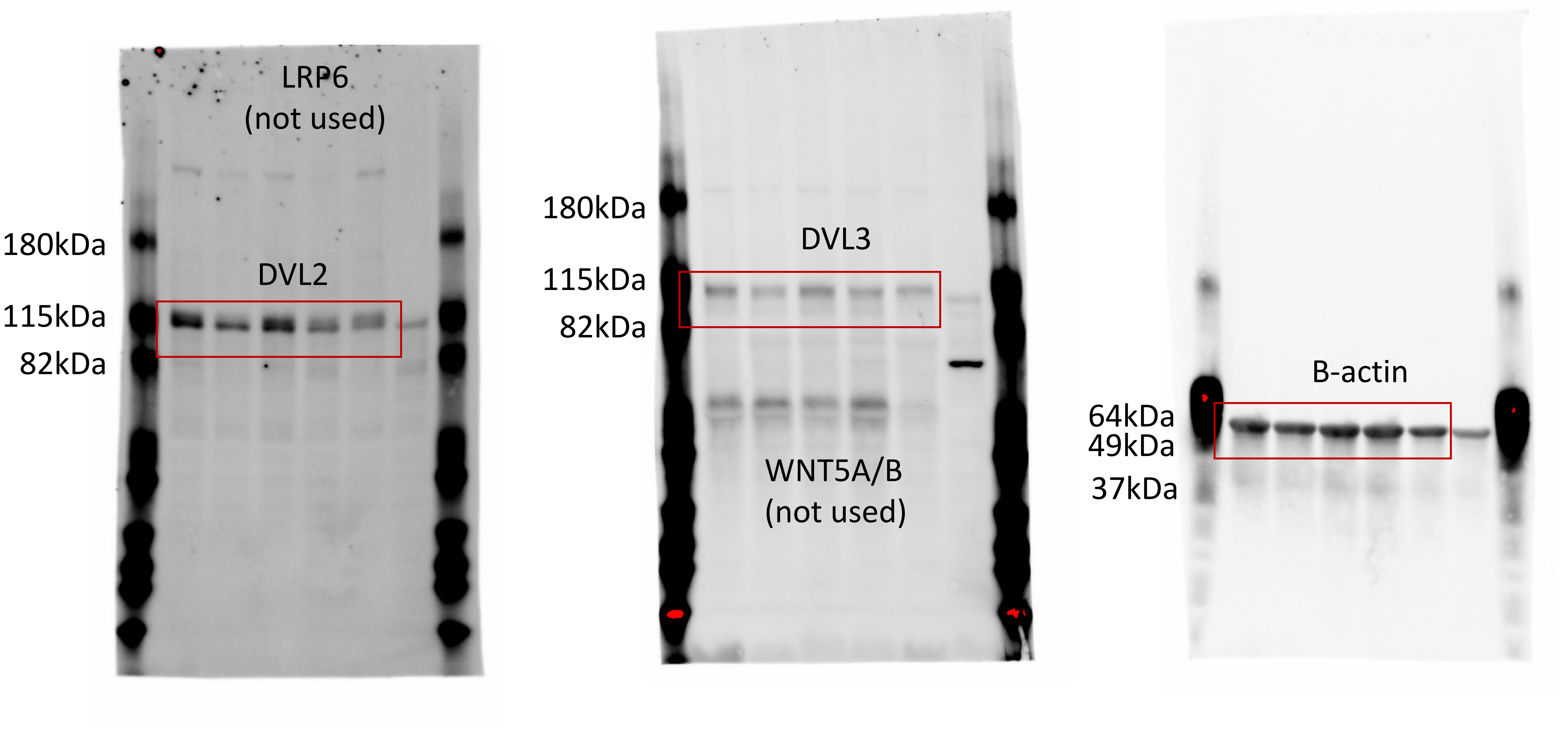

CACO2 cell lysate was used in the last lane, so was therefore cropped out in the fibroblast images
